# Modelling the influence of environmental factors on the acoustic presence of blue whale populations in the southern Indian Ocean

**DOI:** 10.1038/s41598-025-02941-9

**Published:** 2025-07-02

**Authors:** Mathilde Michel, Baptiste Alglave, Maxime Olmos, Maëlle Torterotot, Auriane Virgili, Salomé Martin-Marin, Jean-Yves Royer, Flore Samaran

**Affiliations:** 1 ENSTA, IPP, CNRS Lab-STICC, 29200 Brest, France; 2 Université Bretagne Sud, Lab-STICC, 56000 Vannes, France; 3 DECOD (Ecosystem Dynamics and Sustainability), L’Institut Agro, Ifremer, INRAE, 29280 Plouzané, France; 4 Share the Ocean, 56870 Larmor-Baden, France; 5 Geo-Ocean, CNRS, Université de Brest, Ifremer, 29280 Plouzané, France

**Keywords:** Spatio-temporal modelisation, Blue whale, Acoustic recording, Animal migration, Marine biology, Acoustics, Ecosystem ecology

## Abstract

Blue whales in the Indian Ocean have been severely depleted by previous extensive commercial whaling. A good understanding of their spatio-temporal distribution is crucial for conservation. The songs of three blue whale acoustic populations - Antarctic blue whales (*Balaenoptera musculus intermedia*, ANT BW) and pygmy blue whales (*B. musculus brevicauda*) from the Southeast (SEIO PBW) and Southwest Indian Ocean (SWIO PBW) - were analyzed using 13 years of passive acoustic recordings from 10 sites in the southwest Indian Ocean. Although blue whale vocalizations comprise both songs and non-song calls (e.g., D-calls), the present study concentrates on the examination of songs. Generalized additive models (GAMs) were used to relate acoustic presence, measured by the number of positive minutes per day (averaged weekly), to environmental drivers such as sea surface temperature (SST), chlorophyll-a concentrations, and sea ice extent. These models allowed predictions of blue whale acoustic presence across the region. Empirical orthogonal functions (EOFs) were applied for dimensionality reduction to identify key habitats, including the Kerguelen Plateau and Madagascar Basin, which may serve as important feeding and resting zones based on acoustic presence and environmental data. Antarctic blue whales were predominantly detected in austral winter and spring, associated with lower SST and higher chlorophyll-a. In contrast, SEIO and SWIO pygmy blue whales were more frequent in summer and autumn, with some overlap suggesting ecological interactions. These findings lay the groundwork for targeted conservation efforts to protect critical blue whale habitats in a rapidly changing ocean.

## Introduction

Blue whale (*Balaenoptera musculus*) populations in the southern hemisphere have been severely depleted by previous extensive commercial whaling. As a consequence, the International Whaling Commission (IWC) established the Indian Ocean Whale Sanctuary (IOWS) in 1979, prohibiting commercial whaling in the region^1^. The rare nature of blue whales, combined with the vast and remote expanses of their habitat, complicates efforts to observe them directly. Given these challenges, passive acoustic monitoring has emerged as the most effective method to study these cetaceans, allowing researchers to track their presence and behavior through sounds they produced^2–7^. This approach is particularly appropriate for blue whales, which are highly vocal animals characterized by long low-frequency sounds^8^.

Blue whale sounds are broadly categorized into two types: songs and non-song calls. Songs are complex long-duration calls and are made up of repeated units organized in series, separated by regular intervals (intercall interval, ICI)^5^. They are recorded in all seasons in both feeding and breeding areas and are characterized by a stereotype time-frequency structure, allowing differentiation between populations^3,4^. In contrast, non-song calls, such as D-calls, are shorter, frequency-modulated vocalizations typically associated with foraging behavior^9^. D-calls exhibit highly variable frequencies and are produced by both males and females, without a clear geographic variation in their time-frequency shape^10–13^.

Temporally stable differences in songs provide characteristics that can be used to distinguish the two subspecies and to define several acoustic populations of pygmy blue whales in the Indian Ocean^14^. Although specific to each subspecies and acoustic population, blue whale songs share similar acoustic properties: the duration of song units exceeds 15 seconds, their amplitude is greater than 180 dB re 1 $\upmu$Pa at 1 m, and their pitch ranges from 20 to 100 Hz. Songs can be composed of multiple units, depending on the subspecies or acoustic population, and intercall intervals are periodic and do not exceed a few minutes. The year-round recording of blue whale vocal activity in various locations suggests that individuals produce acoustic emissions continuously throughout the year at the population level^3,4,7,15^. Furthermore, long-term analyzes have revealed a gradual decline in the frequency of blue whale calls in recent decades, although the underlying causes remain uncertain^16^. The differences in vocal repertoire between the blue whale subspecies and the acoustic populations of pygmy blue whales provide a unique means of tracking their migratory movements throughout an ocean basin^7,15,17,18^.

The southern region of the Indian Ocean hosts seasonally several acoustic populations of blue whales, including different subspecies and acoustic populations. Two subspecies are regularly present in this area: the Antarctic blue whale (*B. m. intermedia*) and the pygmy blue whale (*B. m. brevicauda*)^19^. There are notable differences in the way populations use their habitats, each of which exhibits distinct migration patterns. Antarctic blue whales (ANT BW) follow a seasonal migration from subantarctic regions in austral autumn to subtropical areas in austral winter, before returning to subantarctic waters in austral spring (September to November)^2,3,5,20^.

The acoustic populations of pygmy blue whales in the Southeast Indian Ocean (SEIO PBW) and the Southwest Indian Ocean (SWIO PBW) show distinct distribution patterns within the Indian Ocean and are predominantly observed during the austral autumn and winter seasons^3,4,7,15^. The SEIO PBW acoustic population is recorded mainly along the southwest coast of Australia, predominantly between March and May^21,22^, while the SWIO PBW acoustic population is found in regions further west, within the southwestern Indian Ocean^23–25^. However, recent findings reveal that these acoustic populations have been identified beyond their previously recognized distributional limits. SEIO PBW have been observed farther west than expected, in proximity to Kerguelen Island^3,5,7^, while SWIO PBW songs have been detected further south and west, near the Crozet Archipelago and southwest of Amsterdam Island^3,7^.

Oceanographic parameters, including sea surface temperature, chlorophyll-a concentration, and sea ice extent, are critical in determining prey availability and subsequently influence whale migratory movements and foraging behavior. Previous research has concentrated primarily on modelling the associations between these environmental variables and the distribution of cetaceans on extensive spatial scales, predominantly using visual or aerial observation data^26–28^. Other studies have used passive acoustic data, but have been limited to smaller spatial and temporal scales, often focusing only on correlations between environmental variables and acoustic presence^29,30^. Building on this foundation, this study seeks to extend these analyses to a larger geographic scale and a longer time frame. By integrating passive acoustic monitoring with oceanographic data, we provide comprehensive predictions for the entire study region, thus enhancing our understanding of key blue whale habitats and supporting conservation efforts.

Specifically, this study aims to improve the characterization of blue whale habitat based on 13 years of acoustic recordings collected in the southern Indian Ocean. Using a generalized additive model (GAM) framework, we evaluated the influence of oceanographic covariates on the acoustic presence, defined as an acoustic intensity metric based on the number of positive detection minutes per day, averaged weekly. This modelling approach allows us to (i) identify key environmental drivers of whale distribution and assess their non-linear effects, and (ii) predict spatio-temporal variations in blue whale acoustic presence, including inter-annual and seasonal patterns. In a subsequent phase, we will delineate functional areas using Empirical Orthogonal Functions (EOFs), a spatio-temporal dimension reduction technique. The identified spatial and temporal patterns are then compared with the existing literature to ensure alignment with established knowledge. These results provide valuable information for the conservation of this iconic species.

## Methods

### Area Description

The study area in the southern Indian Ocean extends from 20$^{\circ }$S to 60$^{\circ }$S latitude and from 45$^{\circ }$E to 90$^{\circ }$E longitude. In this region, the SST varies significantly, ranging from about 28 °C at lower latitudes to nearly 0 °C near the South Pole. This gradient is influenced by the separation of the water masses at the subantarctic and polar fronts. The subantarctic front, at about 45$^{\circ }$S, separates warm mid-latitude waters from cooler Antarctic waters, improving biological productivity^31^. Further south, between 50$^{\circ }$S and 60$^{\circ }$S, the polar front separates the subantarctic from the polar waters, promoting the formation of sea ice^32^. Antarctic sea ice expands seasonally, peaking in the austral winter and covering much of the southern Indian Ocean. These variations correspond to the natural cycles of sea ice formation and melting dictated by the regional climate. The chlorophyll-a distribution shows spatial variability, with high concentrations in nutrient-rich colder waters. On the Kerguelen Plateau, concentrations peak at 3.96 mg/m$^{3}$ due to nutrient accumulation and upwelling^33^. In contrast, lower latitudes have lower concentrations, around 0.02 mg/m$^{3}$, due to subsurface features that affect currents and nutrient transport^34^. Phytoplankton blooms on the polar front are highly variable in space and time, especially in December, and are associated with increased solar radiation and an increase in SST in the southern ocean^35^.

### Acoustic data collection

**Fig. 1:**
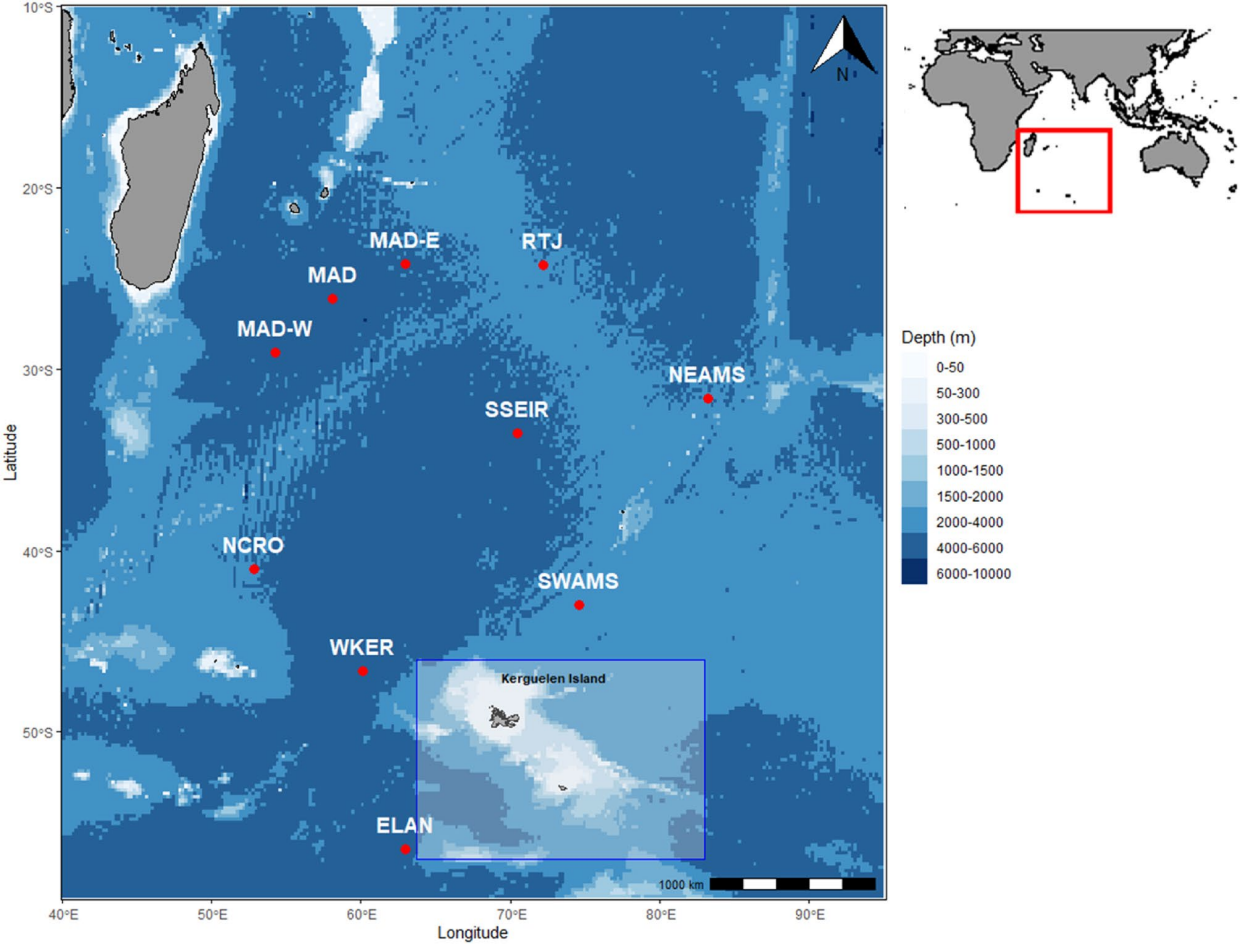
Locations of the mooring sites of the OHA-SIS-BIO hydrophone network. The blue square represents the Kerguelen Plateau region. The map was created using R (version 4.1.2; https://www.r-project.org/)^51^.

The acoustic data used in this study were collected by a wide network of autonomous hydrophones called the Hydro-Acoustic Observatory of Seismicity and Biodiversity (OHA-SIS-BIO)^36^. This network was deployed in the southern Indian Ocean from December 2009 to February 2023 (see Fig. 1). Initially and until 2016, it included five sites : south of la Réunion Island (MAD), north of the Crozet Archipelago (NCRO), west of Kerguelen Island (WKER), southwest (SWAMS) and northeast (NEAMS) of the Saint-Paul and Amsterdam Islands. An additional site south of the Southeast Indian Ridge (SSEIR) was installed in 2014. In 2017, the geometry of the network was slightly modified to improve coverage in the northern regions and better pinpoint the location of southern blue whale feeding grounds. Five new sites were added: near the junction of the three Indian Ocean seafloor spreading ridges (RTJ), west (MAD-W) and east (MAD-E) of the Madagascar Basin, south of the southwest Indian ridge (SSWIR) and south of the Kerguelen Plateau (ELAN), while the MAD and NCRO sites were no longer deployed.

**Fig. 2:**
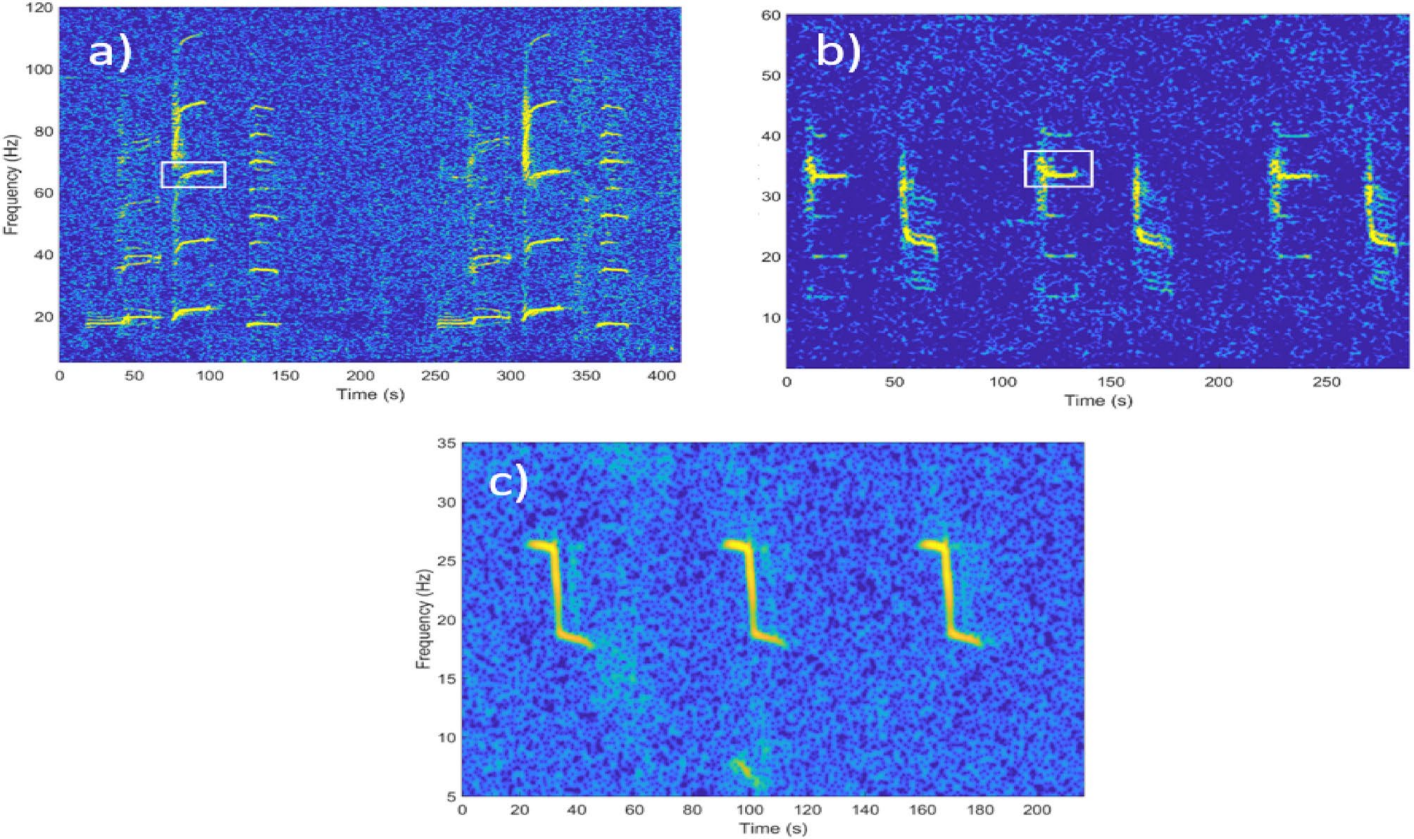
Spectrograms of (**a**) SEIO PBW songs (**b**) SWIO PBW songs, and (**c**) ANT BW songs from the southern Indian Ocean. The units selected for the detector are framed in white.

**Fig. 3:**
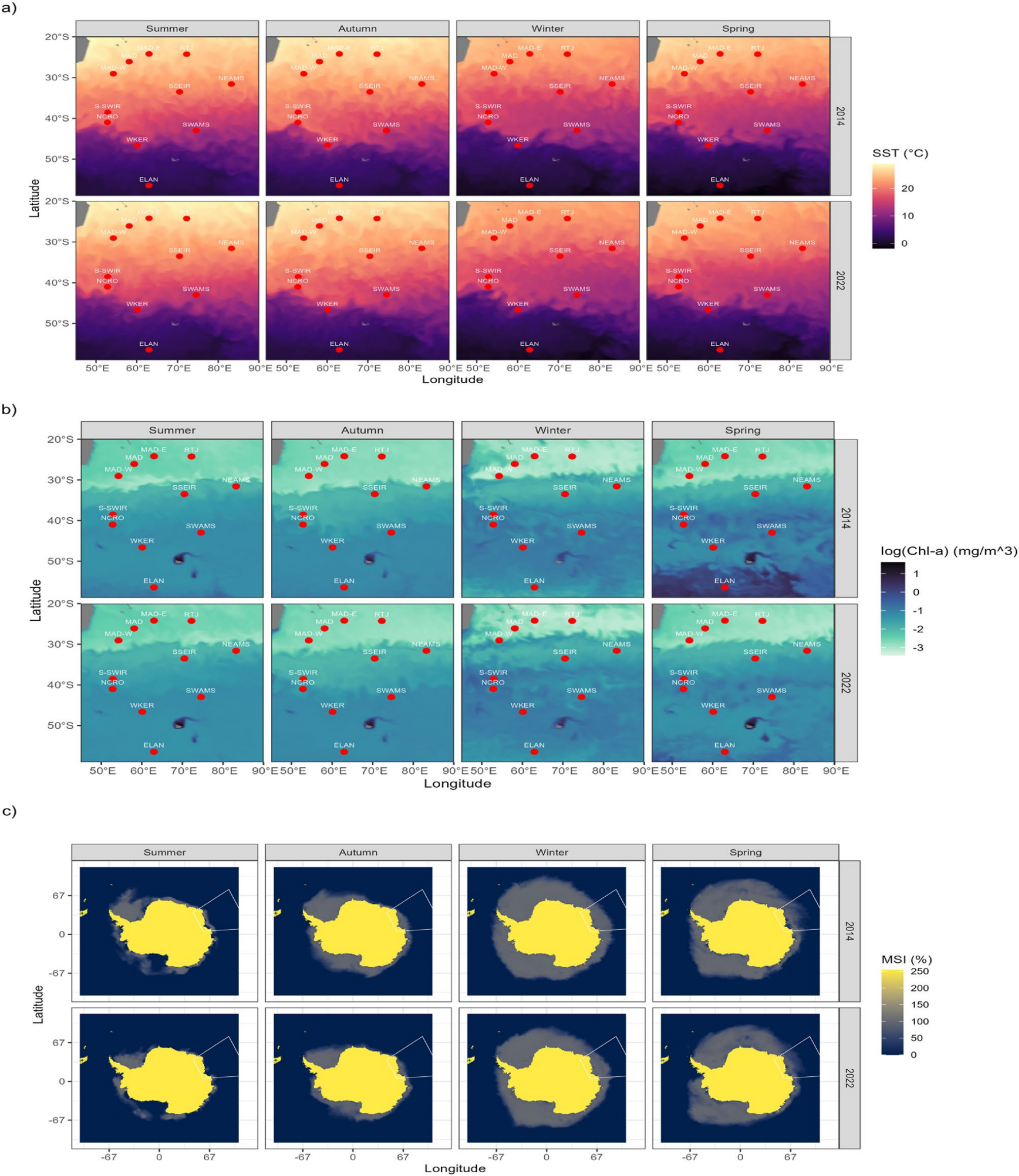
Monthly averages of covariates over the entire study area in the Indian Ocean for the four seasons, represented by January (summer), April (autumn), July (winter), and November (spring), for the years 2014 and 2022. The variables represented are: (**a**) SST (°C), (**b**) log (Chl-a) (mg/m$^{3}$), and (**c**) MSI (%). The red dots indicate the locations of the hydrophones used in this study.

**Table 1:** Summary of the hypotheses tested, the associated model configurations, and AIC values attributed to each model for each acoustic population: ANT BW, SEIO PBW, and SWIO PBW. Explained deviance (%) quantifies the proportion of variation explained by the model. $\Delta$AIC is the difference in the AIC score between the best model (*) and the model being compared.

Models	Spatio-temporal variations in DPM are explained by	Ecological hypothesis	Acoustic population	Distribution	Deviance explained (%)	AIC	$\Delta$AIC	Cross Validation ($R_{\text {Spearman}}$)
M0	Chlorophyll-a + sea surface temperature + mean sea ice	Only depends on environmental variables, does not take into account monthly and annual variations in acoustic presence	ANT BW	Tweedie	11.6	92019	3234	0.38
			SEIO PBW	Tweedie	43.5	34165	3748	0.49
			SWIO PBW	Tweedie	20.9	76628	6755	0.47
M1	M0 + Month and Year effect	Account for inter-annual and monthly changes in acoustic whale distribution	ANT BW	Tweedie	16.4	90471	1686	0.45
			SEIO PBW	Tweedie	52	31952	1535	0.52
			SWIO PBW	Tweedie	32.2	72155	2282	0.58
M2	M1 + longitude and latitude effects	Take into account the areas of distribution of species and migration patterns	ANT BW	Tweedie	21.2	88785	0*	0.51
			ANT BW	Negative binomial	20.9	88809	24	0.51
			SEIO PBW	Tweedie	57	30417	0*	0.54
			SEIO PBW	Negative binomial	56.9	31569	1152	0.54
			SWIO PBW	Tweedie	37.6	69873	0*	0.63
			SWIO PBW	Negative binomial	37.1	70617	744	0.63

**Fig. 4:**
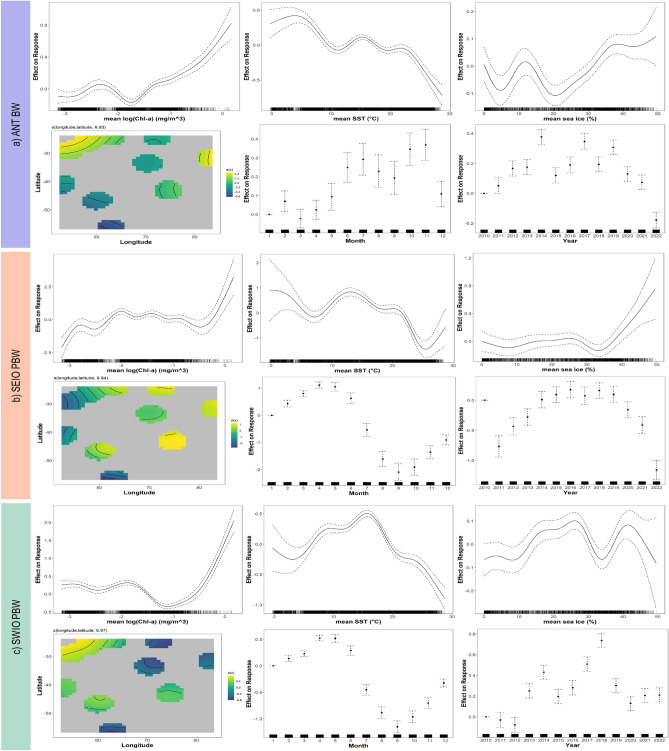
Functional relationships obtained with generalized additive models between DPM and environmental variables for the three acoustic populations: (**a**) ANT BW (explained deviance = 21.2%), (**b**) SEIO PBW (explained deviance = 57%), and c) SWIO PBW (explained deviance = 37.6%). Solid lines represent the estimated smooth functions and the dashed lines represent the approximate 95% confidence intervals. The x-axis corresponds to the linear predictor and the y-axis to the covariate. Tick marks on the x-axis show the distribution of data points.

**Fig. 5:**
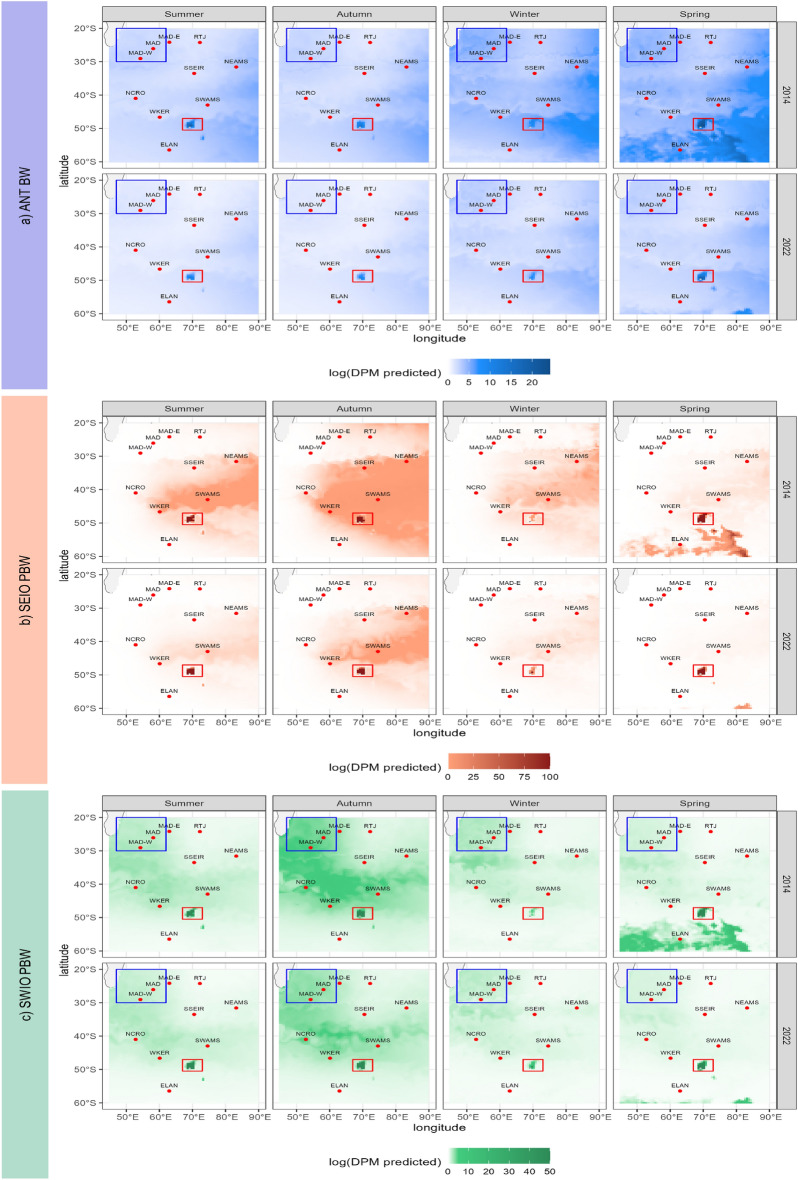
Predicted log(DPM) distribution for the three acoustic populations: (**a**) ANT BW, (**b**) SEIO PBW, and (**c**) SWIO PBW for the four seasons, represented by January (summer), April (autumn), July (winter), and November (spring), for the years 2014 and 2022. The red square indicates the Kerguelen Island region, and the blue square corresponds to the area east of Madagascar. The color scale is not the same for all acoustic populations. Predictions for each month and year are provided in supplementary materials.

**Fig. 6:**
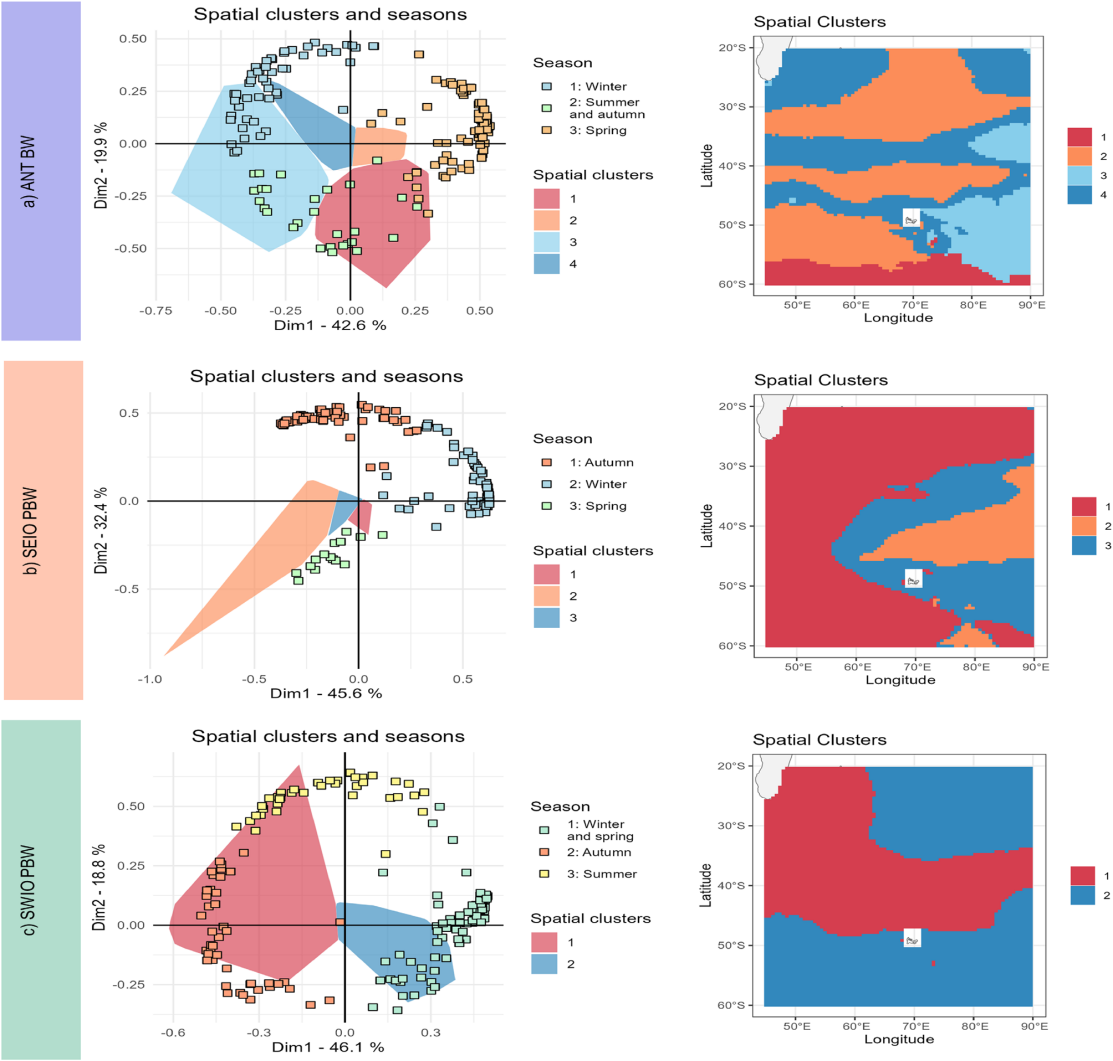
Functional areas and associated seasons for the three acoustic populations: (**a**) ANT BW, (**b**) SEIO PBW, and (**c**) SWIO PBW. (Left column) Projection of the standardized temporal loadings (seasons) and the standardized spatial factors (spatial clusters) onto the first two dimensions of the EOF. (Right column) Mapping of spatial clusters using standardized spatial factors to identify functional areas. The same colors are used to represent spatial clusters on the maps (right column) and on the first two dimensions of the EOF (polygons in the left column). In the left column, each season is represented by a square with a specific color. If the loadings of a season are in the same direction as a spatial cluster, then they are correlated and correspond to a couple season x functional area.

Each mooring consisted of an anchor, an acoustic release, an adjustable line, and a submerged buoy that houses the recording system. The instruments were deployed at depths ranging from 1000 to 1300 m on the axis of the Sound Fixing and Ranging (SOFAR) channel, which facilitates the transmission of low-frequency sounds over long distances^37^. The recording was continuous at 240 Hz sampling with a 24-bit analog-to-digital conversion. The hydrophones used had a sensitivity of -163.5 dB re 1V/μPa, except for the moorings at SWAMS in 2015 and 2016, where the sensitivity was -168.6 dB re 1V/μPa. The data collected were stored locally on each device^38^. The moorings were maintained during the annual scientific cruise of the R/V Marion Dufresne in the French Southern and Antarctic Territories in January/February each year. The database used in this study contains almost continuous acoustic recordings over 13 years, from 2010 to 2022.

### Acoustic data analysis

In this study, blue whale song detections from 2010 to 2018 were taken from Torterotot et al.^7^, and from 2019 to 2022 were processed using the same detection method, as follows.

A detection algorithm using dictionary learning and sparse representation was applied to automatically detect blue whale songs^39^. This technique uses large dictionaries to capture song variability and uses linear combinations of dictionary entries to represent song complexity. Without relying on a fixed template, the algorithm builds dictionaries directly from temporal call signals extracted from the data and reconstructs observed signals with sparse combinations of waveform entries. Higher-quality reconstructions correspond to higher similarity scores^39,40^. The signal used for recognition was the entire call for the ANT BW and a harmonic of a single unit of the call for both pygmy blue whales (see the white frames in Fig. 2). These harmonics were chosen for their high amplitude and frequency range that did not overlap with other blue whale songs.

The performance of this detection algorithm was evaluated on a substantial subset of manually annotated data spanning different seasons and locations within the OHA-SIS-BIO array. By setting the detection threshold to allow for one false positive per hour, we achieved a 90% recall for all calls with an SNR greater than 0 dB. A detailed review of all detections at the WKER location throughout the 2015 recordings confirmed that the number of false positives was very low, most of them caused by calls masked within the chorus^41^. The year-to-year frequency decrease in blue whale calls is very small (from -0.12 to .0.32 Hz/yr depending on the species)^15^. As a result, the dictionary from one year can still be applied effectively to the adjacent year. Although some calls may be missed due to a slight drop in frequency, most will still be detected successfully. A new dictionary was then built for each year of data, based on the calls detected by the adjacent year’s detector. This approach effectively accounts for the frequency shift in the calls. A detailed evaluation of the algorithm performance in the dataset used is provided by Torterotot et al. (2019)^41^.

### Environmental variables

In this study, three dynamic environmental variables were analyzed to explain the acoustic presence of blue whales derived from acoustic detections. These variables were chosen because they effectively characterize the environment in the study area, as discussed in the “Area description” section.

- Sea Surface Temperature (SST) Changes in sea surface temperature can signal shifts in regional biological and chemical processes that affect productivity. The SST data in this study come from the Copernicus Marine global ocean reanalysis (DOI: 10.48670/moi-00021), which covers the period from 1992 to 2024, with a high spatial resolution of 1/12$^{\circ }$ (approximately 8 km). The reanalysis includes observations from satellites such as TOPEX POSEIDON and ERS-1 and models of 3D thermodynamic and dynamic variables. SST is measured in degrees Celsius ($^{\circ }\hbox {C}$) with daily temporal resolution. Daily data were extracted and averaged over a 50 km area around each hydrophone, assuming the songs originated in that region. In addition, these averages were calculated weekly to align with the response variable under study. Figure 3 summarizes the SST data for the study area for each season.
- Chlorophyll-a concentration Chlorophyll-a concentration, a common indicator of primary productivity, is considered as a distal rather than proximal variable for prey such as zooplankton^42^. Although proximal variables typically better explain species distributions, the environmental variables chosen reflect prey distribution patterns^42^. Chlorophyll-a data were obtained from Mercator-Ocean, from January 1, 1993, to February 29, 2024, with a resolution of 1/4$^{\circ }$ and concentrations reported in mg/m$^{3}$ (DOI: 10.48670/moi-00019). Data were collected daily and averaged over a 50 km area per site to account for spatial variability. An overview of these data for the study area is presented in Fig. 3, which summarizes the chlorophyll-a data for each season.
- Sea Ice Concentration Sea ice significantly affects marine habitats, the availability of whale prey, and the migration of whales^28,43^. The sea ice concentration data come from the NOAA/NSIDC Climate Data Record, Version 4^44^, based on passive microwave measurements following NOAA criteria. It combines estimates from NASA Team and Bootstrap algorithms, using the highest precision^45^. Data are available at 25 km x 25 km resolution, daily and monthly. We analyze sea ice in a specific area (50$^{\circ }$E to 85$^{\circ }$E, -70$^{\circ }$S to -10$^{\circ }$S) by calculating pixels that exceed a concentration of 15% ice, a standard threshold to distinguish sea ice from open water^32^. An overview of these data for the study area is presented in Fig. 3, which summarizes the sea ice concentration data for each season.
- Geographic coordinates Geographic coordinates of the study sites were included in the models to capture spatial variability not captured by the other covariates and to account for large-scale distributions resulting from the geographical extent of the subspecies^5^.
- Temporal Parameters: Months and years Months and years were used as temporal predictors in the models to account for seasonal and annual variations in the acoustic presence of an acoustic population. These variations reflect the migratory patterns of the species and the inter-annual variability^7^.

In this study, the austral seasons are defined as follows: summer includes December, January and February; autumn includes March, April, and May; winter includes June, July, and August; and spring includes September, October, and November. To simplify the visual representation of the seasonal patterns in Figs. 3 and 5, a single representative month was selected for each season. Specifically, January was designated for summer, April for autumn, July for winter, and November for spring.

### Habitat modelling

The environmental variables (described above) may not have a linear relationship with the spatio-temporal distribution of the whales. Consequently, we modeled the acoustic presence of the whales with a spatio-temporal GAM^46^. This approach accounts for non-linear relationships with covariates, can deal with zero inflation through non-standard observation models (e.g. tweedie distribution), and models spatio-temporal correlations through spatial and temporal splines^47,48^.

To fit the model, we used observations from the average number of minutes with positive detections per day (DPM), averaged weekly. Observations were zero-inflated positive with heavy tails. Consequently, we tested two different likelihood distributions that can be considered for count data that exhibit overdispersion and excess zeros (Tweedie or Negative Binomial, Table 1) to model the observations. The averaged number of minutes with positive DPM $\mu _i$ is modeled through the equation:

$$g(\mu _i) = \mu _0 + \beta _{\text {Month}} \cdot \text {Month}_i + \beta _{\text {Year}} \cdot \text {Year}_i + f_1(\text {Chla}_i) + f_2(\text {SST}_i) + f_3(\text {MSI}_i) + f_4(\text {lon}_i,\text {lat}_i)$$

where *g* is a link function (here $\log$), $\mu _0$ is the intercept, the linear terms related to months and years are $\beta _{\text {Month}}$ and $\beta _{\text {Year}}$, $f_j, j\in \{1,\cdots ,4\}$ are the smoothed functions related to the covariates chlorophyll-a (Chla), sea surface temperature (SST), mean sea ice (MSI), and the spatial effect related to longitude (lon) and latitude (lat). Collinearity among environmental covariates (Chla, SST and MSI) was assessed using pairwise correlation coefficients. A strong negative correlation was found between Chla and SST (r = -0.81), but both variables were retained in the model due to their ecological relevance and because AIC-based model selection supported their inclusion.

From this model, we provide spatio-temporal maps of the acoustic presence of the whales by predicting $\hat{\mu }$ over a 50 km$^{2}$ spatial grid that covers the spatial domain at each time step. Different models were tested to investigate the spatio-temporal variations in the acoustic data (Table 1). In the M2 reference model, we modeled acoustic data as a function of months, years, environmental covariates, and spatial effects related to longitude and latitude. Two models of lower complexity were also considered (M0, M1, Table 1). We used Akaike’s information criterion (AIC) for model selection as a measure of the goodness of fit penalized by the number of parameters to account for model parsimony^49^. To avoid overfitting, we split the data into 80% training and 20% validation sets, and cross-validation was performed using Spearman’s rank correlation ($R_{\text {Spearman}}$) to assess model performance. The results showed improved performance for more complex models that incorporated temporal and spatial effects. Furthermore, the contribution of each covariate to the explained variance in the GAM was assessed by comparing the deviation of the full model with reduced models, where one covariate was removed at a time. This approach enabled calculation of the proportion of variance explained by each covariate as the relative change in deviation, normalized by the null deviation. The asterisks (*) indicate significant contributions (p < 0.001). These significance values are provided to indicate the importance of each covariate in explaining the variance in the model (Fig. Supplementary 4). Finally, the coefficients of variation of the model predictions were calculated as the standard deviation divided by the mean of the predictions (Fig. Supplementary 5). The model was implemented using the ’mgcv’ package^50^ in R^51^.

### EOFs to identify functional areas

To identify functional areas based on GAM spatio-temporal predictions, we relied on standard spatio-temporal dimension reduction methods known as Empirical Orthogonal Functions (EOFs)^52,53^.

EOFs are derived from climate science to extract the main spatial and temporal patterns in spatio-temporal data. They are principal component analysis (PCA) in a matrix where locations are individuals and time steps are variables. They have been widely used in marine ecology to analyze ecosystem variability, species distribution, and phenology^53,54^. Here, the spatio-temporal data are the model predictions $\mu$ at each location of the spatial grid for each time step (month of each year). They are stacked in a matrix $n \times m$ where *n* is the number of spatial locations (here X) and *m* is the number of time steps (here Y).

EOFs summarize information from a sequence of maps into a smaller set of *r* maps, denoted $\textbf{p}_j = (p_j(x_1),\cdots ,p_j(x_n))$ where *n* is the number of spatial locations. These terms (called spatial factors) best represent the spatio-temporal variability patterns of the spatio-temporal variable. The first spatial factor of the EOF $\textbf{p}_1$ captures the highest amount of variability in the spatio-temporal data; the second $\textbf{p}_2$ is orthogonal to the first spatial factor $\textbf{p}_1$ and captures the second highest amount of variability. The same principle applies to the next dimensions.

For each dimension *j*, the spatial factor is related to a set of temporal indices called (temporal) loadings defined as $\varvec{\alpha }_j = (\alpha _j(t_1),\cdots ,\alpha _j(t_m))$, where *m* is the number of time steps. If the loading of a time step is positive ($\alpha _j(t) > 0$), then the spatio-temporal variable is distributed according to the related spatial factor $\textbf{p}_j$. If the loading of a time step is negative ($\mathbf \alpha _j(t) < 0$), the spatio-temporal variable is distributed according to the opposite of the related spatial factor $-\textbf{p}_j$. More details on the interpretation of EOFs can be found in Alglave et al. (2024)^55^.

As with standard PCA, EOFs can be combined with clustering analysis (here Hierarchical Clustering on Principal Components) to regroup locations that have similar temporal trends (clustering on spatial factors) or time steps that have similar spatial patterns (clustering on temporal loadings)^53^. Clusters of locations can be interpreted as distinct functional habitats, while clusters of time steps can be interpreted as ecological seasons. Both can be plotted on a single graph, and locations can be related to seasons if they are in the same direction on the graph (similar to the case in PCA).

Note that both EOF dimensions and clustering can be strongly affected by extreme values. Given the high predictions surrounding Kerguelen Island, which distort the EOF representation of broad-scale spatial patterns, we exclusively retained predictions outside the Kerguelen area to perform the EOFs and clustering for the SEIO PBW and SWIO PBW acoustic population. EOF analysis and clustering were carried out using the ‘FactoMineR’ library^56^ in R^51^.

## Results

### Acoustic effort

Acoustic effort and detection data for each site and acoustic population are summarized in Table Supplementary 1. The total recording effort exhibited variation between sites, ranging from 1,630 days (NCRO) to 4,750 days (WKER), with a range of recording durations between sites, totaling approximately 2.3 million minutes (NCRO) to 6.8 million minutes (WKER). The proportion of days with acoustic presence exhibited variation across sites and acoustic populations, ranging from 0.0% at MADW for the SEIO PBW acoustic population up to 95.6% at NEAMS for the ANT BW acoustic population. Acoustic effort data reflect continuous and intermittent recording throughout the study period, with the frequency of detections varying over time. Although some sites show nearly constant acoustic presence (e.g., MAD, NEAMS), others exhibit more intermittent detections (e.g., RTJ, ELAN). The acoustic presence of each acoustic population and the recording periods are detailed in the supplementary materials (Fig. Supplementary 1, Supplementary 2, Supplementary 3).

### Spatio-temporal variations in environmental covariates

The thermal gradient in the study area (Fig. 3) ranges from -4$^{\circ }$C to more than 25$^{\circ }$C, exhibiting a discernible latitudinal pattern, particularly evident during austral summer and autumn, with a SST peak in the northwest. These temperatures remain consistent from year to year and demonstrate minimal variability (Fig. 3a). Conversely, chlorophyll-a concentration exhibits an inverse latitudinal gradient with concentrations ranging from 7.38 mg/m$^{3}$ in nutrient-rich zones to 0.04 mg/m$^{3}$ in nutrient-poor zones, reaching its peak near the Kerguelen Plateau in austral summer. In 2014, inter-annual variability was observed, with a notable peak in primary productivity in austral spring, compared to the other years (Fig. 3b). The sea ice cover (Fig. 3 c) shows stable seasonal patterns, with slight variability, peaks in austral winter, with ice cover ranging from 0% to almost 50% of the region.

### Model selection

Table 1 delineates the hypotheses, model configurations, and AIC values pertinent to each acoustic population: ANT BW, SEIO PBW and SWIO PBW. For all species examined, model M2, characterized by its incorporation of spatial, monthly and annual effects in conjunction with all environmental covariates such as chlorophyll-a concentration, sea surface temperature, and sea ice cover, exhibited the lowest AIC and the highest explanatory power regarding deviation, although anticipated. Among the two tested distributions, the Tweedie distribution demonstrated superior performance in the context of model M2. In addition to AIC-based model selection, cross-validation using Spearman’s rank correlation ($R_{\text {Spearman}}$) further supported the superiority of model M2, revealing improved predictive performance for more complex models incorporating temporal and spatial effects. Ultimately, the chosen model represented 21. 2% of the deviation for ANT BW, 57% for SEIO PBW, and 37.6% for SWIO PBW, and was validated through both AIC and cross-validation metrics.

### Monthly habitat models and predicted distributions

#### ANT BW

ANT BW songs were detected at all recording stations, demonstrating a large representation within the study area (Fig. Supplementary 1). Detection rates are significantly higher at the NEAMS site and comparatively lower at the SSEIR and RTJ sites (Fig. Supplementary 1). The covariates that contribute the most significantly to the variance in our model include sea surface temperature, chlorophyll-a concentration, sea ice extent, GPS coordinates, temporal year parameters, and month. These covariates were identified as statistically significant (p < 0.001)) (Fig. Supplementary 4). There is a stable positive correlation between chlorophyll-a concentration and acoustic presence, except at very high chlorophyll-a concentrations, where further increases in acoustic presence are observed (Fig. 4a). In particular, this correlation starts with a logarithmic value of log(mean Chla) = -1.8 mg/m$^{3}$ (Fig. 4a). Seasonal peaks in acoustic events were notably discernible, particularly during austral winter and spring (Fig. 4a). In contrast, there is a negative correlation between temperature and acoustic presence, markedly above 20$^{\circ }$C, with a plateau and even a second peak in the range of 10 to 20$^{\circ }$C (Fig. 4a). The relationship between sea ice extent and acoustic presence reveals variability at lower percentages of sea ice coverage, with an increase in daily positive minutes (DPM) concurrent with initial ice formation, followed by a secondary positive peak at higher percentages of ice coverage (Fig. 4a). The annual variability in the acoustic presence exhibited significant peaks in the years 2014, 2017, and 2019, with predicted contributions of approximately 0.3, 0.35, and 0.4, respectively, followed by an observable downward trend post-2019 (Fig. 4a). The inter-annual variability in the predicted contributions over the years ranged from around 0.1 in 2010 to a maximum of 0.4 in 2019, subsequently decreasing to approximately 0.2 in 2022.

The predicted areas of acoustic presence extend throughout the study region, with a higher acoustic presence predicted around Kerguelen Island and its plateau, and adjacent to the offshore areas of Madagascar (Fig. 5a). The intra-annual variability in acoustic presence is evident in all four seasons. The highest acoustic presence is observed during austral winter and spring, with predicted contributions ranging between 0.3 and 0.4. In contrast, summer and autumn show significantly lower contributions, ranging between 0 and 0.1 (Fig. 4a). Despite these seasonal fluctuations, the Kerguelen area remains consistently occupied throughout the year, with vocalizations detected in all seasons (Fig. 4a). All predicted DPM maps are available in the supplementary material, from Fig. Supplementary 8 to Fig. Supplementary 20.

#### SEIO PBW

SEIO PBW are mainly detected acoustically at locations located east of the observation network, specifically at SWAMS and NEAMS, whereas they are nearly absent from northwestern sites such as MAD and southern locations such as ELAN (Fig. Supplementary 2). The covariates that account for the most significant variance in the model include chlorophyll-a concentration, sea surface temperature, sea ice coverage, GPS coordinates, month, and year. All of these covariates are statistically significant (p < 0.001) (Fig. Supplementary 4).

Chlorophyll-a concentration exhibits a notable relationship with acoustic presence. Very low chlorophyll-a concentrations (log (mean Chla) = -3 mg/m$^{3}$) are associated with a very low acoustic presence, while higher chlorophyll-a concentrations (log (mean Chla) = -0.4 mg/m$^{3}$) are associated with a stronger acoustic presence. Between these values, the relationship remains stable (Fig. 4b). Regarding sea surface temperature, a negative relationship with acoustic presence is observed at low temperatures, specifically 0 to 5$^{\circ }$C, followed by a rebound and stabilization between 10 and 20$^{\circ }$C, and a return to a negative relationship at temperatures exceeding 20$^{\circ }$C (Fig. 4b). There is no significant correlation with the percentage of sea ice coverage, except in higher percentages (Fig. 4b). GPS coordinates contribute significantly to spatial variability, with a higher acoustic presence observed in the eastern part of the study area, particularly around the Kerguelen Islands (Fig. 5b).

In terms of temporal variability, there is marked seasonal variation in the acoustic presence. Activity peaks during the austral summer and extends into the autumn (between 0 and 1). The acoustic presence decreases significantly during the winter months, with values dropping to near -2 and remaining low during early spring (-1; Fig. 4b). The inter-annual variability in acoustic presence is evident, with a steady increase observed until 2019 (from approximately -0.7 to 0), followed by a subsequent decline to below -1 in 2022 (Fig. 4b). All predicted DPM maps are available in the supplementary material, from Fig. Supplementary 21 to Fig. Supplementary 33.

#### SWIO PBW

SWIO PBW are broadly present within the network, but are quasi-absent from extreme northeastern sites like RTJ and extreme southern sites like ELAN (Fig. Supplementary 3). The highest detection rates occur in the western part of the network, particularly at offshore sites near Madagascar, such as MAD, MADE, and MADW (Fig. Supplementary 3).

The covariates that have the most significant impact on the variance of our model include chlorophyll-a concentration, sea surface temperature, GPS coordinates, month, and year. All of these covariates are statistically significant (p < 0.001)) (Fig. Supplementary 4). Chlorophyll-a concentration shows a positive correlation with acoustic presence only at higher concentrations (log (mean Chla) -1.2 mg/m$^{3}$) (Fig. 4c). For SST, there is a generally negative relationship with acoustic presence at temperatures near 0$^{\circ }$C and a stronger negative correlation at temperatures exceeding approximately 15$^{\circ }$C (Fig. 4c). No significant correlation is observed with the increasing percentage of sea ice coverage (Fig. 4c). The predicted areas of acoustic presence are spread throughout the region, with notably high predictions off Madagascar and around Kerguelen, while the acoustic population is not predicted to be present south of 50$^{\circ }$S or in the northeast region (Fig. 5 c). Seasonally, the acoustic population exhibits strong peaks in activity during austral summer and autumn (between 0 and 0.5), with a notable peak in 2018 (approximately 0.8). Since 2019, the acoustic population has shown a more stable pattern (around 0.2) (Fig. 4c). All predicted DPM maps are available in the supplementary material, from Fig. Supplementary 34 to Fig. Supplementary 46.

### EOF to identify functional areas

For the three acoustic populations, the first two dimensions of the EOF were selected, which explain more than 60% of the variance for the ANT BW and SWIO PBW acoustic populations, and more than 75% for the SEIO PBW acoustic population. Additional dimensions were excluded from the analysis due to their tendency to represent noise (Fig. Supplementary 6). Each acoustic population exhibits strong spatio-seasonal patterns with succession between a summer/autumn season and a spring/winter season. The cluster analysis highlights the different functional areas predicted for whales during the different seasons for each species (Fig. Supplementary 7).

For the ANT BW subspecies, three temporal clusters and four spatial clusters were predicted (Fig. 6a). Spatial clusters 1 and 4 are predicted for summer and autumn, mainly in the Kerguelen Plateau and higher latitudes. Spatial cluster 2 is predicted for spring and shows limited acoustic activity, concentrated mainly in the southwestern region. Spatial cluster 3 is predicted for winter, predominantly near the coasts or islands within the area.

For SEIO PBW, two temporal clusters and three spatial clusters were predicted (Fig. 6b). Spatial clusters 1 and 2 are predicted for summer and autumn, mainly in the eastern areas surrounding the Kerguelen Plateau. Spatial cluster 3, predicted for winter and spring, suggests that this acoustic population is likely outside the study area.

For SWIO PBW, three temporal clusters and three spatial clusters were predicted (Fig. 6c). In winter, the acoustic population is expected to be mainly located in spatial cluster 3, off Madagascar. In spring, acoustic activity is predicted in clusters 1 and 3, either off Madagascar or beyond the zone. In summer and autumn, the acoustic presence is predicted in clusters 1 and 2, extending from off Madagascar to the Kerguelen Plateau.

## Discussion

Using generalized additive models (GAMs) and empirical orthogonal functions (EOFs), we identified key relationships between acoustic presence and environmental variables. ANT BW acoustic activity was strongly associated with low sea surface temperatures, high chlorophyll-a concentrations, and extensive sea ice cover, primarily during austral winter and spring. In contrast, the acoustic populations of SEIO and SWIO PBW showed peak activity in temperate waters (12-15 $^{\circ }$ C) with intermediate chlorophyll-a concentrations, revealing different ecological niches. Three critical habitats were identified in the southern Indian Ocean: (1) Madagascar, which serves as a key potential resting area during the austral winter; (2) the Kerguelen Plateau, an essential summer and fall habitat characterized by high primary productivity, which likely supports both feeding and resting activities; and (3) an overlap zone for the SEIO and SWIO PBW acoustic populations in summer, suggesting possible interactions such as hybridization or competition. Our approach combines the flexibility of GAMs with the dimensionality reduction power of EOFs. These methods, coupled with the extensive spatial and temporal scales of the dataset, provide novel insights into the migratory patterns, habitat use, and ecological drivers of blue whale distributions^57,58^.

A principal advantage of our approach is its ability to predict the acoustic presence of whales in unsampled regions, offering a comprehensive understanding of their distribution over vast marine areas. We used the number of positive minutes per day, averaged weekly, metric to effectively capture inter-annual and seasonal variations of blue whale acoustic presence over a dataset with extensive spatial and temporal coverage. The incorporation of EOFs further improved spatio-temporal dimensionality reduction, providing novel insights into critical habitats and seasonal dynamics of blue whale acoustic populations.

The results of our model align with the established knowledge on the ANT BW acoustic population. These cetaceans are predominantly detected during austral winter and spring, with peak acoustic activity correlated with low temperatures, high chlorophyll-a concentrations, and extensive sea ice cover. The spatial predictions of the model, reflecting the temporal variability, corroborate known migratory behaviors, where the ANT BW feed in the southern ocean during the summer and migrate to lower latitudes during the winter months^2,3,5,20^. Furthermore, inter-annual variability in detection patterns is evident, with a notable decline in acoustic presence after 2019. This fluctuation could be linked to changes in environmental conditions, such as variations in sea ice extent and primary productivity, or anthropogenic factors, suggesting dynamic habitat use and potential shifts in blue whale distribution over time.

These findings are consistent with previous research that emphasizes the importance of sea ice and the availability of prey in the distribution of ANT BW. The positive correlation between acoustic detections and sea ice extent supports the results of several studies^29,30^. Furthermore, the prominence of chlorophyll-a as a proxy of prey availability is consistent with observations in similarly productive areas such as the Northeast Pacific and Northern Patagonia^59,60^.

In particular, our results suggest that the eastern waters of Madagascar may serve as a significant resting area for ANT BW during austral winter. These regions, characterized by moderate temperatures and productivity, are mainly visited during this season, indicating their potential role as temporary refuges or resting areas. The Kerguelen Plateau appears to play a prominent role during the austral summer and autumn, where a significant acoustic presence of blue whales is observed. This area may function both as a resting zone before migration to higher latitudes to feed in summer and as a feeding site for non-migrating individuals^5,61^. Its substantial primary productivity, particularly in krill abundance^62^, supports this hypothesis, highlighting the importance of Kerguelen as a critical habitat for blue whale acoustic populations.

SEIO and SWIO PBW exhibit different spatial distribution patterns throughout the year. However, these acoustic populations were recorded simultaneously during the austral summer and autumn, with maximum activity observed in regions characterized by intermediate temperatures (12–15 °C) and high chlorophyll-a concentrations. This spatial and temporal overlap supports hypotheses from previous studies, which suggest potential interactions between these two acoustic populations, such as hybridization or competition for food resources^63^. EOF analysis further improves understanding of habitat use by identifying spatio-temporal patterns consistent with those described in other dynamic models^64,65^.

In contrast to ANT BW, pygmy blue whales show a minimal association with sea ice, which has limited influence on the predictions in our model. This observation is consistent with studies of SEIO PBW acoustic populations, which do not migrate as far south to feed. Satellite telemetry studies have shown that these whales are not detected south of 42$^{\circ }$S, which confirms their tendency to avoid regions with extensive sea ice cover^66,67^. The hypothesis of geographical separation within the SEIO PBW acoustic population is supported by recent studies documenting the presence of SEIO PBW in the Kerguelen Plateau and southern Australian waters during the same austral summer and autumn season^21,22^. Acoustic data from Kerguelen and southern Australia overlap seasonally, reinforcing the idea that the Kerguelen Plateau is an important feeding ground for these whales, especially given its high primary productivity. Satellite tracking further supports this, showing that SEIO PBW individuals migrate south from Indonesia to Kerguelen for feeding but also travel to southern Australia, indicating a potential division within the population into two distinct migratory routes^66,67^. These observations suggest that the SEIO PBW population may follow different migratory routes, with migrations to Kerguelen driven by foraging rather than reproduction, as these whales are known to reproduce in Indonesian waters. Although Kerguelen’s abundant krill^62^ makes it a critical food source, separation into distinct migratory routes and ecological zones may not be fixed.

Similarly, SWIO PBWs disperse as they migrate across the southwestern Indian Ocean. Their range extends from the south of Marion and Prince Edward Islands, where acoustic detections have been reported between December and April^68^ to the south of Amsterdam Island in the east, with detections occurring from December to July^7^. The region surrounding Prince Edward Island has been identified as a significant feeding ground during this period^68^. The concomitant recording of SWIO PBW songs near Kerguelen in our study posits the potential for Kerguelen to also function as a feeding site. This suggests that there may be a spatial dispersion within the SWIO PBW population during summer - mid-fall, with individuals following different migratory pathways while exploiting similar ecological resources. Northward migration begins in April, with individuals traveling toward Madagascar, where the migratory route splits: one branch passes through the Mozambique Channel^24^, while the other skirts offshore around Réunion and Mauritius^7^. The northernmost acoustic detections have been recorded near Mayotte between May and July^25^.However, all acoustic monitoring stations across the Indian Ocean report an absence of calls from August to October^6,7,24,68^, which may indicate a shift in vocal behavior or a seasonal movement of the population to a more northerly, equatorial region that remains unsurveyed. Migration to the central and eastern tropical Indian Ocean is unlikely, as no calls have been detected around the Seychelles or Diego Garcia^69,70^. During their return south (October to January), SWIO PBW calls are not detected by offshore acoustic stations in the western Indian Ocean^7^. However, they are consistently detected along the western and eastern coasts of Madagascar^6,24,25^, suggesting that coastal waters provide a more favorable habitat during this part of the migratory cycle.

Our results further advance current knowledge by emphasizing the role of the Kerguelen Plateau for pygmy blue whale acoustic populations as a shared habitat during austral summer and autumn. This area, with its high primary productivity and abundance of krills^62^, is likely to support feeding and resting activities for these acoustic populations. Identifying key habitats underscores the ecological importance of these areas for the conservation of blue whales. The region serves as a biodiversity hotspot, supporting not only blue whales but also other predators such as elephant seals and penguins^71,72^.

This study has certain limitations to consider. First, ambient noise, which can affect call detection, was not explicitly taken into account in our model. High levels of noise from natural or anthropogenic sources could mask whale calls and lead to underestimations of acoustic activity^59,64^. Including ambient noise as a covariate in future studies would improve the accuracy of the model. To address these limitations, we estimate a detection range of 50 km around the hydrophones to account for sound propagation and minimize bias. An estimated detection range of 50 km was determined as a conservative figure, reflecting the substantial variability in the detection ranges documented in the literature. These ranges exhibit substantial variation, ranging from 10 km to more than 700 km, depending on factors such as geographical region, seasonal variations, and source levels between acoustic populations^68,73,74^.

Secondly, this analysis intentionally excluded D-calls, which represent short acoustic signals typically associated with foraging activities and are believed to aid in prey detection or coordination during feeding^9^. These vocalizations were omitted because of their inability to be reliably attributed to a distinct acoustic population. However, the inclusion of D-calls in future research could provide deeper insights into the various behavioral contexts of blue whale vocalization, especially in highly productive areas such as the Kerguelen Plateau. This proposition is corroborated by Torterotot et al. (2023)^75^, who reported high occurrence of D-calls near the Kerguelen Plateau. In contrast, the blue whale songs examined in this study are predominantly associated with reproductive activities, similar to those observed in humpback whales^13,76^. However, their presence in feeding regions^77,78^ implies that they may perform additional functions.

Third, we used chlorophyll-a concentration as a proxy for prey availability, which may not directly reflect krill biomass, the primary food source for blue whales. This limitation stems from the difficulty of obtaining direct measurements of the biomass of krill in such vast regions and time frames. Future work should incorporate direct measurements of krill biomass, such as acoustic surveys or satellite data, to better capture prey dynamics and refine habitat models.

In addition, we focus on broad-scale variables such as SST, chlorophyll-a, and sea ice extent. Although this approach is ecologically relevant on large scales, finer-scale factors such as nutrient upwelling or mesoscale eddies could improve local habitat models and provide deeper ecological insights.

In conclusion, this study sheds light on the complex relationships between the acoustic populations of blue whales and their environment. By identifying functional zones shaped by environmental factors, it establishes a foundation for targeted conservation efforts to preserve these critical marine ecosystems.

## Supplementary Information

Supplementary Information.

## Data Availability

The environmental data used in this study are publicly available from dedicated databases: Sea Surface Temperature (SST): Data were obtained from the Copernicus Marine global ocean reanalysis (DOI: 10.48670/moi-00021), covering the period 1992 to 2024 with a spatial resolution of 1/12$^{\circ }$ and daily temporal resolution. Chlorophyll-a concentration: Data were sourced from Mercator-Ocean (DOI: 10.48670/moi-00019), available from January 1993 to February 2024 at a 1/4$^{\circ }$ resolution. Sea Ice Concentration: Data were obtained from the NOAA/NSIDC Climate Data Record, Version 4 (DOI: 10.7265/efmz-2t65), based on passive microwave measurements and available at a 25 km × 25 km resolution with daily and monthly temporal scales. These environmental data were used to generate Fig. 3 in the manuscript and were included as explanatory variables in the models used throughout the study. The acoustic data used in this study (DOI: 10.18142/229) were used to generate Fig. 2 and served as the response variable in our model. The raw acoustic data is not publicly available, but the detection data can be obtained from Jean-Yves Royer (jean-yves.royer@univ-brest.fr) upon reasonable request. Additional data sets were not generated or analyzed beyond those specified.
