# Supplementary figures and images for: Modelling the influence of environmental factors on the acoustic presence of blue whale populations in the southern Indian Ocean
Source: Sci Rep. 2025 Jul 2;15:23218. doi: 10.1038/s41598-025-02941-9 (PMC12223265; doi:10.1038/s41598-025-02941-9)

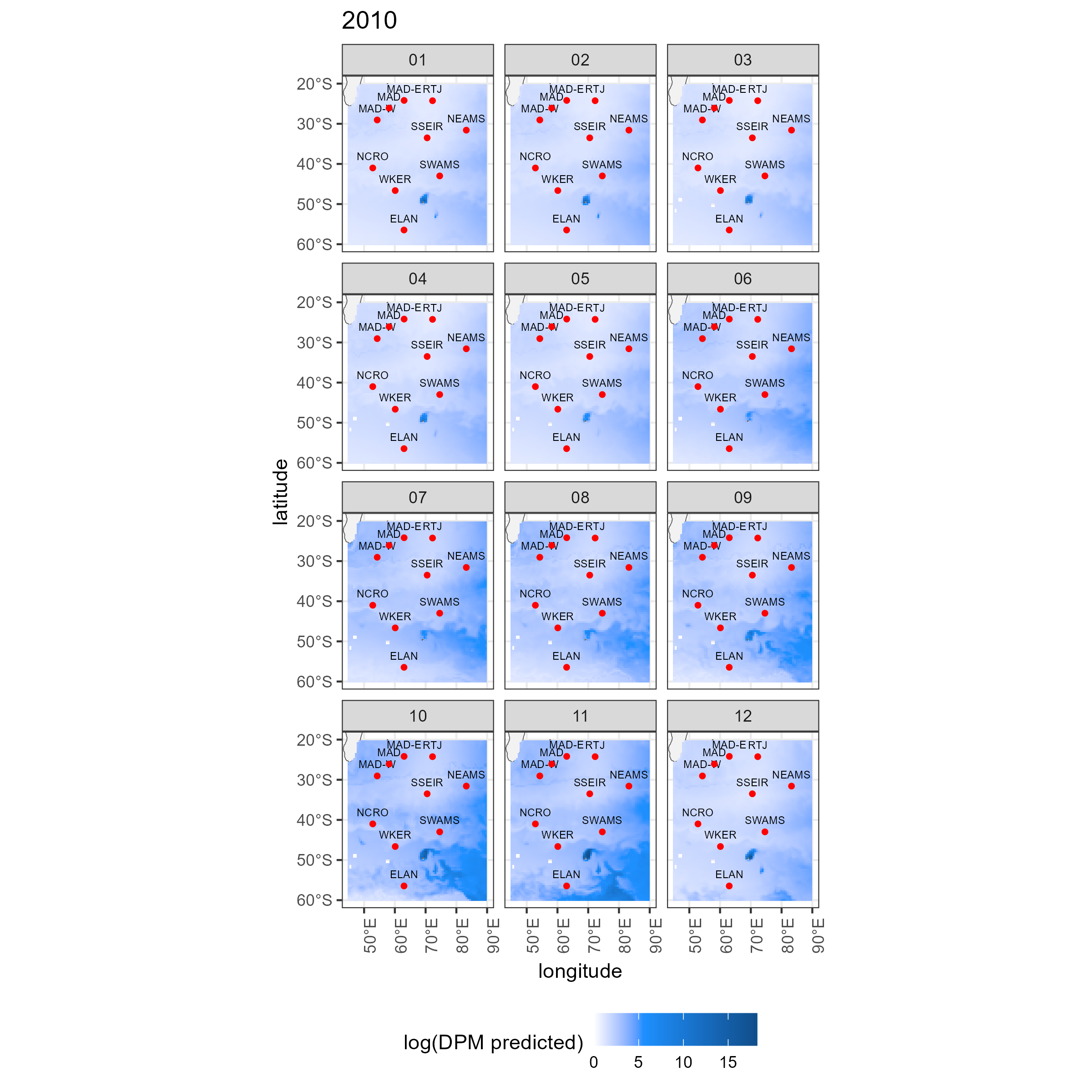

Supplement: Supplementary file 1 — Supplementary Information. [file 41598_2025_2941_MOESM1_ESM.zip › supp fig/antbw/plot_prediction_antbw_2010.png]

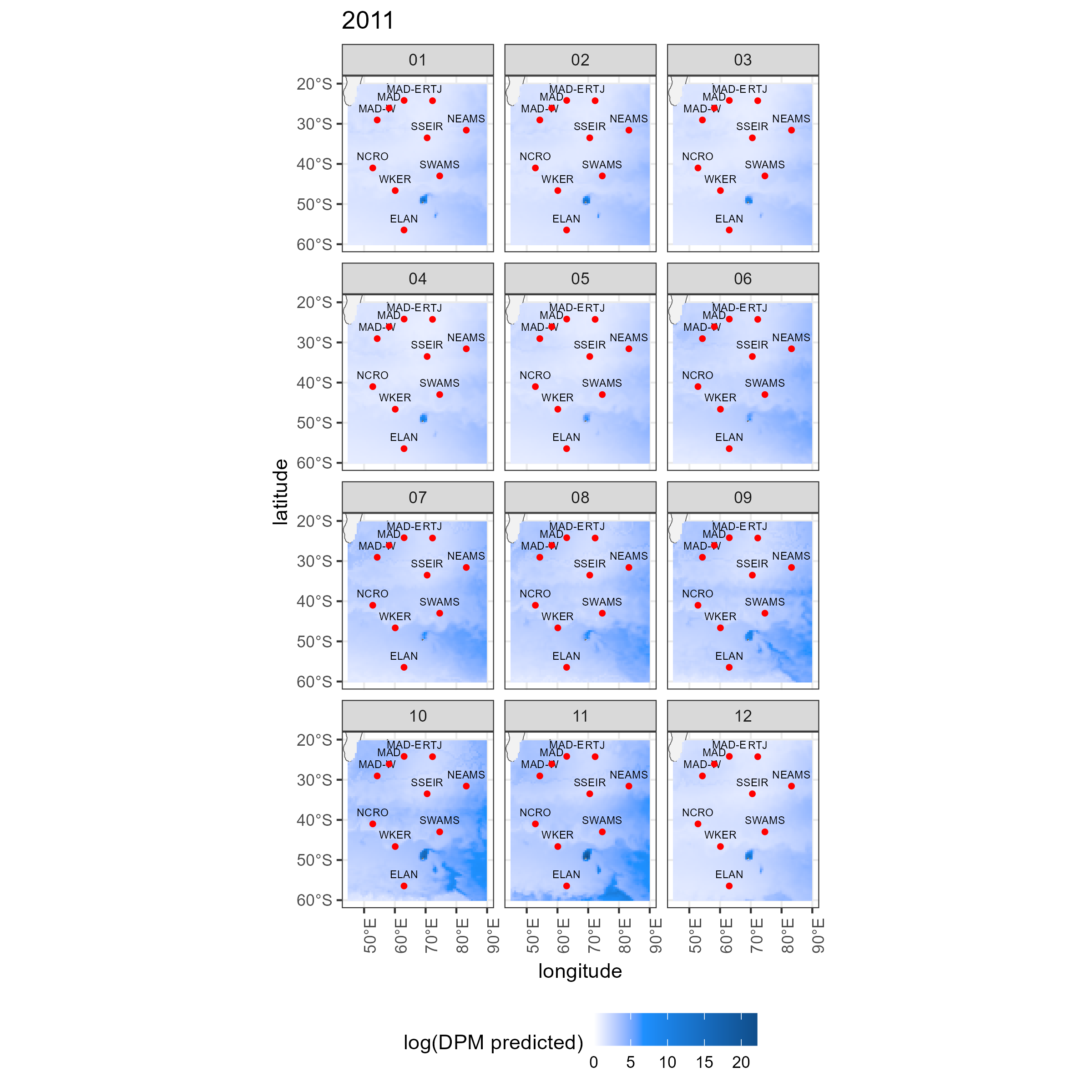

Supplement: Supplementary file 1 — Supplementary Information. [file 41598_2025_2941_MOESM1_ESM.zip › supp fig/antbw/plot_prediction_antbw_2011.png]

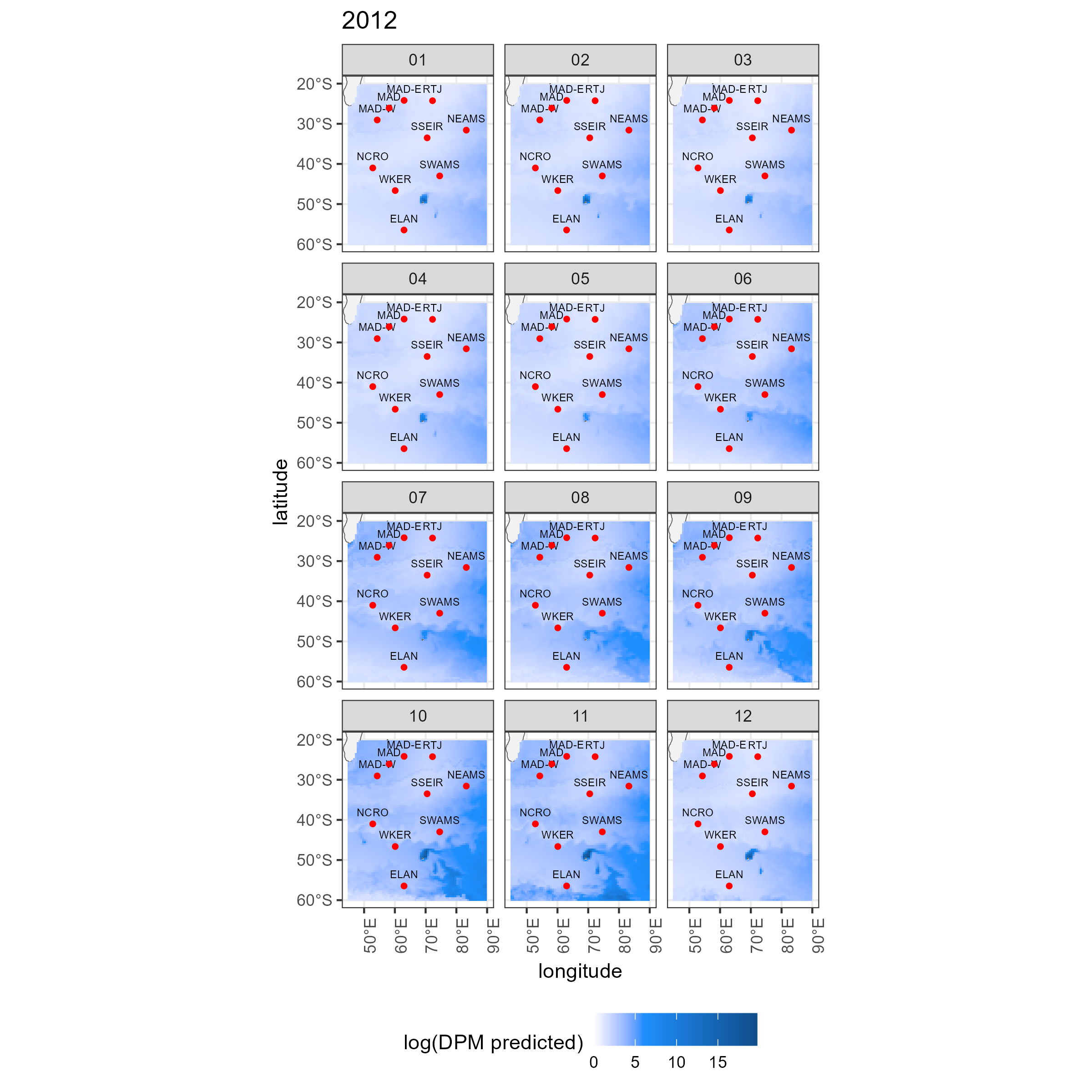

Supplement: Supplementary file 1 — Supplementary Information. [file 41598_2025_2941_MOESM1_ESM.zip › supp fig/antbw/plot_prediction_antbw_2012.png]

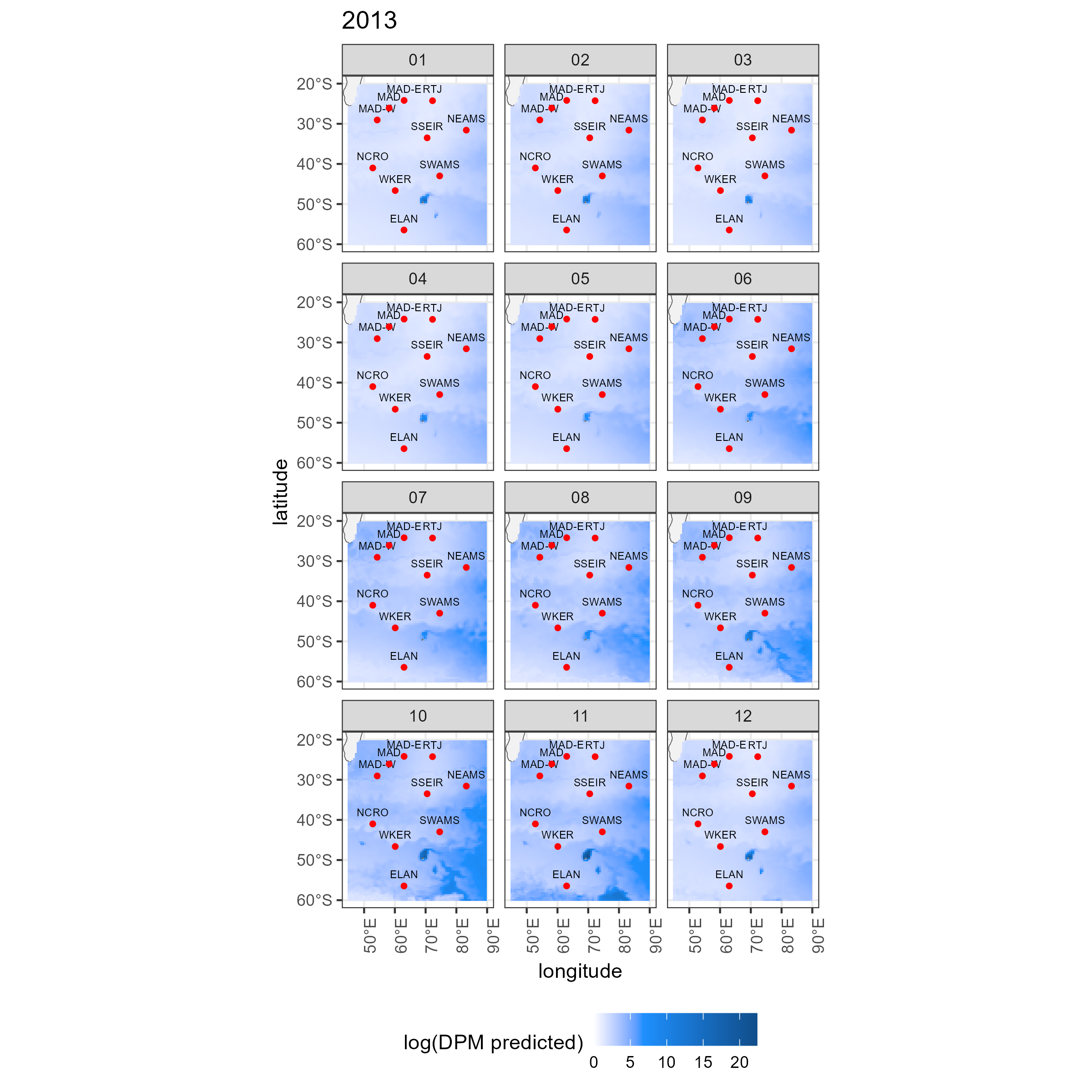

Supplement: Supplementary file 1 — Supplementary Information. [file 41598_2025_2941_MOESM1_ESM.zip › supp fig/antbw/plot_prediction_antbw_2013.png]

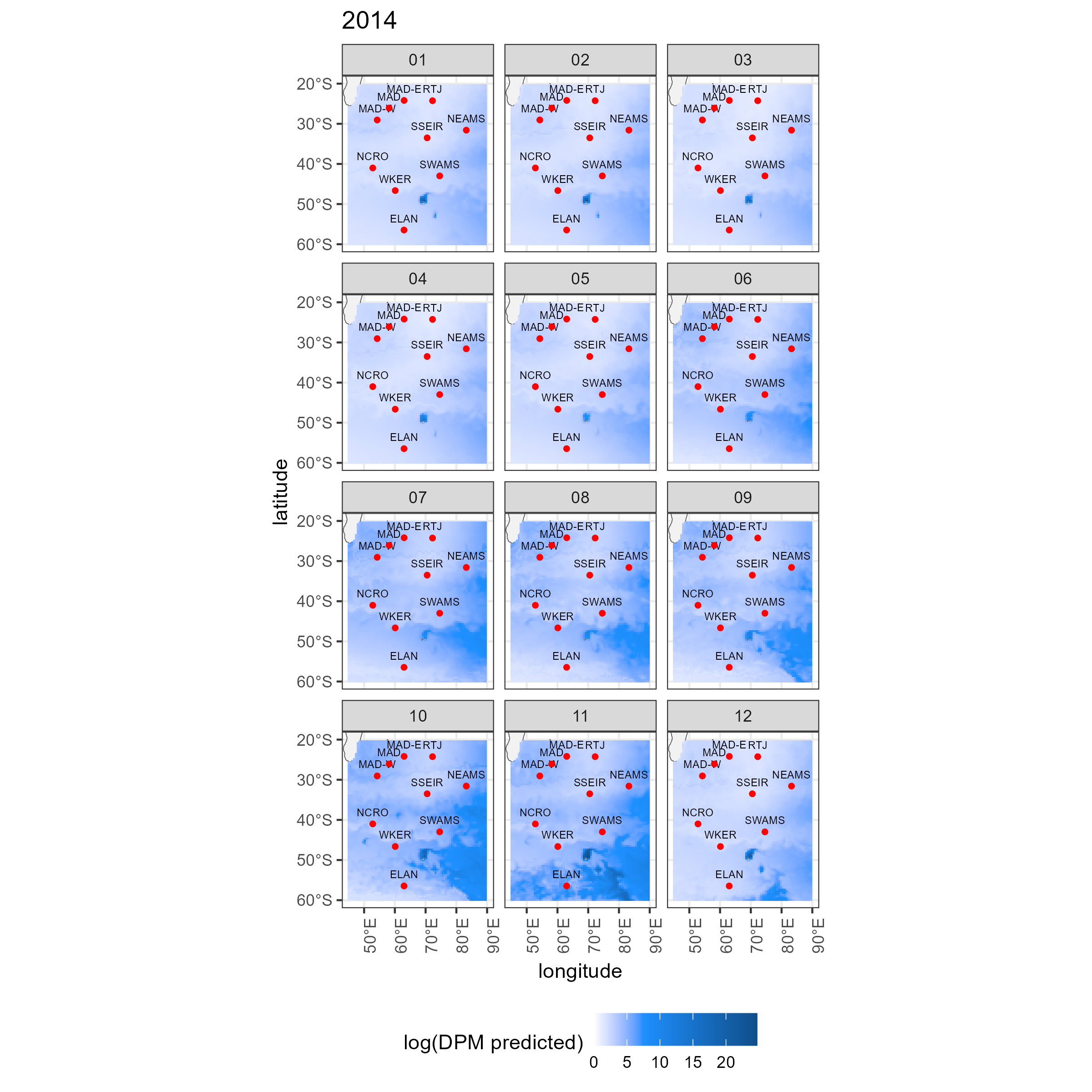

Supplement: Supplementary file 1 — Supplementary Information. [file 41598_2025_2941_MOESM1_ESM.zip › supp fig/antbw/plot_prediction_antbw_2014.png]

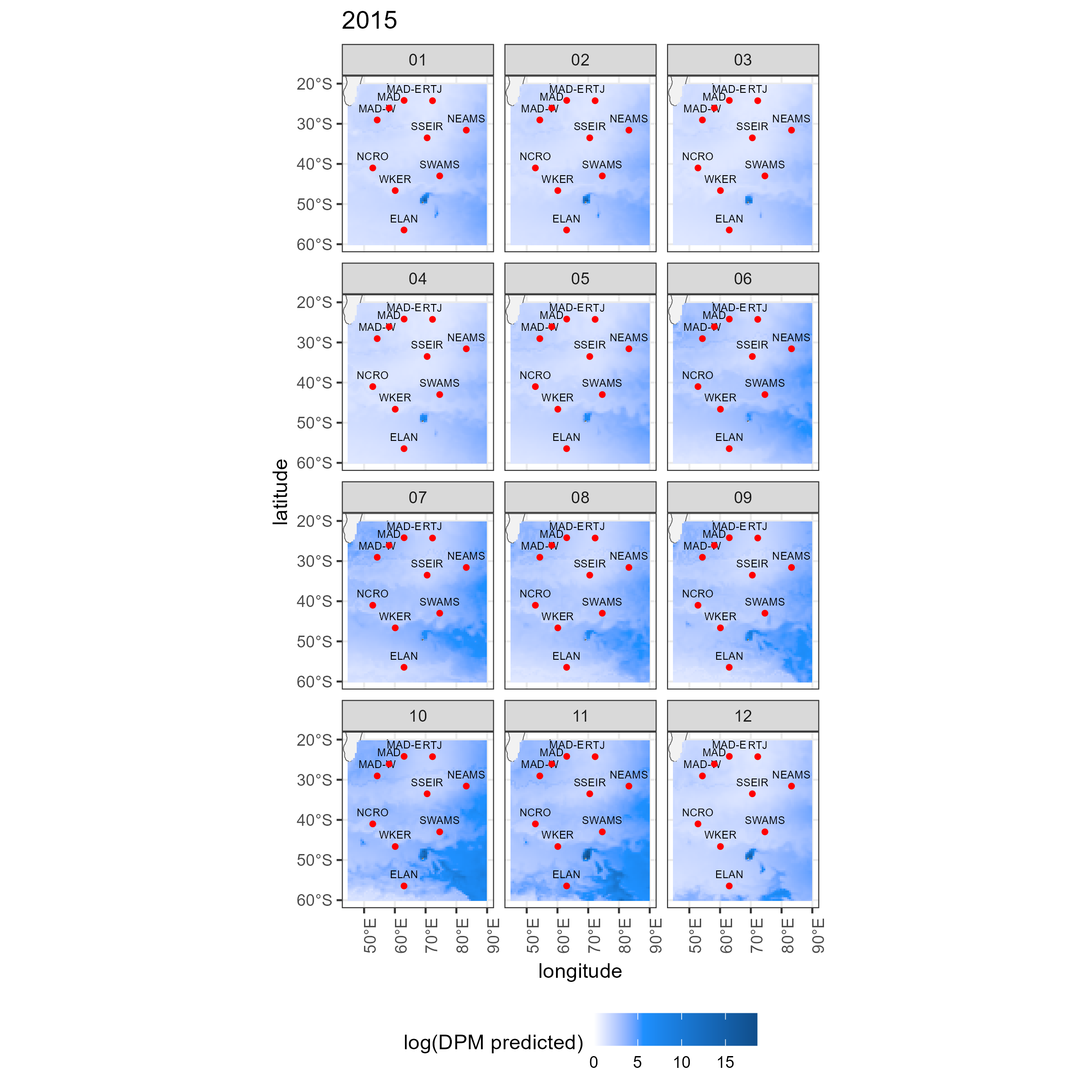

Supplement: Supplementary file 1 — Supplementary Information. [file 41598_2025_2941_MOESM1_ESM.zip › supp fig/antbw/plot_prediction_antbw_2015.png]

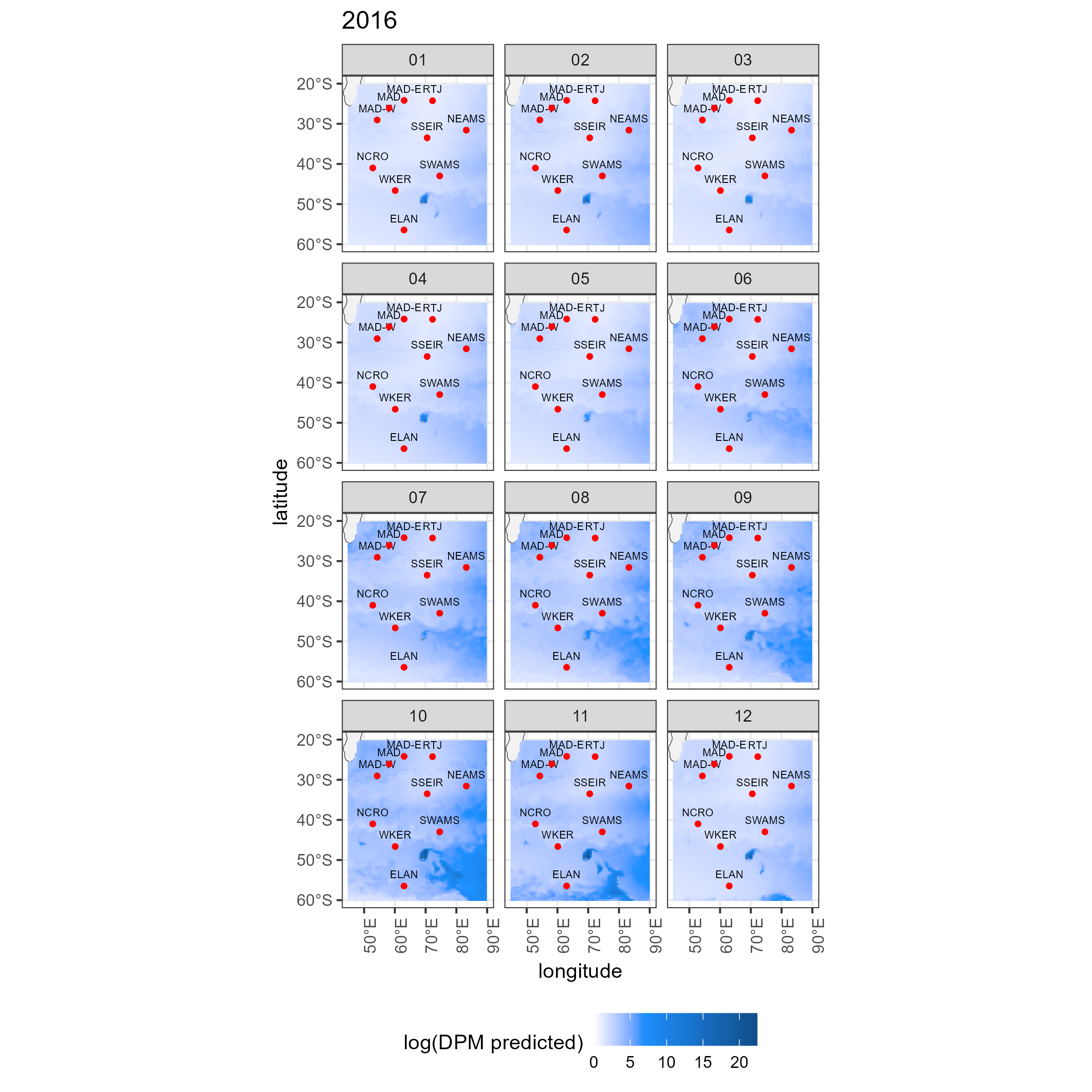

Supplement: Supplementary file 1 — Supplementary Information. [file 41598_2025_2941_MOESM1_ESM.zip › supp fig/antbw/plot_prediction_antbw_2016.png]

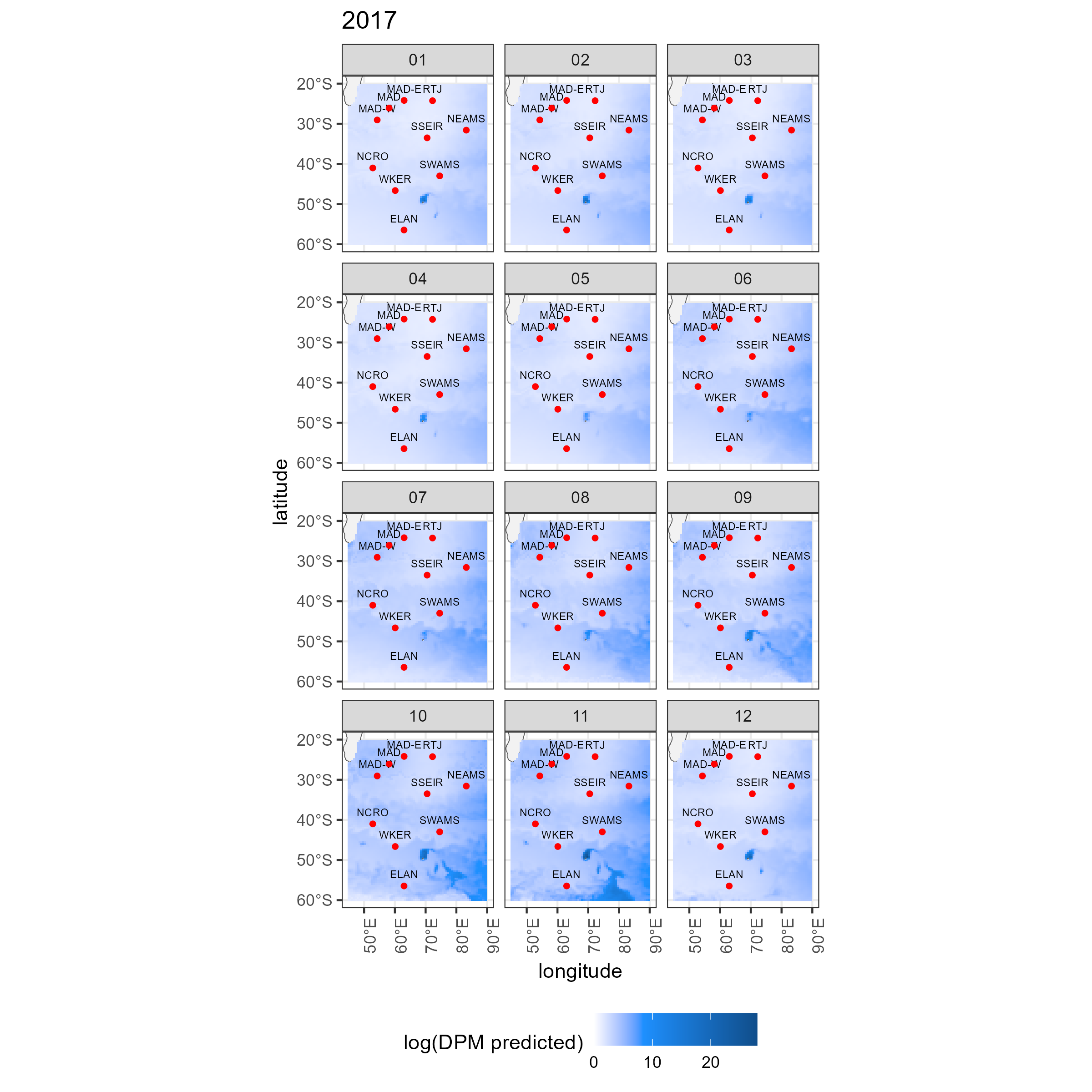

Supplement: Supplementary file 1 — Supplementary Information. [file 41598_2025_2941_MOESM1_ESM.zip › supp fig/antbw/plot_prediction_antbw_2017.png]

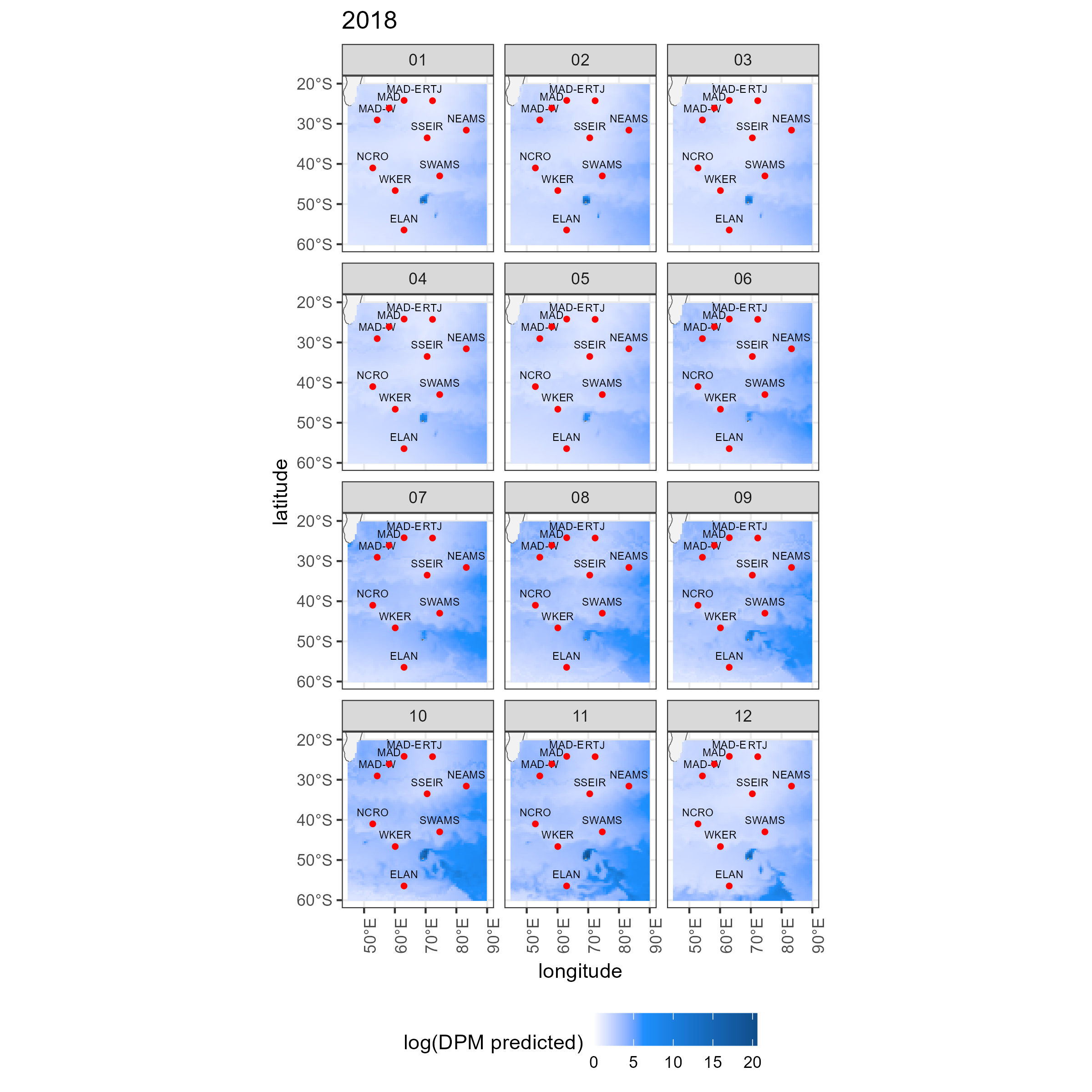

Supplement: Supplementary file 1 — Supplementary Information. [file 41598_2025_2941_MOESM1_ESM.zip › supp fig/antbw/plot_prediction_antbw_2018.png]

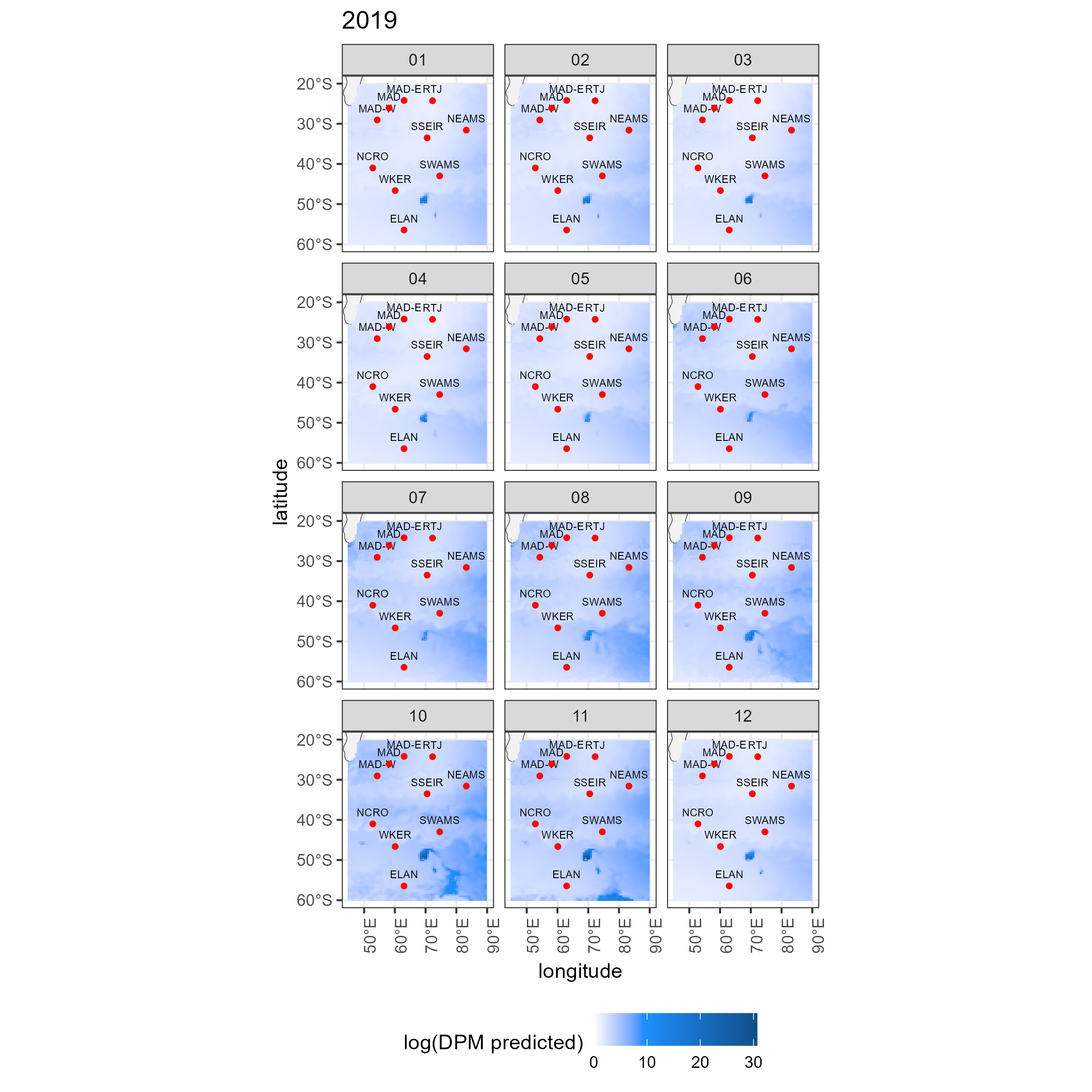

Supplement: Supplementary file 1 — Supplementary Information. [file 41598_2025_2941_MOESM1_ESM.zip › supp fig/antbw/plot_prediction_antbw_2019.png]

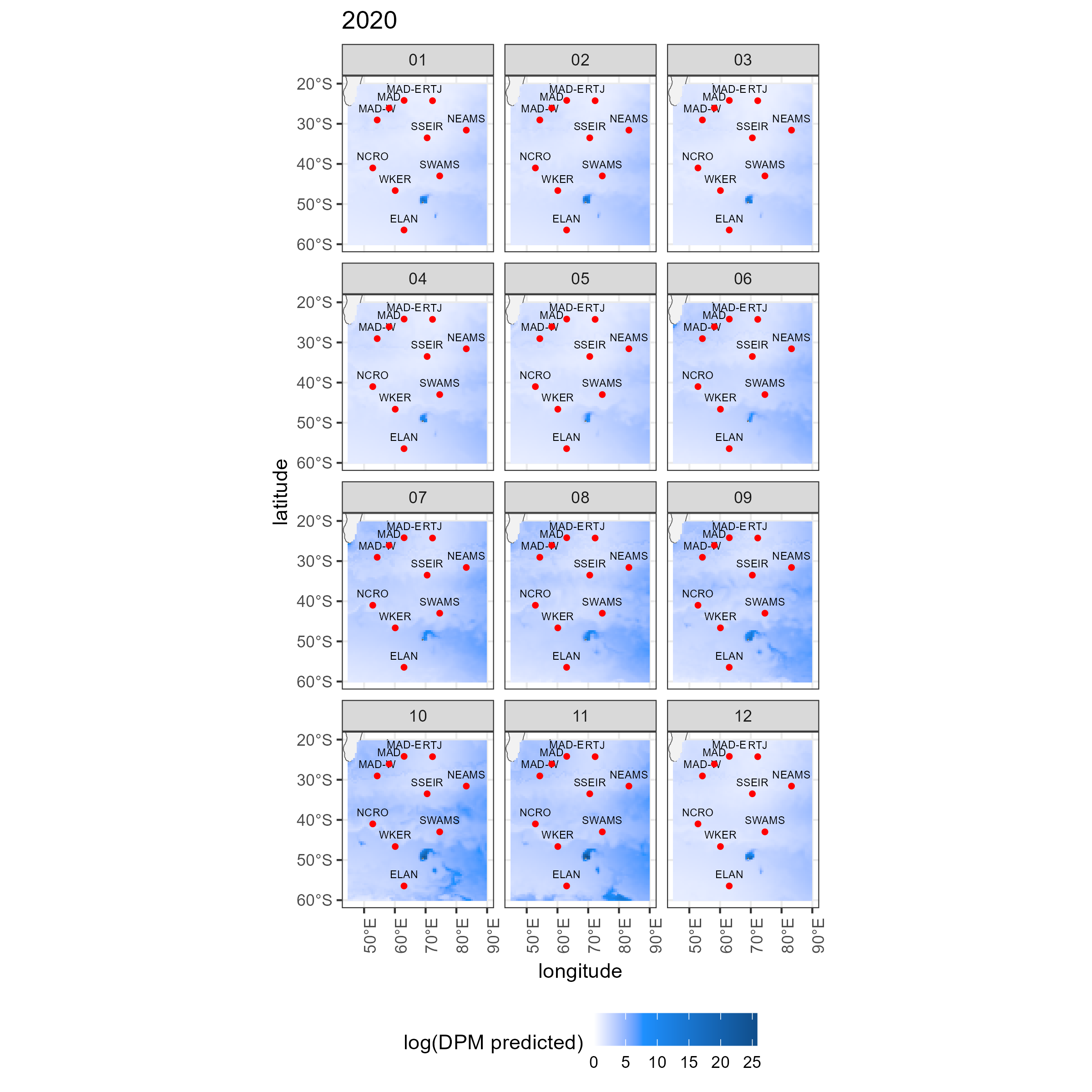

Supplement: Supplementary file 1 — Supplementary Information. [file 41598_2025_2941_MOESM1_ESM.zip › supp fig/antbw/plot_prediction_antbw_2020.png]

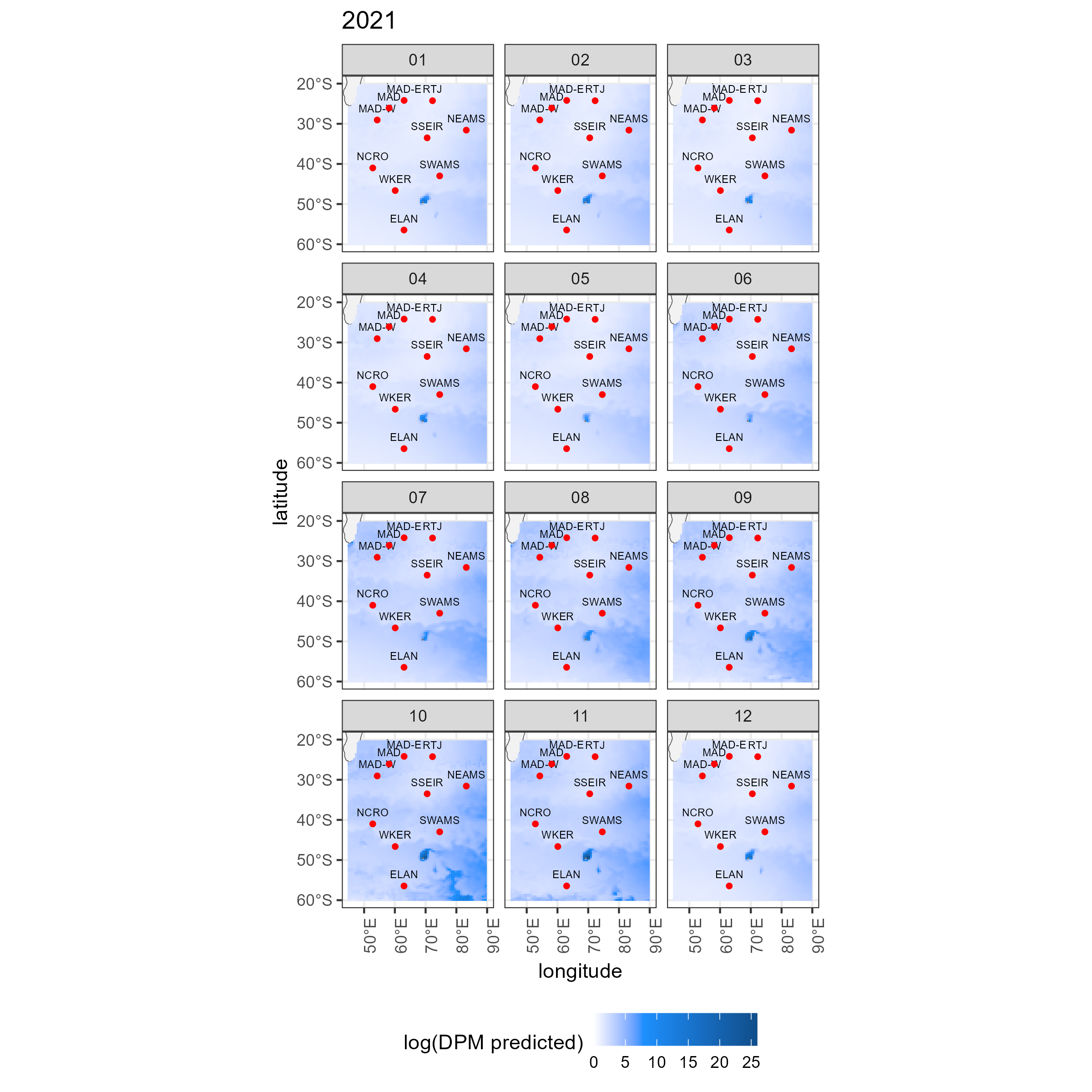

Supplement: Supplementary file 1 — Supplementary Information. [file 41598_2025_2941_MOESM1_ESM.zip › supp fig/antbw/plot_prediction_antbw_2021.png]

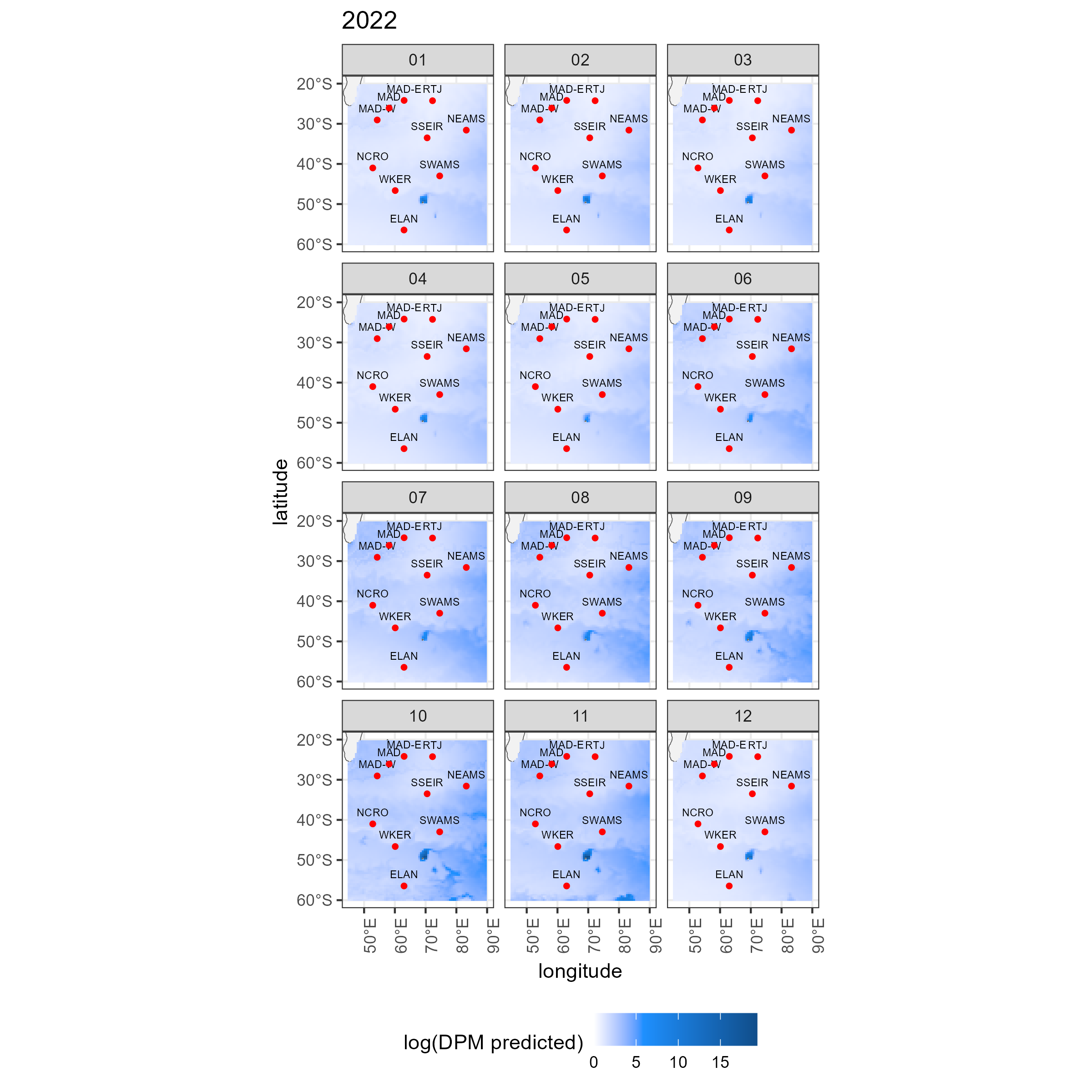

Supplement: Supplementary file 1 — Supplementary Information. [file 41598_2025_2941_MOESM1_ESM.zip › supp fig/antbw/plot_prediction_antbw_2022.png]

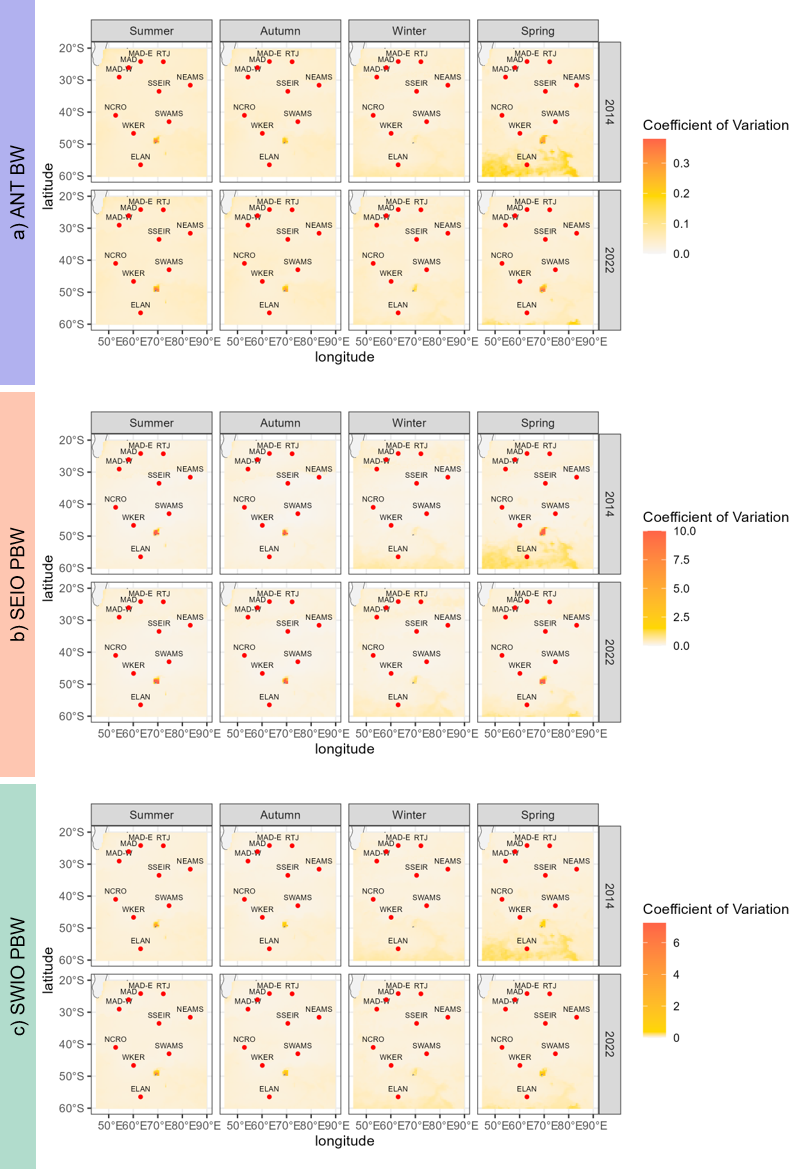

Supplement: Supplementary file 1 — Supplementary Information. [file 41598_2025_2941_MOESM1_ESM.zip › supp fig/combined_plots_cv.png]

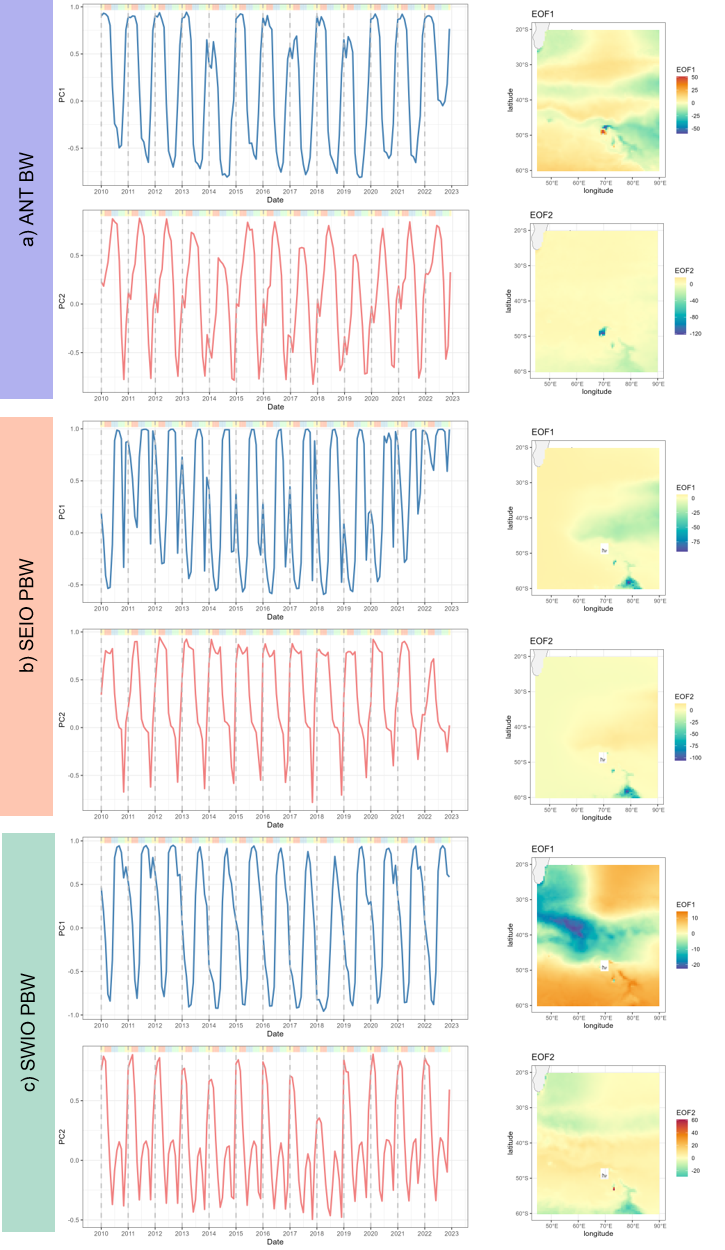

Supplement: Supplementary file 1 — Supplementary Information. [file 41598_2025_2941_MOESM1_ESM.zip › supp fig/combined_plots_EOF_all_species.png]

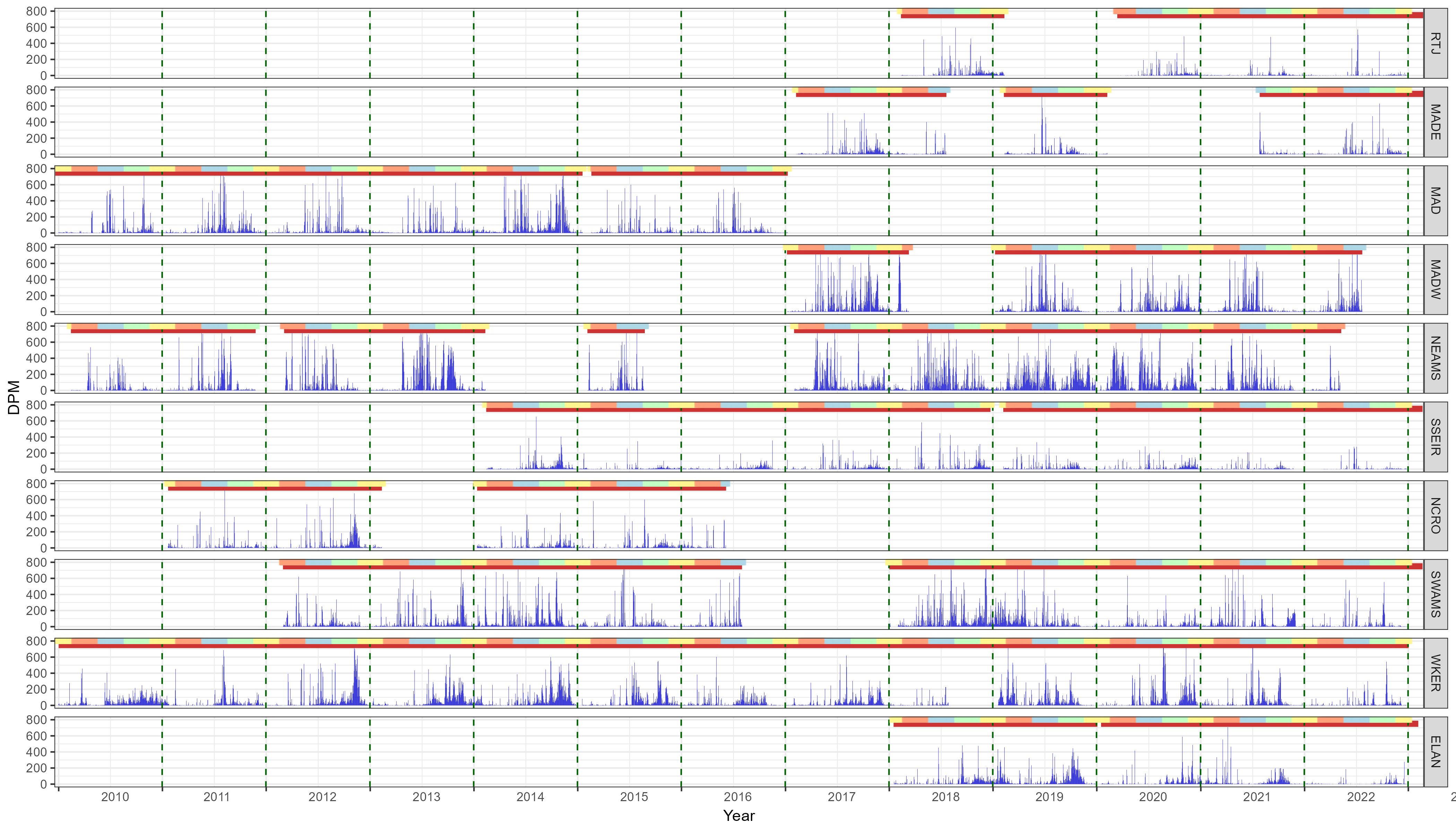

Supplement: Supplementary file 1 — Supplementary Information. [file 41598_2025_2941_MOESM1_ESM.zip › supp fig/plot_antbw.png]

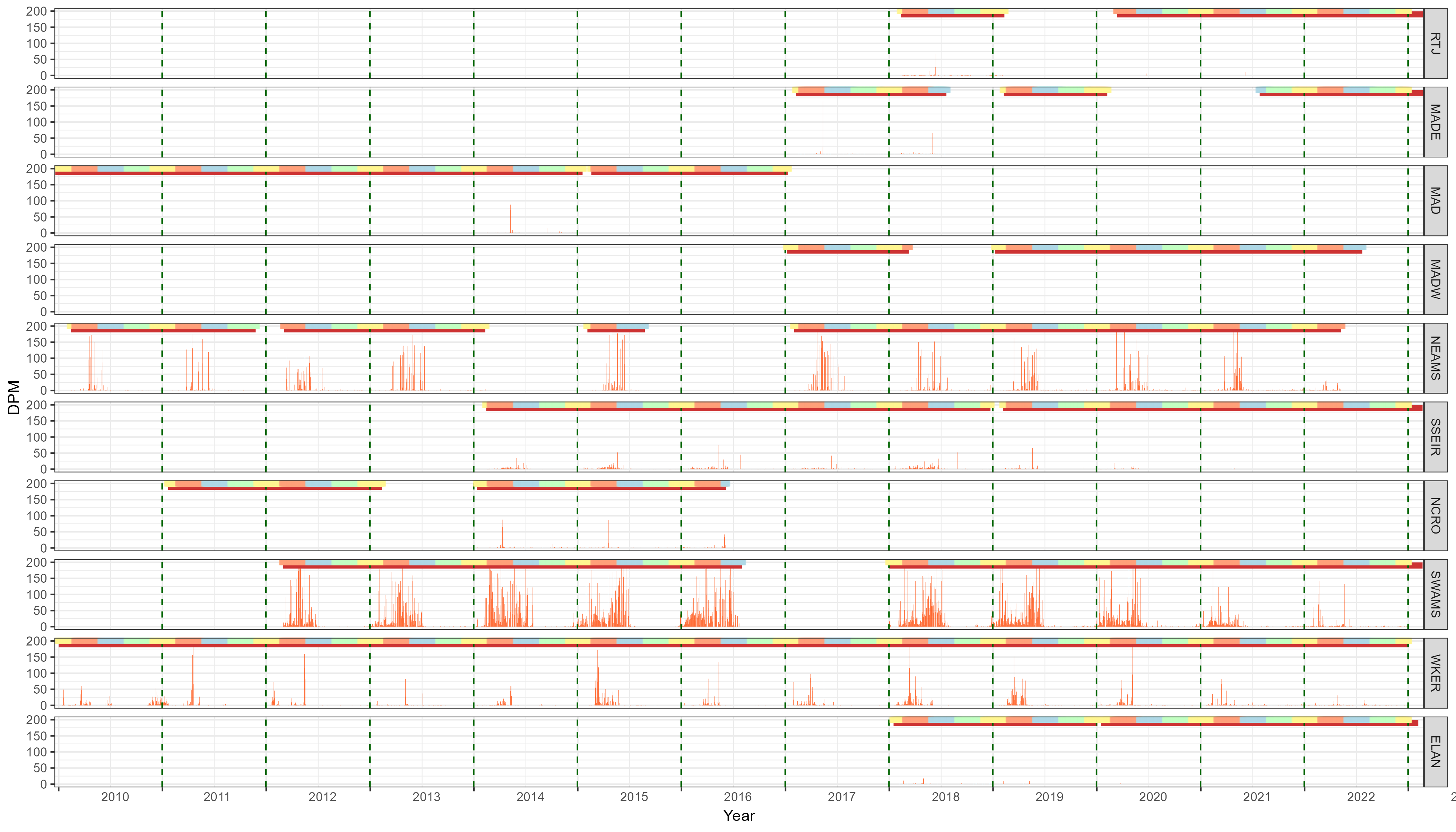

Supplement: Supplementary file 1 — Supplementary Information. [file 41598_2025_2941_MOESM1_ESM.zip › supp fig/plot_auspbw.png]

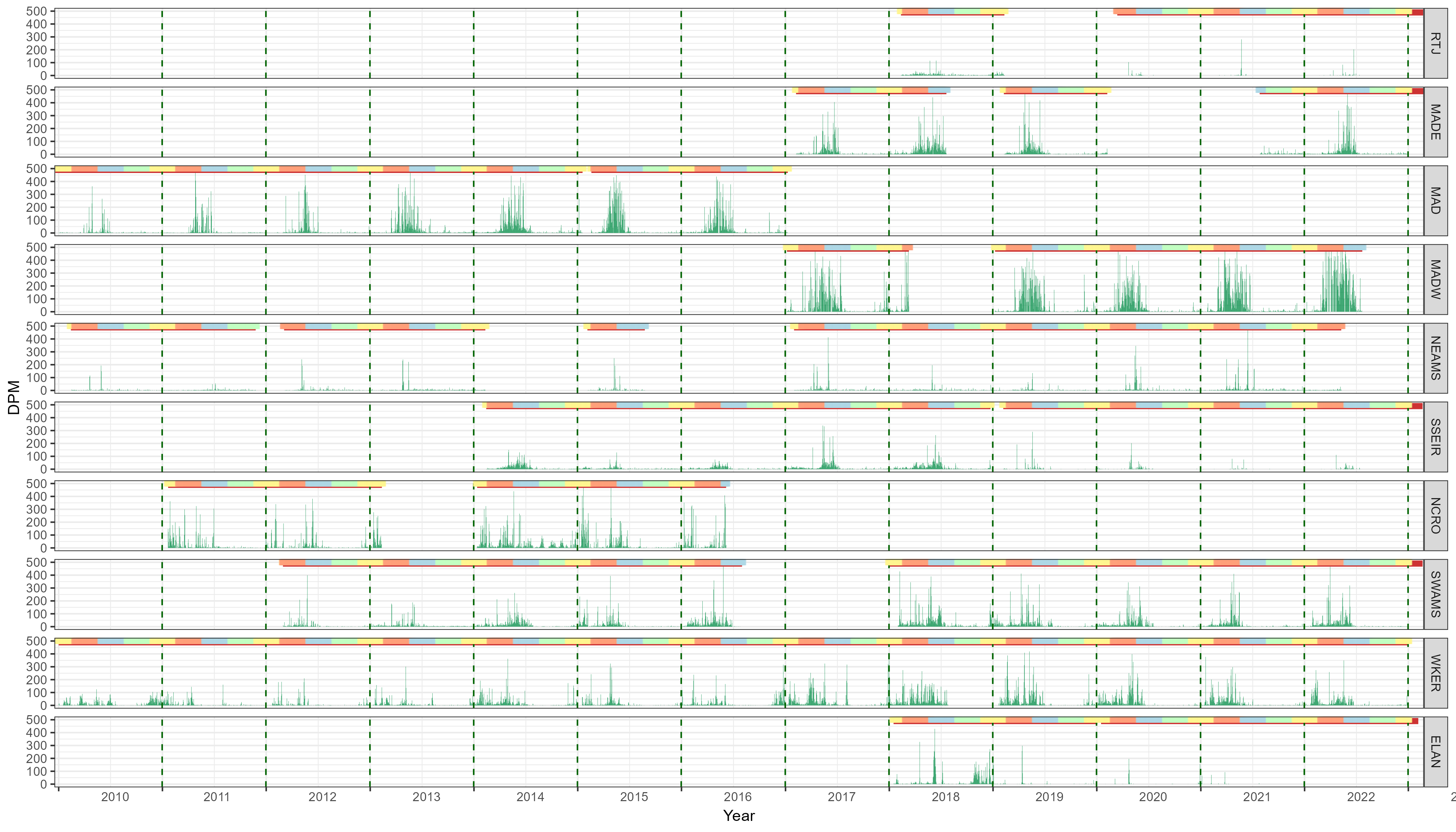

Supplement: Supplementary file 1 — Supplementary Information. [file 41598_2025_2941_MOESM1_ESM.zip › supp fig/plot_madpbw.png]

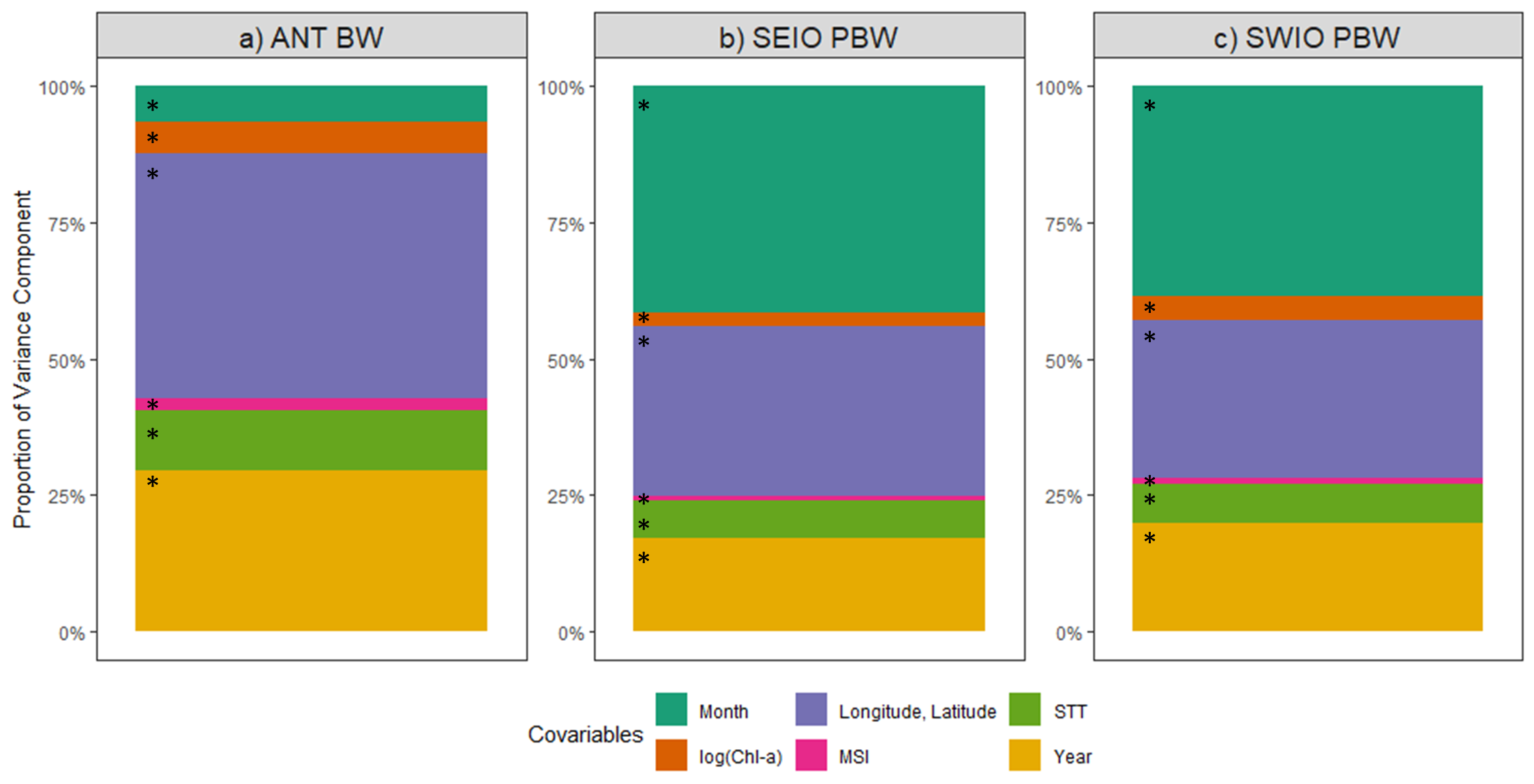

Supplement: Supplementary file 1 — Supplementary Information. [file 41598_2025_2941_MOESM1_ESM.zip › supp fig/proportion covariables.png]

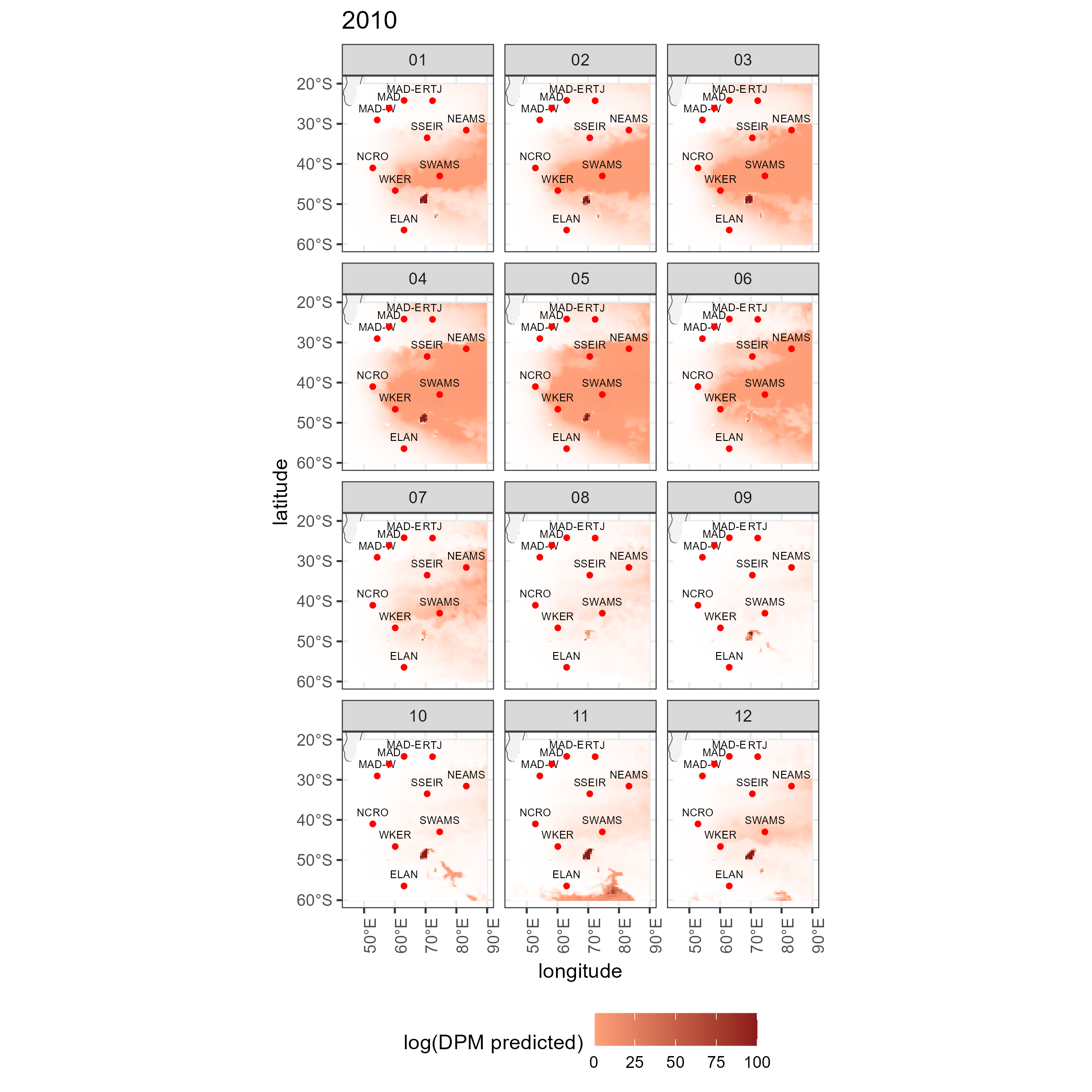

Supplement: Supplementary file 1 — Supplementary Information. [file 41598_2025_2941_MOESM1_ESM.zip › supp fig/seiopbw/plot_prediction_auspbw_2010.png]

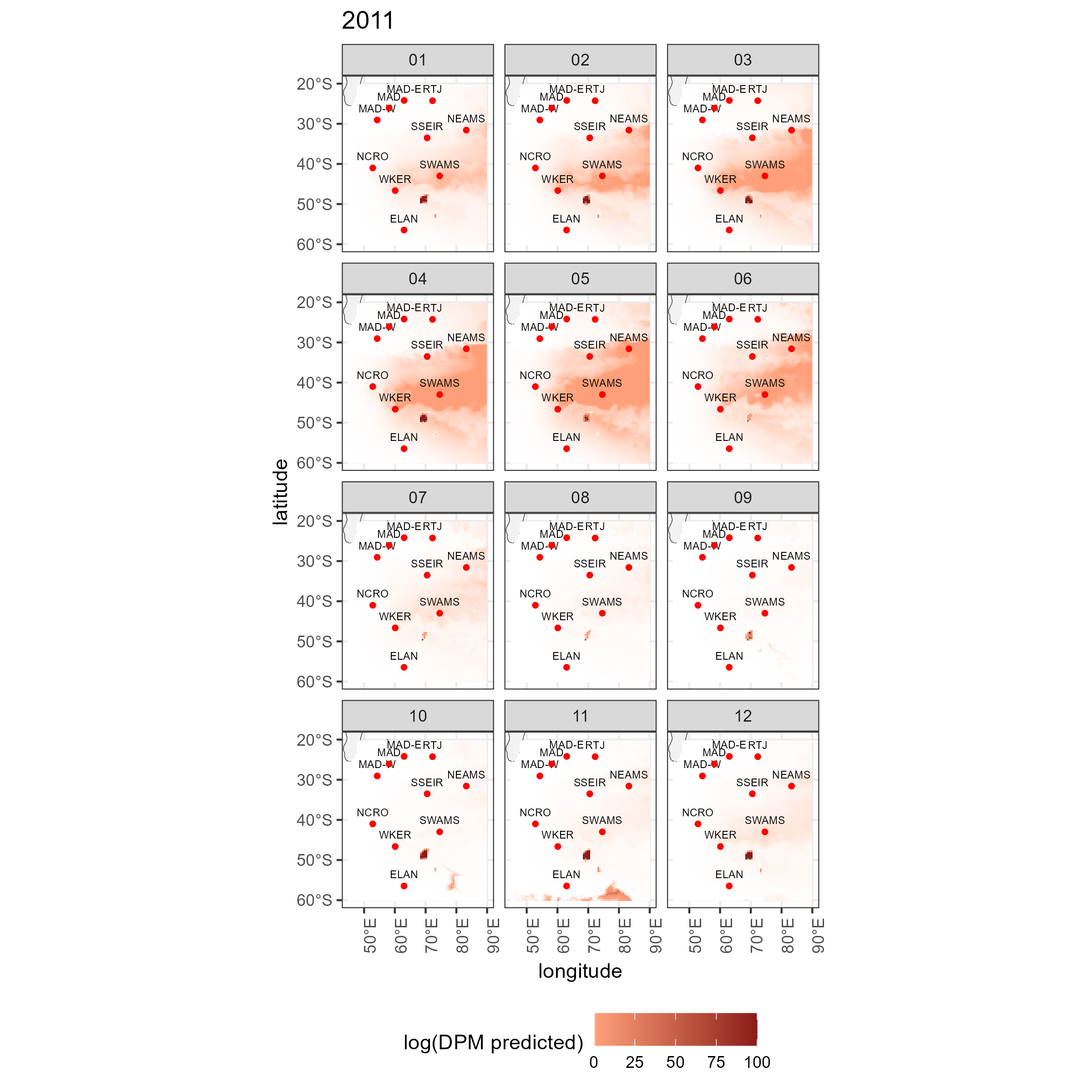

Supplement: Supplementary file 1 — Supplementary Information. [file 41598_2025_2941_MOESM1_ESM.zip › supp fig/seiopbw/plot_prediction_auspbw_2011.png]

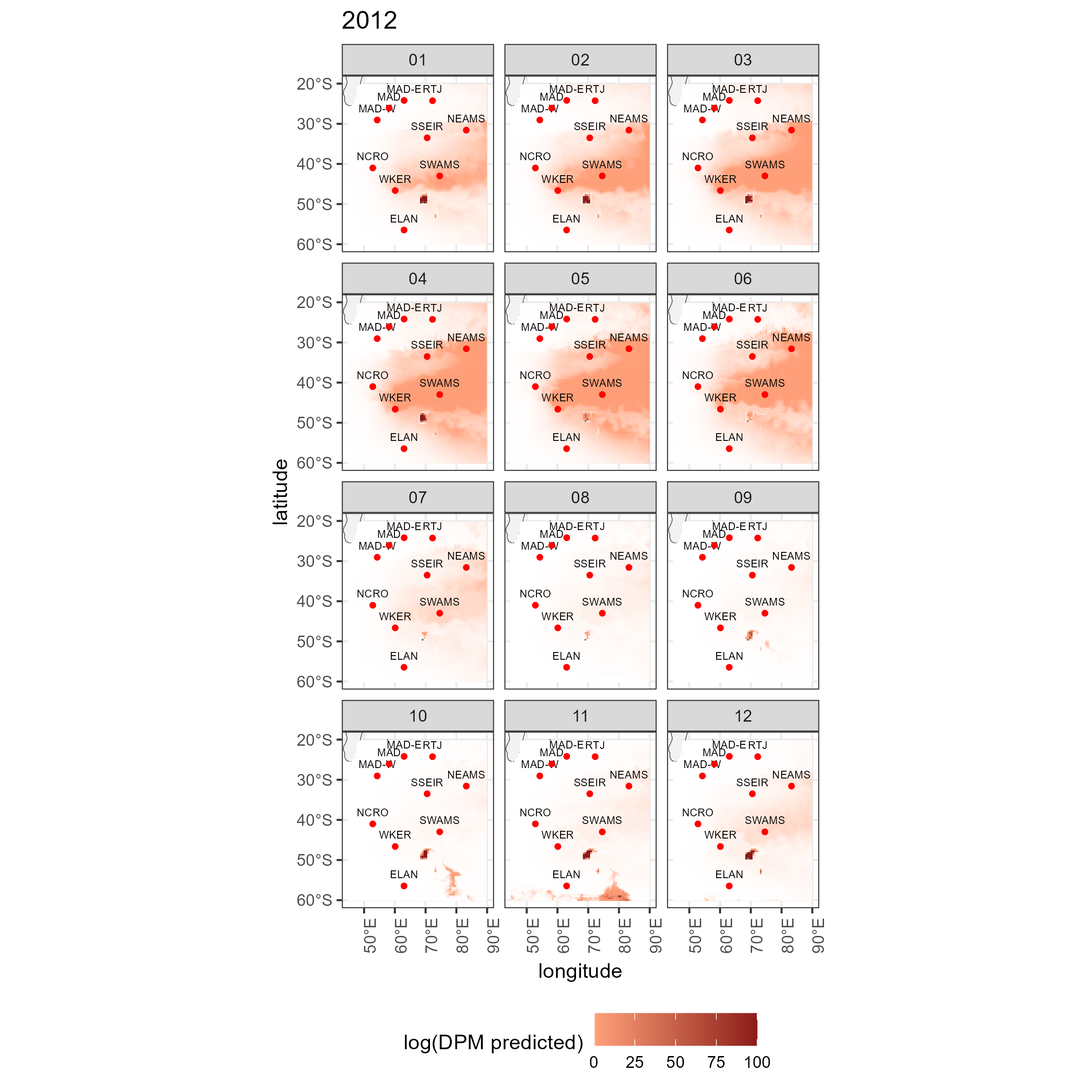

Supplement: Supplementary file 1 — Supplementary Information. [file 41598_2025_2941_MOESM1_ESM.zip › supp fig/seiopbw/plot_prediction_auspbw_2012.png]

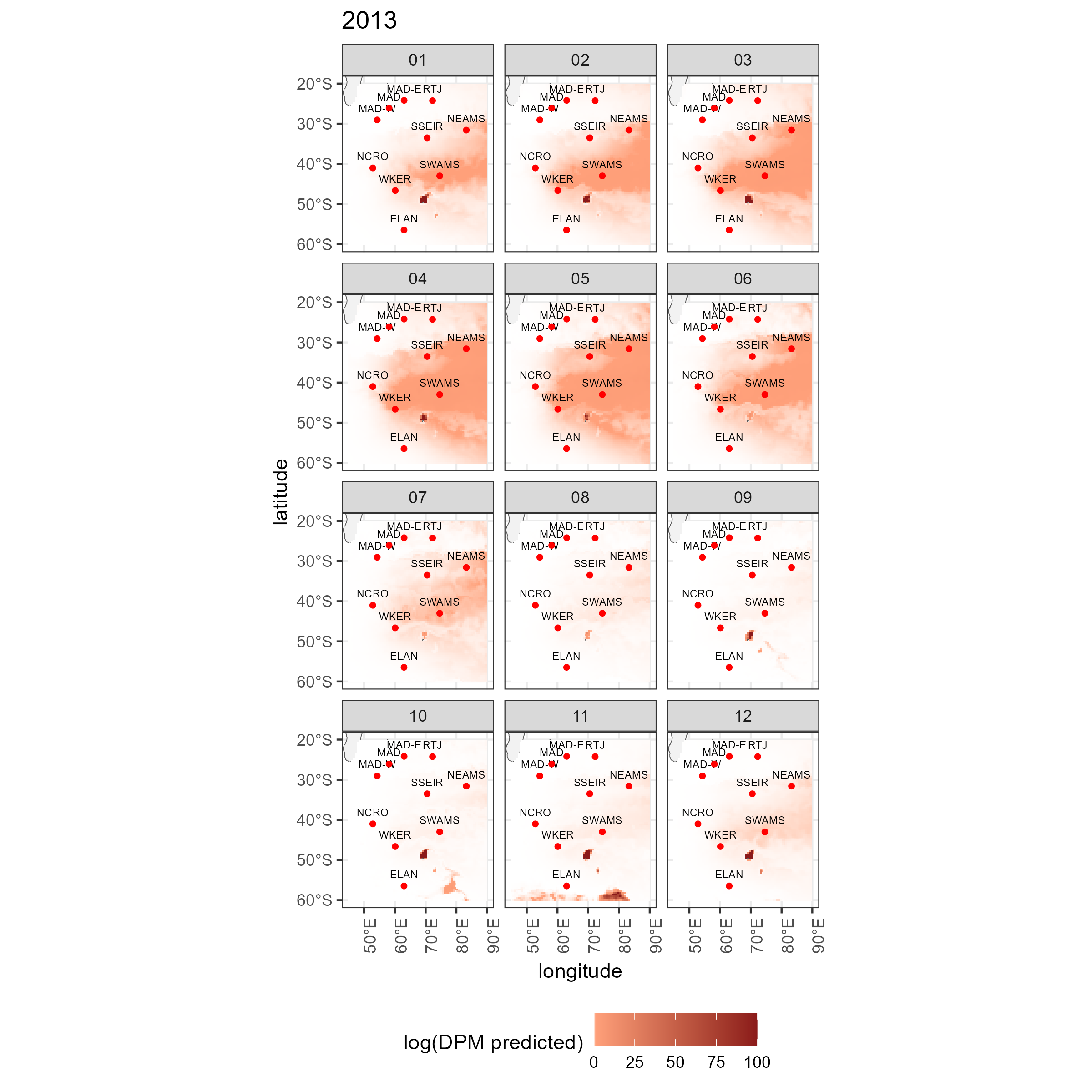

Supplement: Supplementary file 1 — Supplementary Information. [file 41598_2025_2941_MOESM1_ESM.zip › supp fig/seiopbw/plot_prediction_auspbw_2013.png]

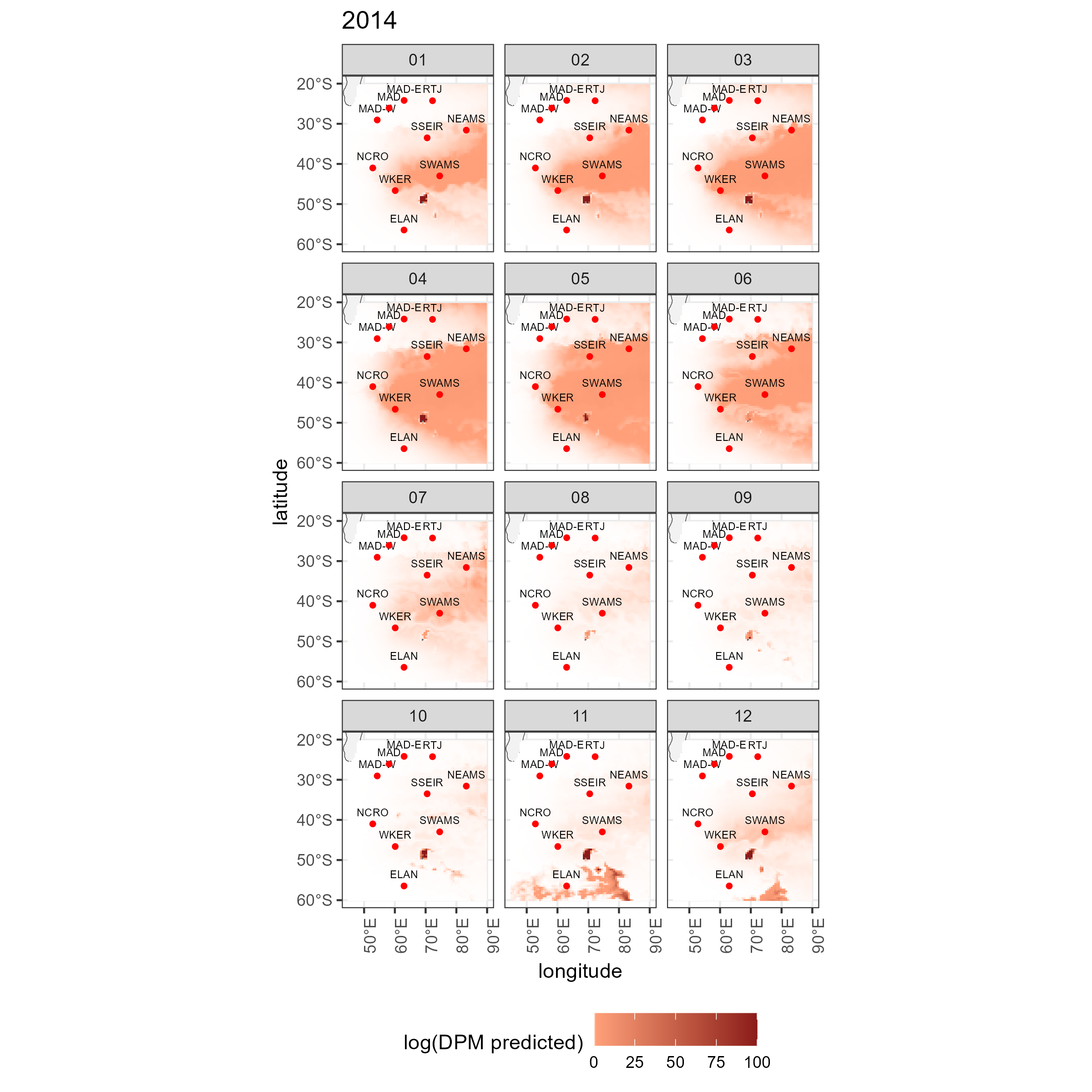

Supplement: Supplementary file 1 — Supplementary Information. [file 41598_2025_2941_MOESM1_ESM.zip › supp fig/seiopbw/plot_prediction_auspbw_2014.png]

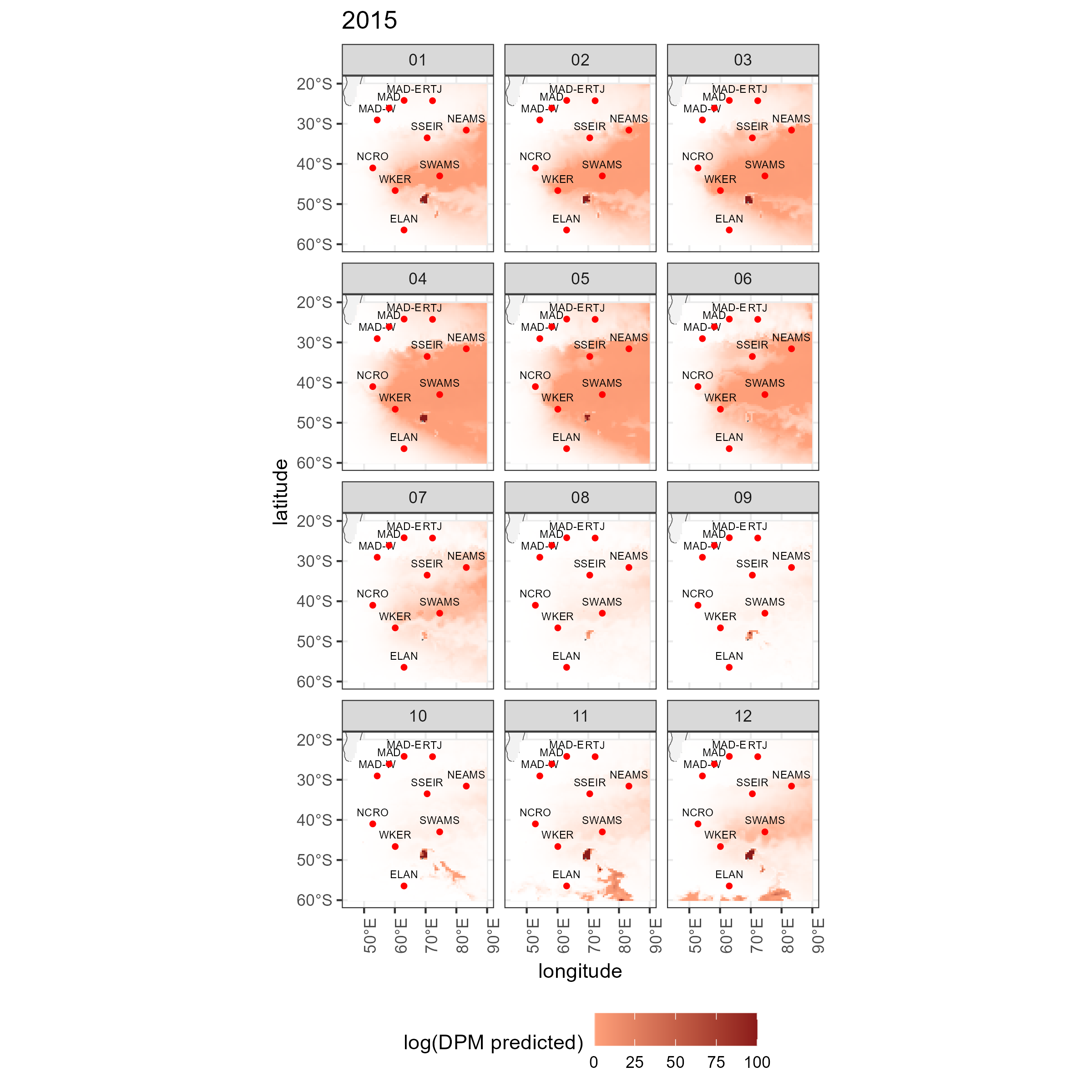

Supplement: Supplementary file 1 — Supplementary Information. [file 41598_2025_2941_MOESM1_ESM.zip › supp fig/seiopbw/plot_prediction_auspbw_2015.png]

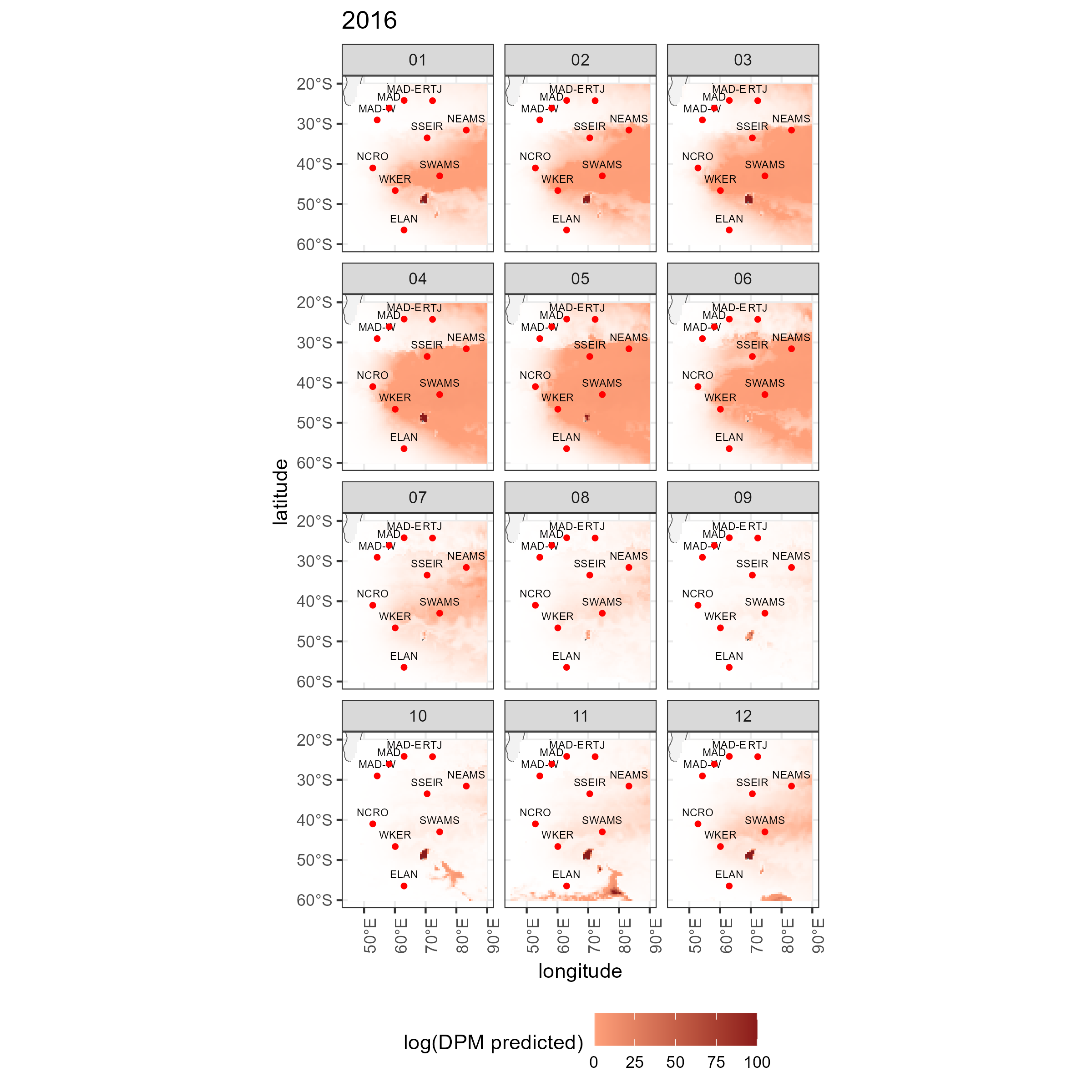

Supplement: Supplementary file 1 — Supplementary Information. [file 41598_2025_2941_MOESM1_ESM.zip › supp fig/seiopbw/plot_prediction_auspbw_2016.png]

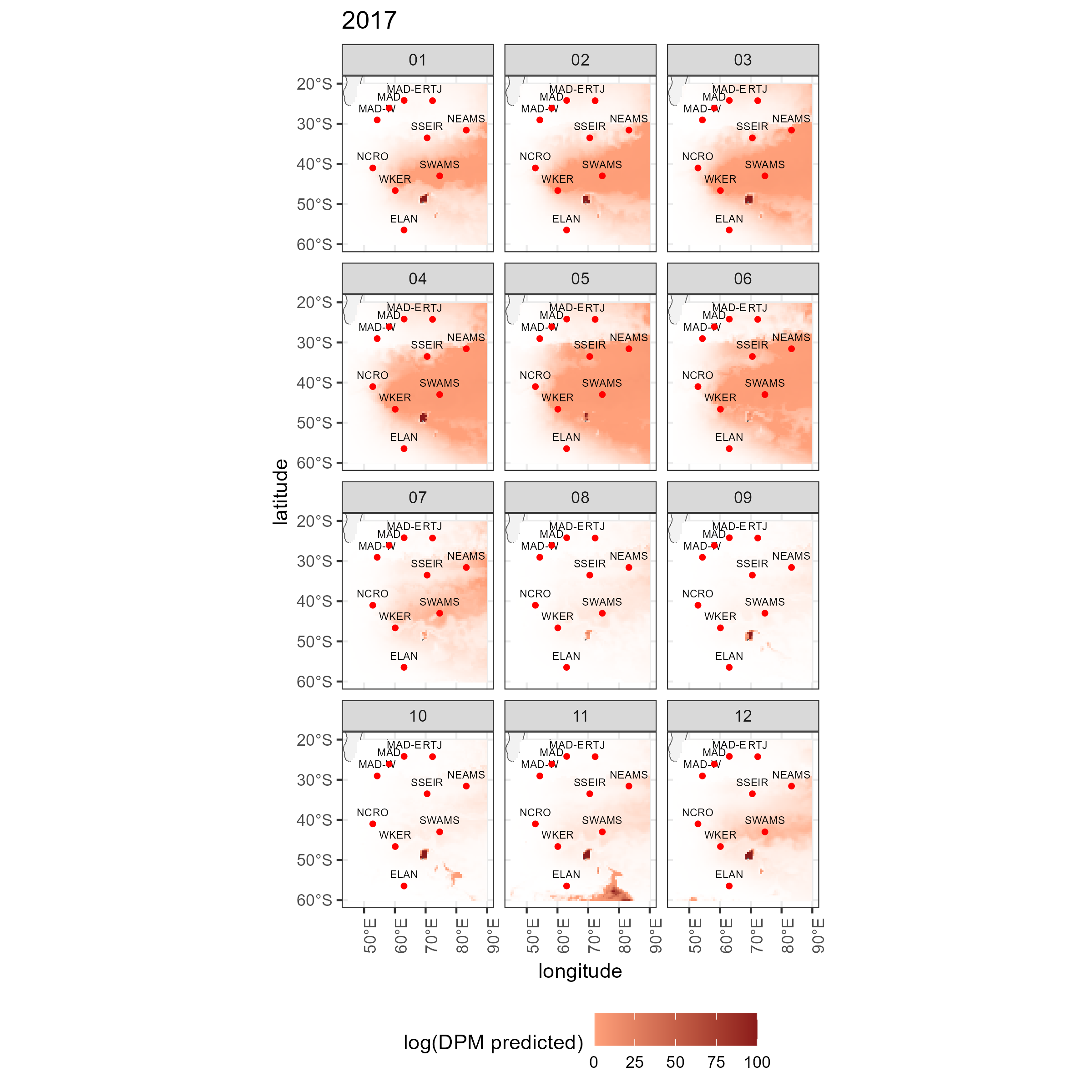

Supplement: Supplementary file 1 — Supplementary Information. [file 41598_2025_2941_MOESM1_ESM.zip › supp fig/seiopbw/plot_prediction_auspbw_2017.png]

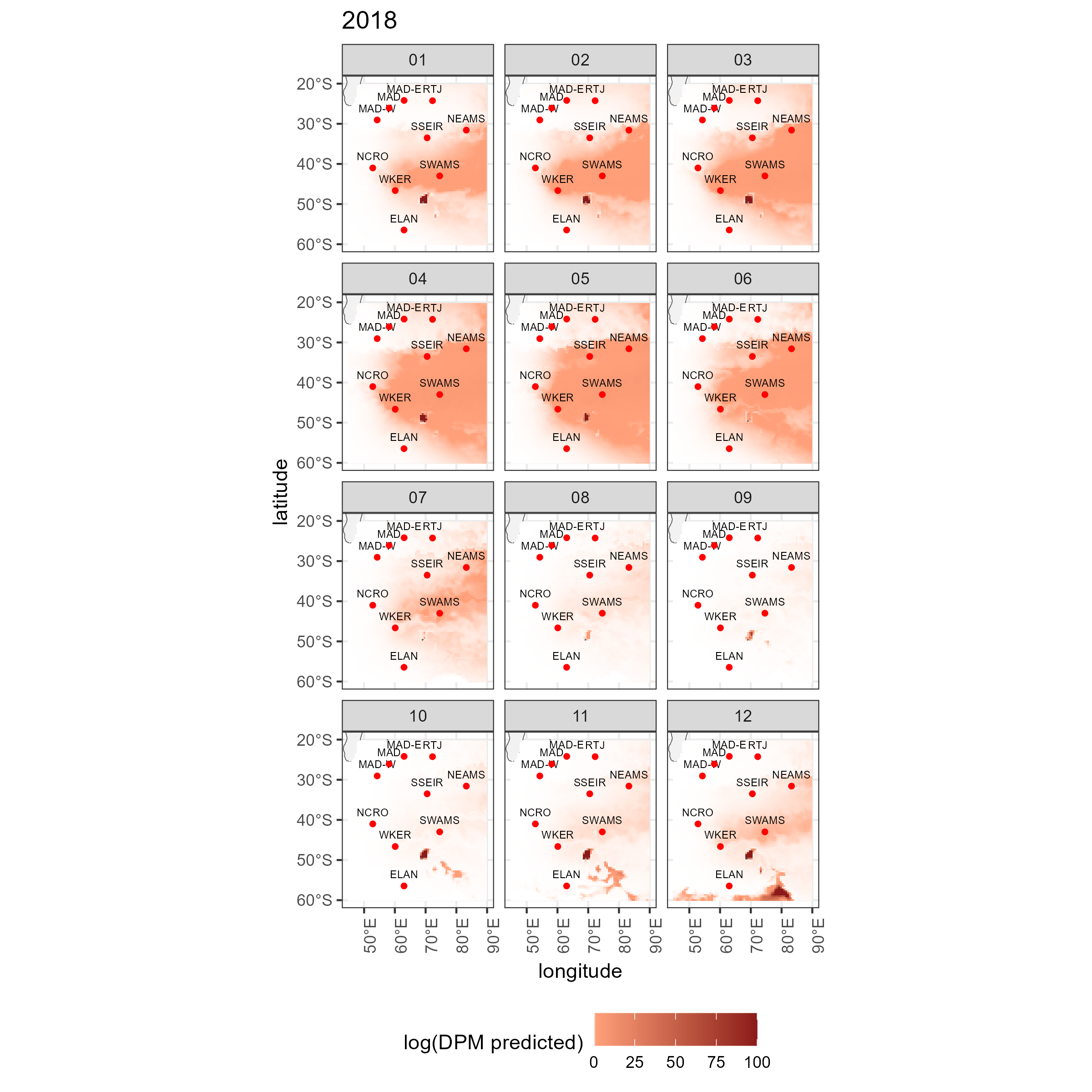

Supplement: Supplementary file 1 — Supplementary Information. [file 41598_2025_2941_MOESM1_ESM.zip › supp fig/seiopbw/plot_prediction_auspbw_2018.png]

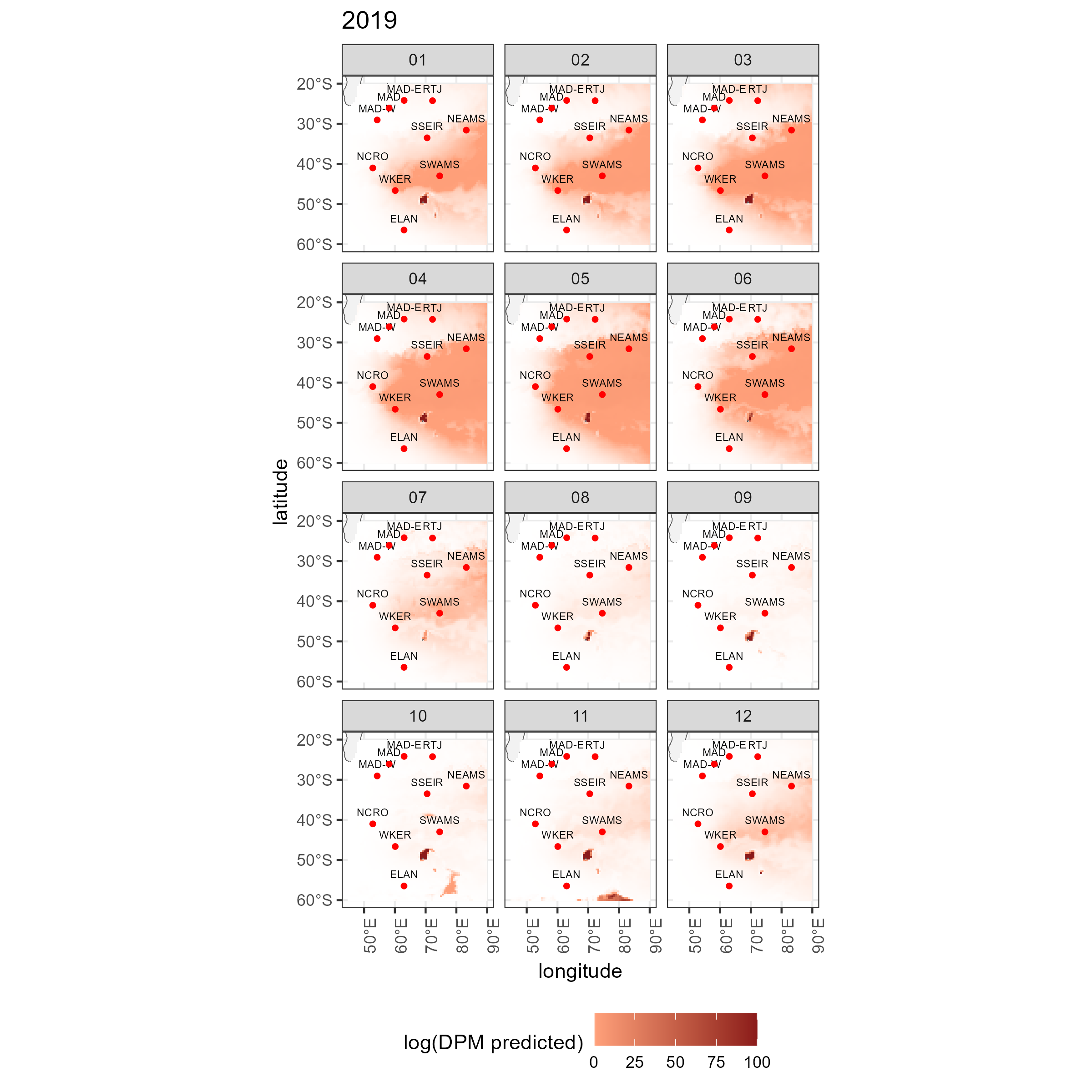

Supplement: Supplementary file 1 — Supplementary Information. [file 41598_2025_2941_MOESM1_ESM.zip › supp fig/seiopbw/plot_prediction_auspbw_2019.png]

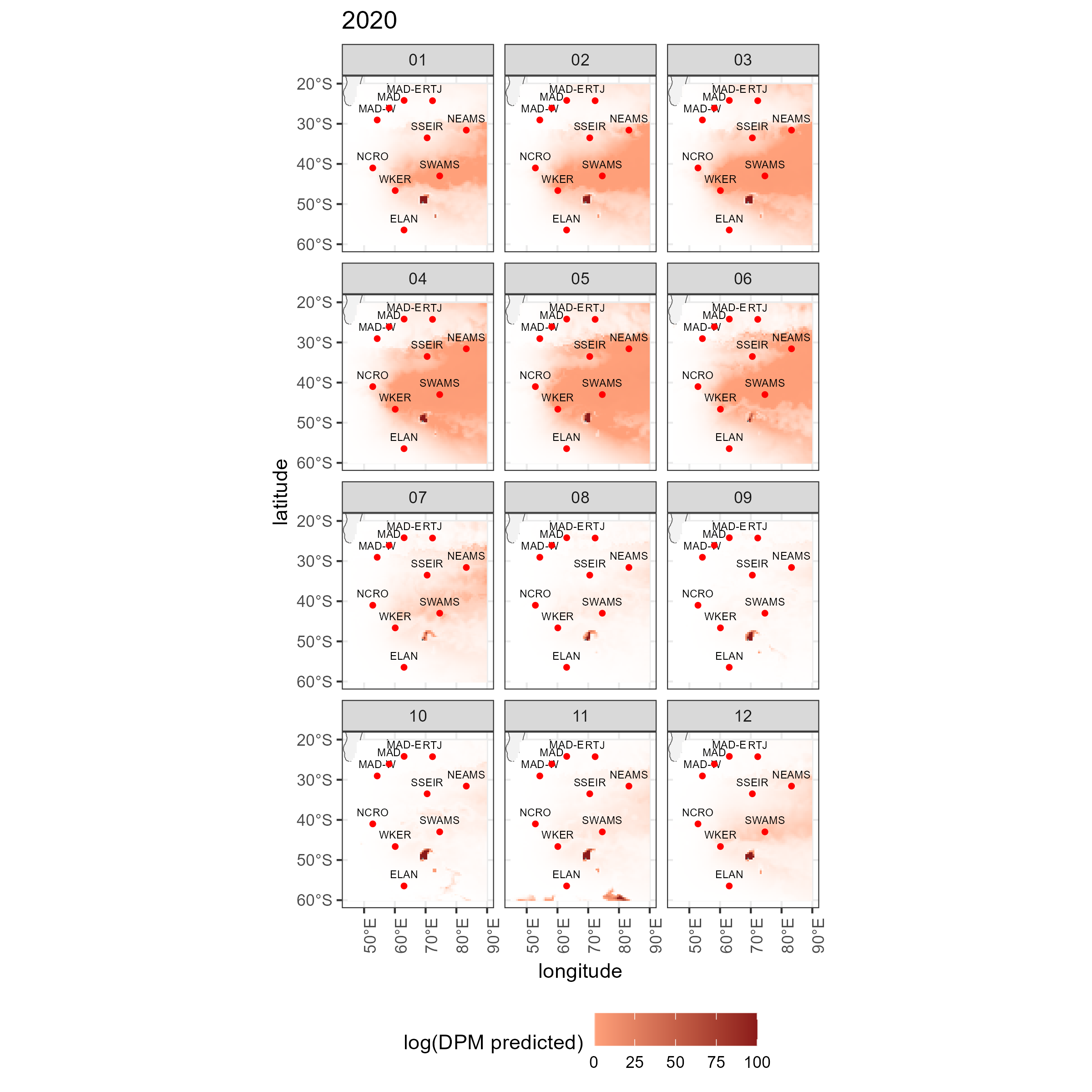

Supplement: Supplementary file 1 — Supplementary Information. [file 41598_2025_2941_MOESM1_ESM.zip › supp fig/seiopbw/plot_prediction_auspbw_2020.png]

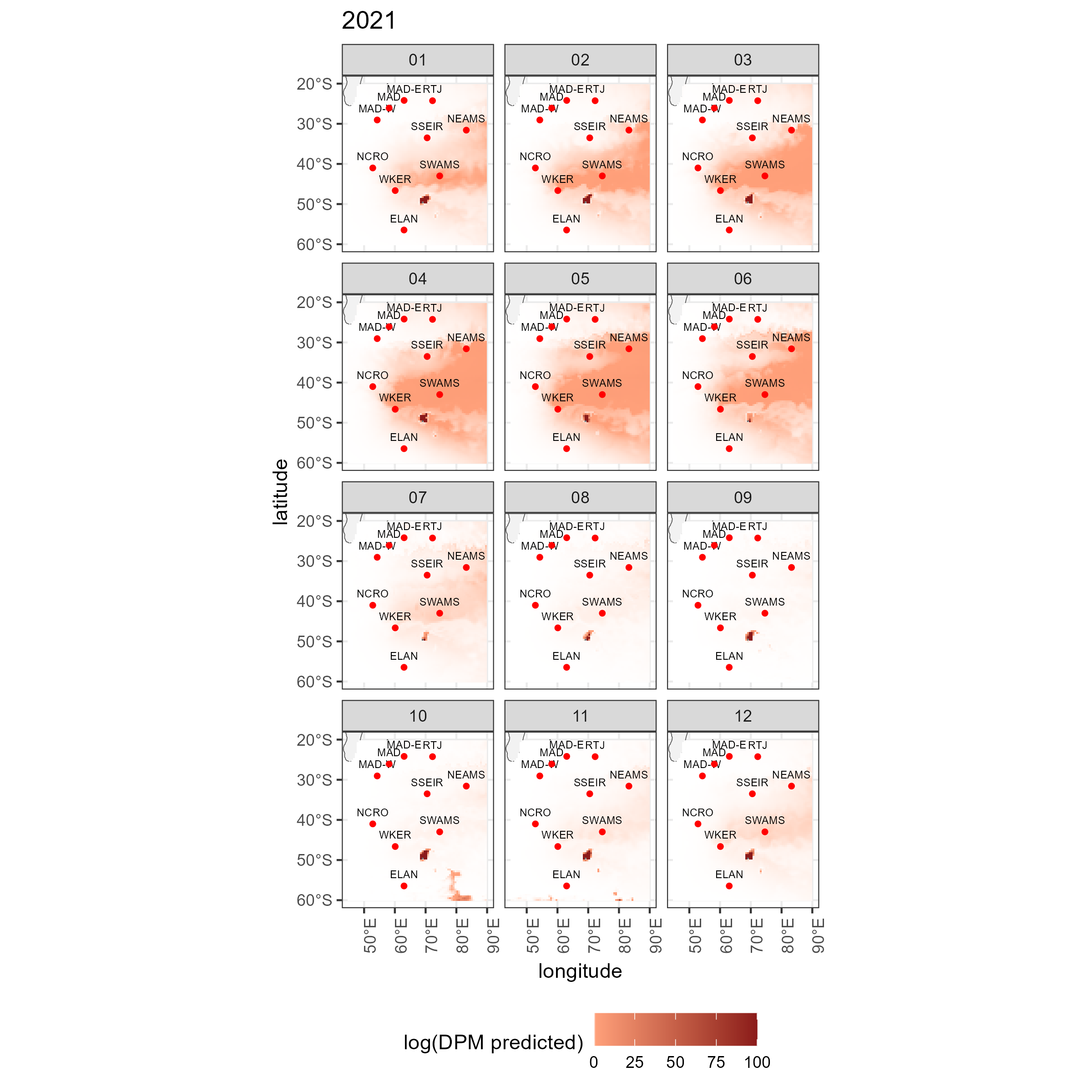

Supplement: Supplementary file 1 — Supplementary Information. [file 41598_2025_2941_MOESM1_ESM.zip › supp fig/seiopbw/plot_prediction_auspbw_2021.png]

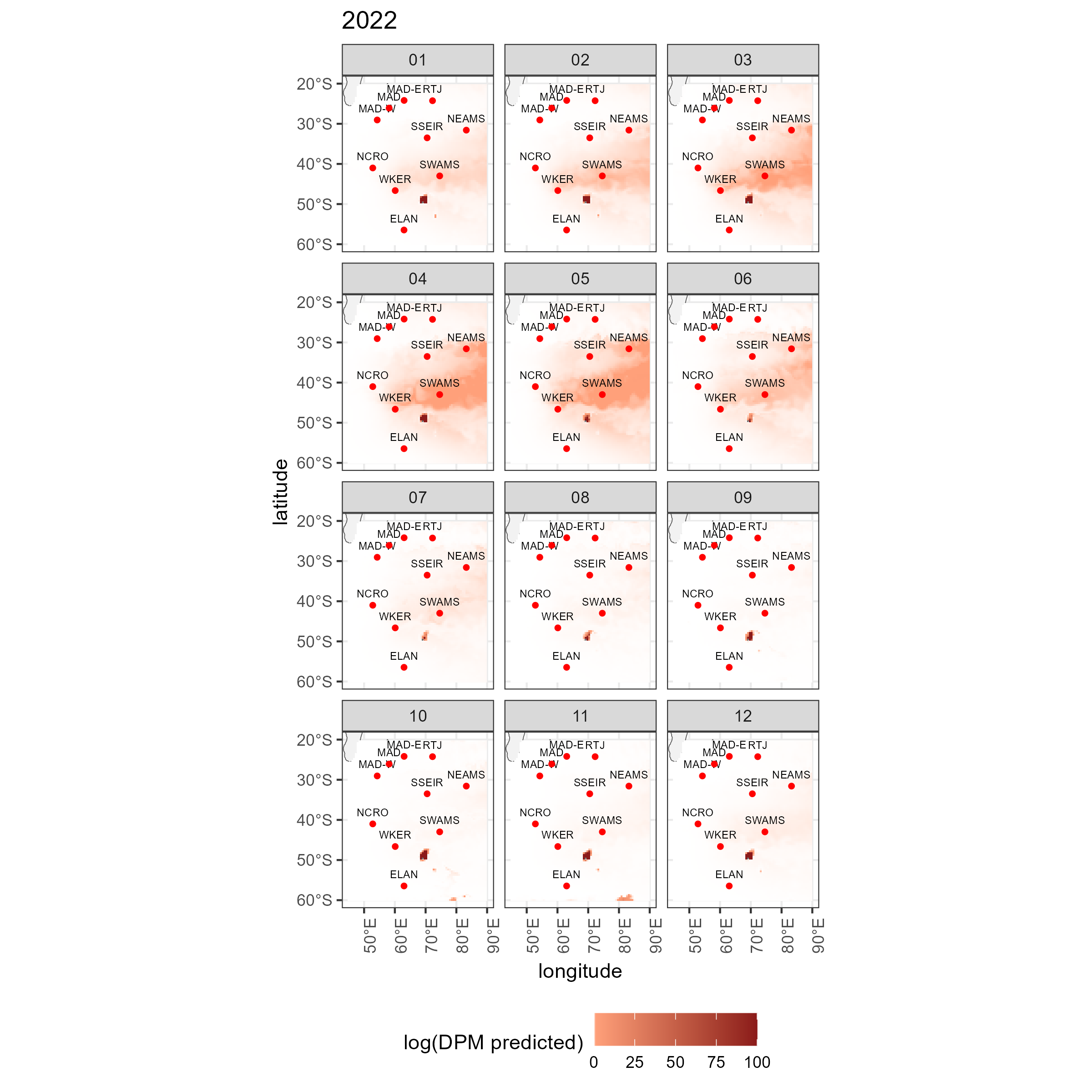

Supplement: Supplementary file 1 — Supplementary Information. [file 41598_2025_2941_MOESM1_ESM.zip › supp fig/seiopbw/plot_prediction_auspbw_2022.png]

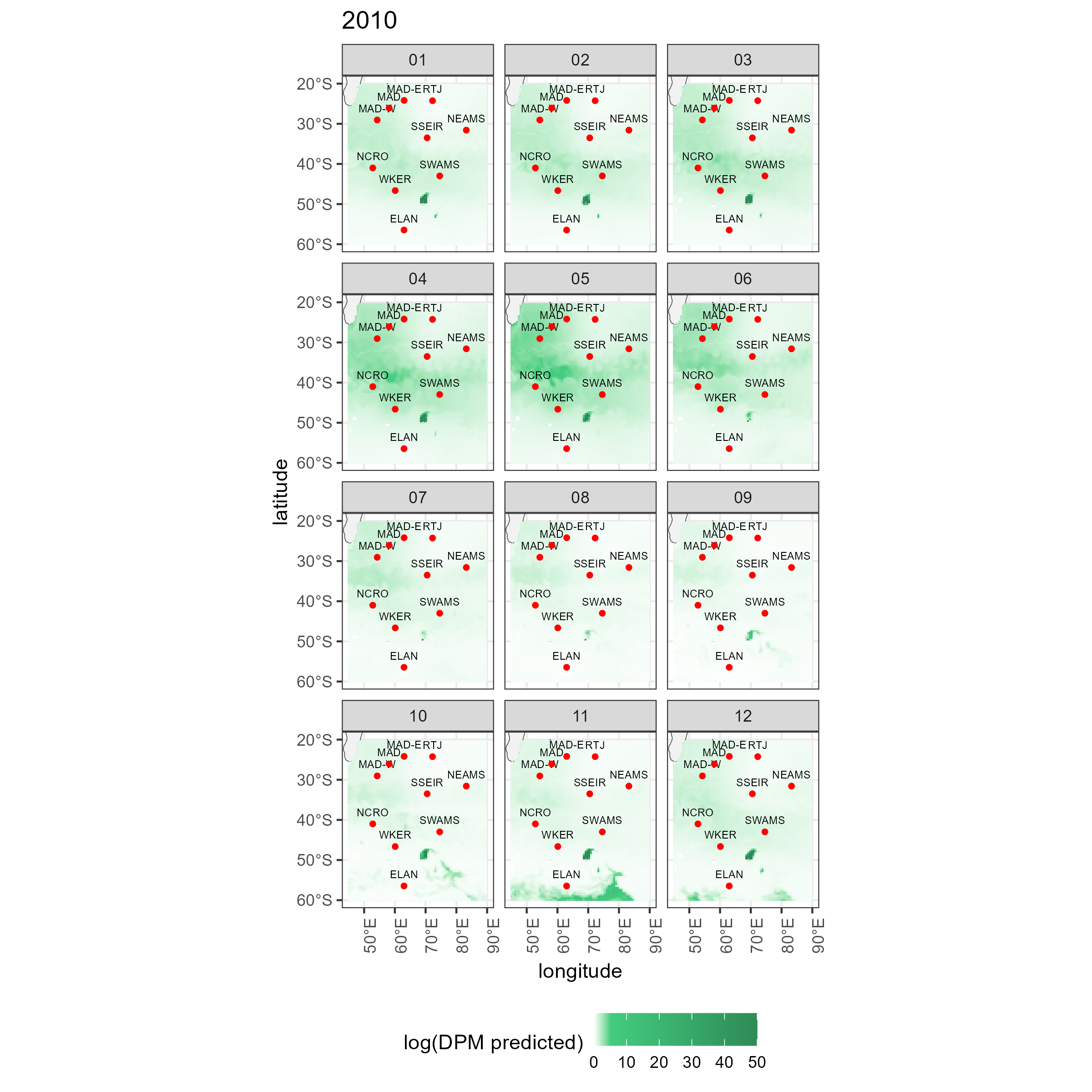

Supplement: Supplementary file 1 — Supplementary Information. [file 41598_2025_2941_MOESM1_ESM.zip › supp fig/swiopbw/plot_prediction_madpbw_2010.png]

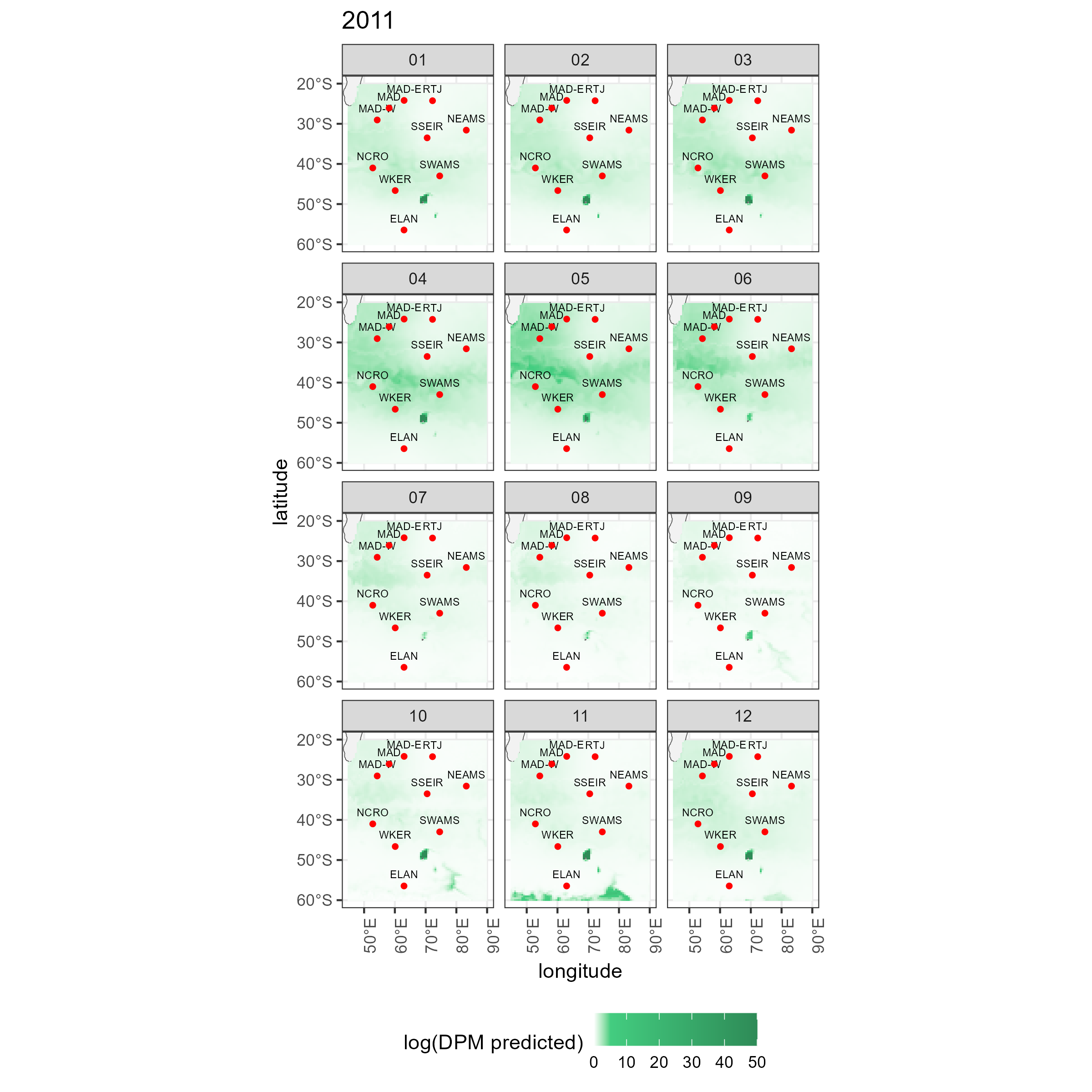

Supplement: Supplementary file 1 — Supplementary Information. [file 41598_2025_2941_MOESM1_ESM.zip › supp fig/swiopbw/plot_prediction_madpbw_2011.png]

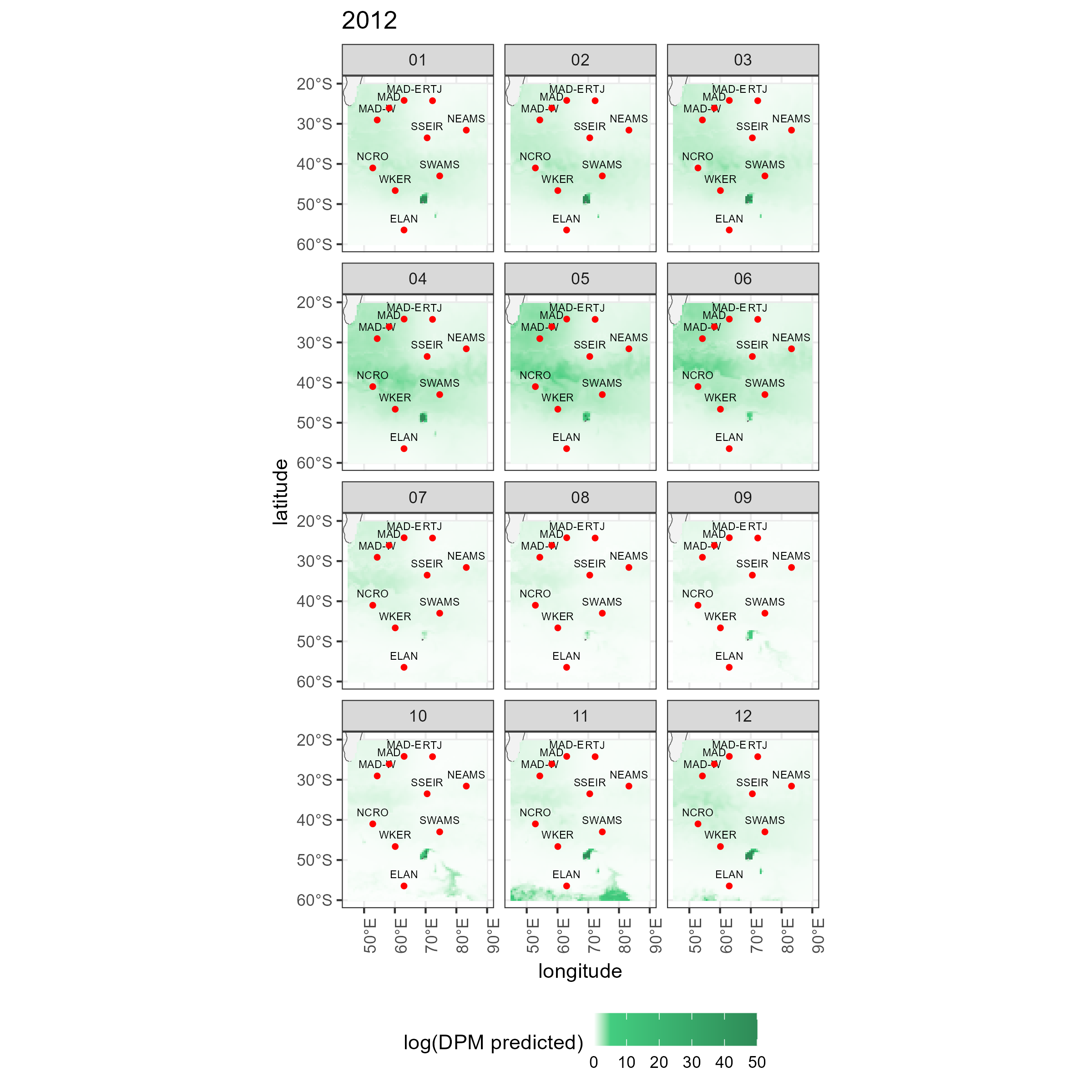

Supplement: Supplementary file 1 — Supplementary Information. [file 41598_2025_2941_MOESM1_ESM.zip › supp fig/swiopbw/plot_prediction_madpbw_2012.png]

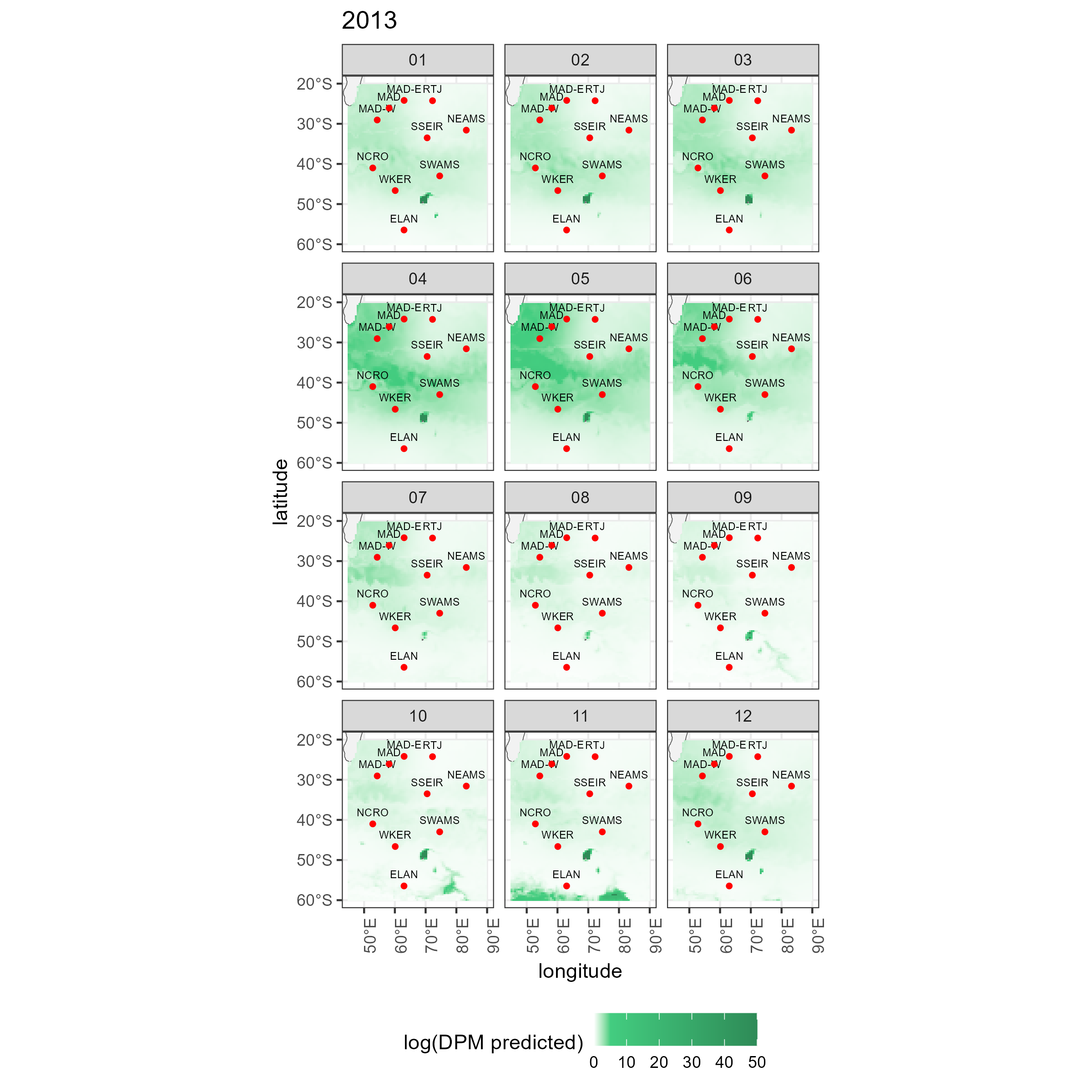

Supplement: Supplementary file 1 — Supplementary Information. [file 41598_2025_2941_MOESM1_ESM.zip › supp fig/swiopbw/plot_prediction_madpbw_2013.png]

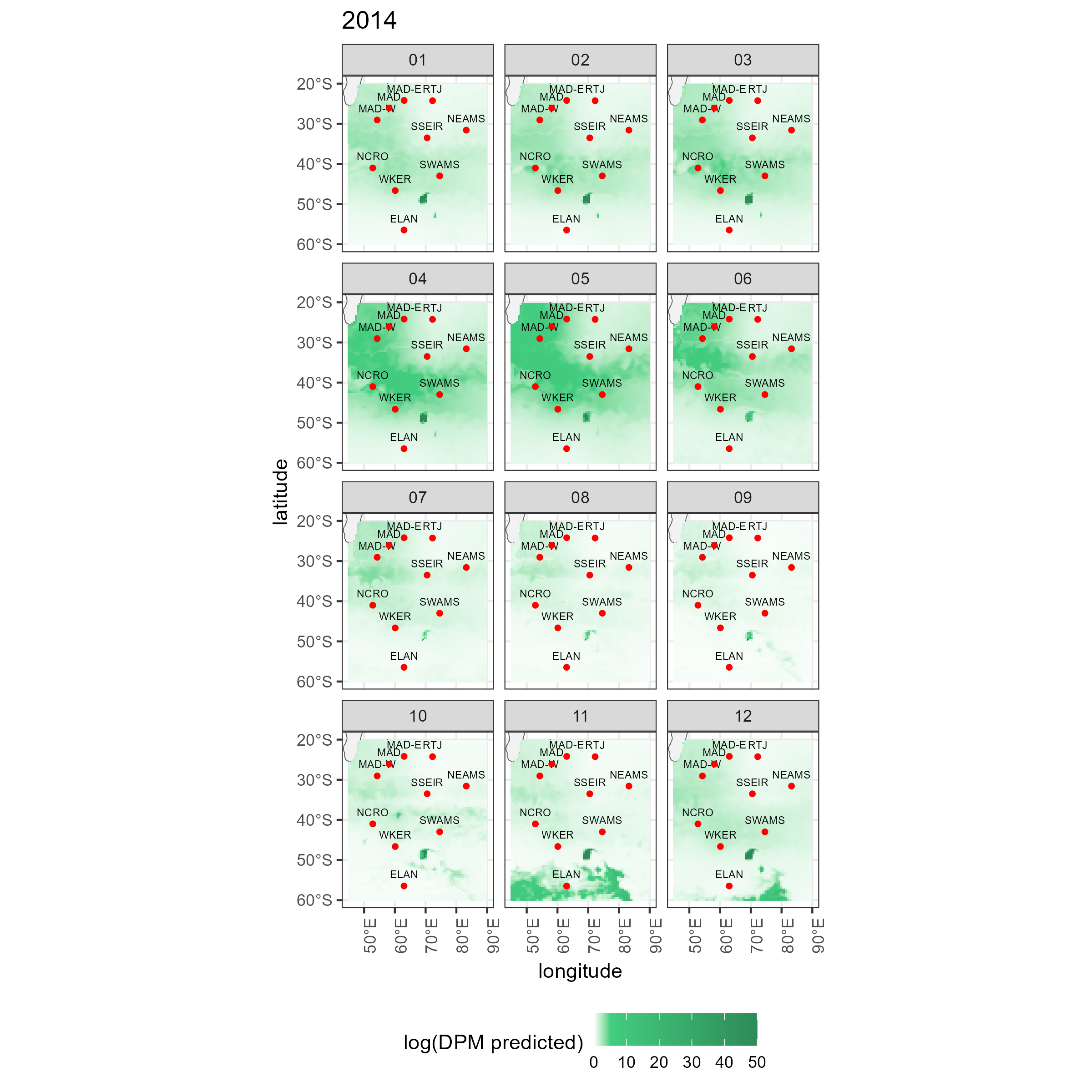

Supplement: Supplementary file 1 — Supplementary Information. [file 41598_2025_2941_MOESM1_ESM.zip › supp fig/swiopbw/plot_prediction_madpbw_2014.png]

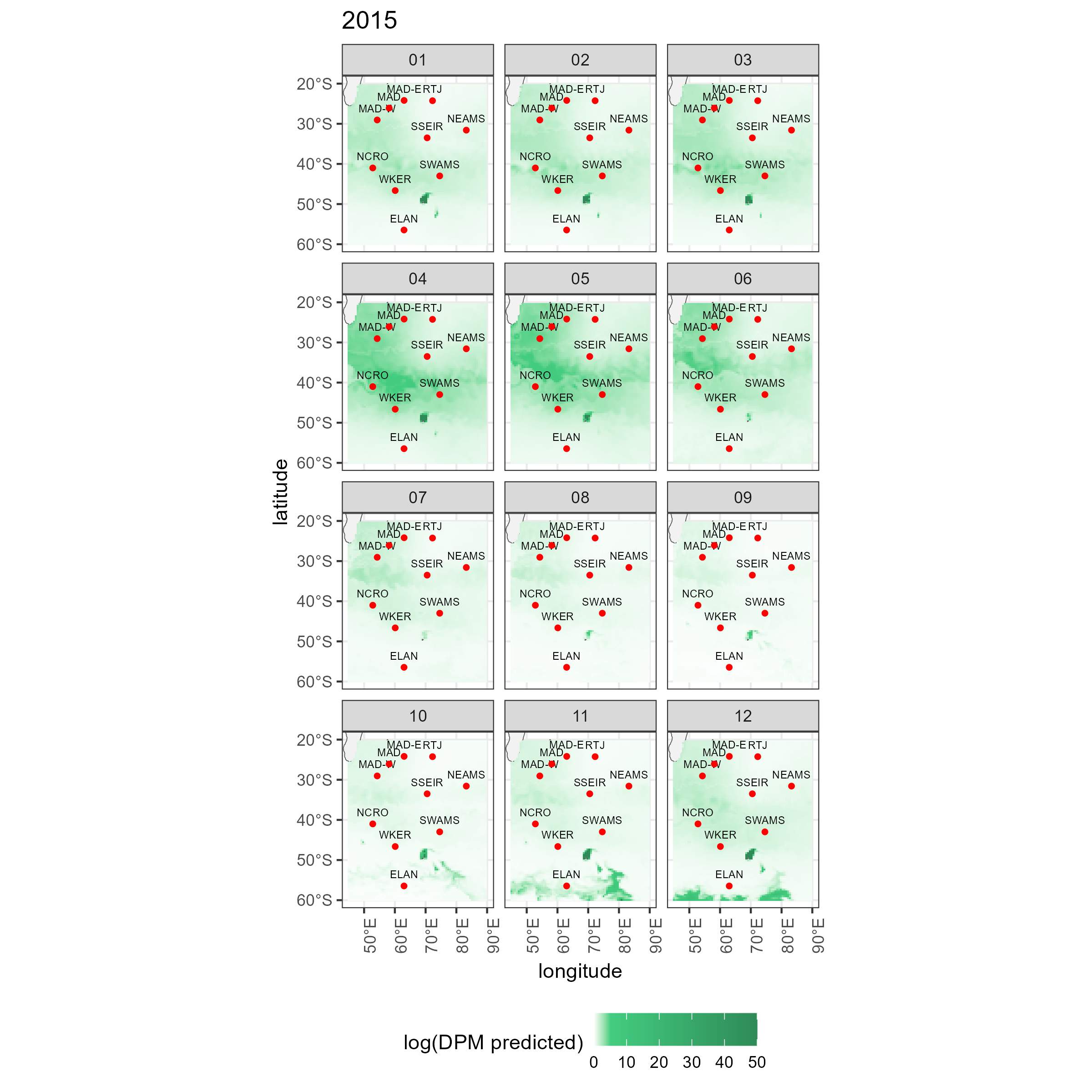

Supplement: Supplementary file 1 — Supplementary Information. [file 41598_2025_2941_MOESM1_ESM.zip › supp fig/swiopbw/plot_prediction_madpbw_2015.png]

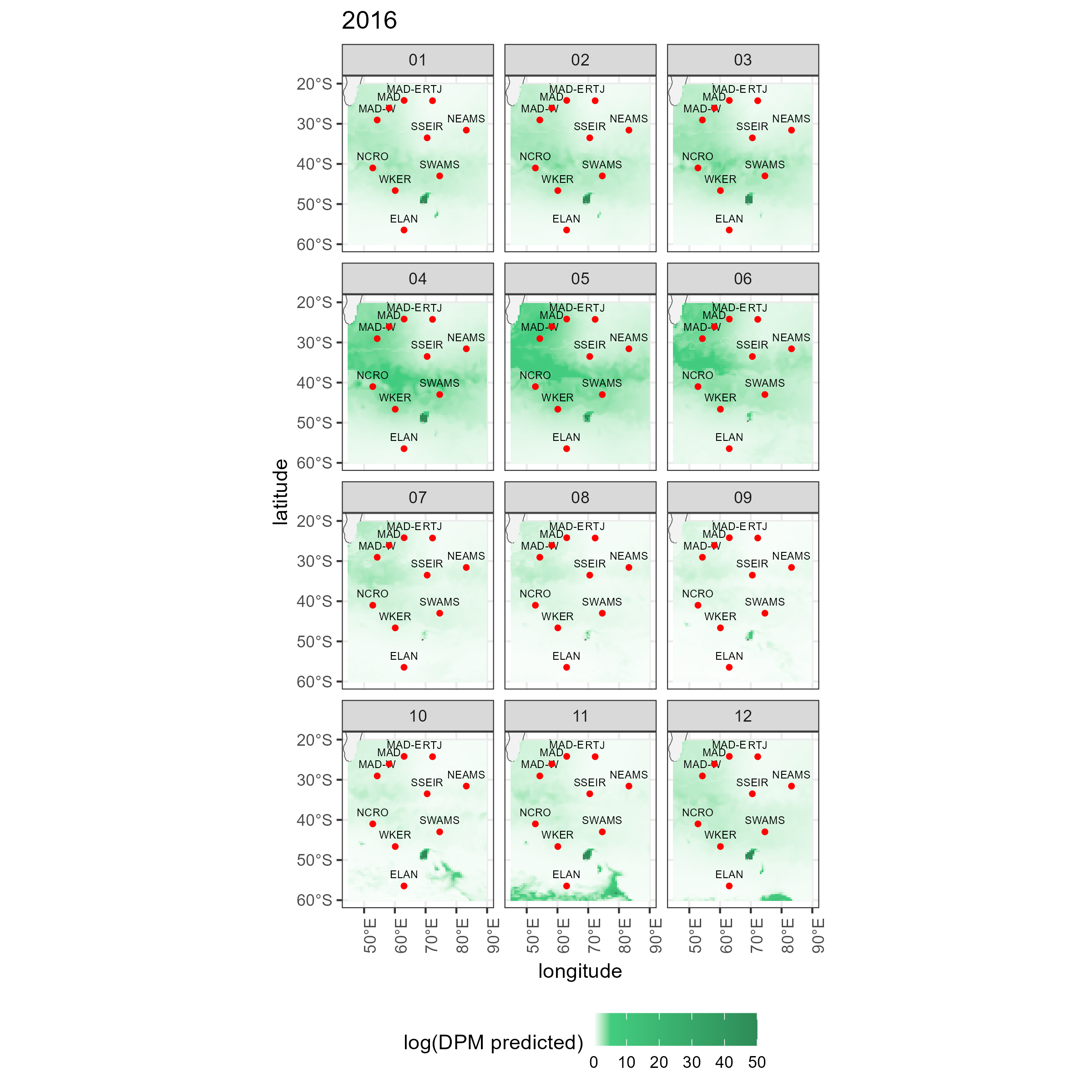

Supplement: Supplementary file 1 — Supplementary Information. [file 41598_2025_2941_MOESM1_ESM.zip › supp fig/swiopbw/plot_prediction_madpbw_2016.png]

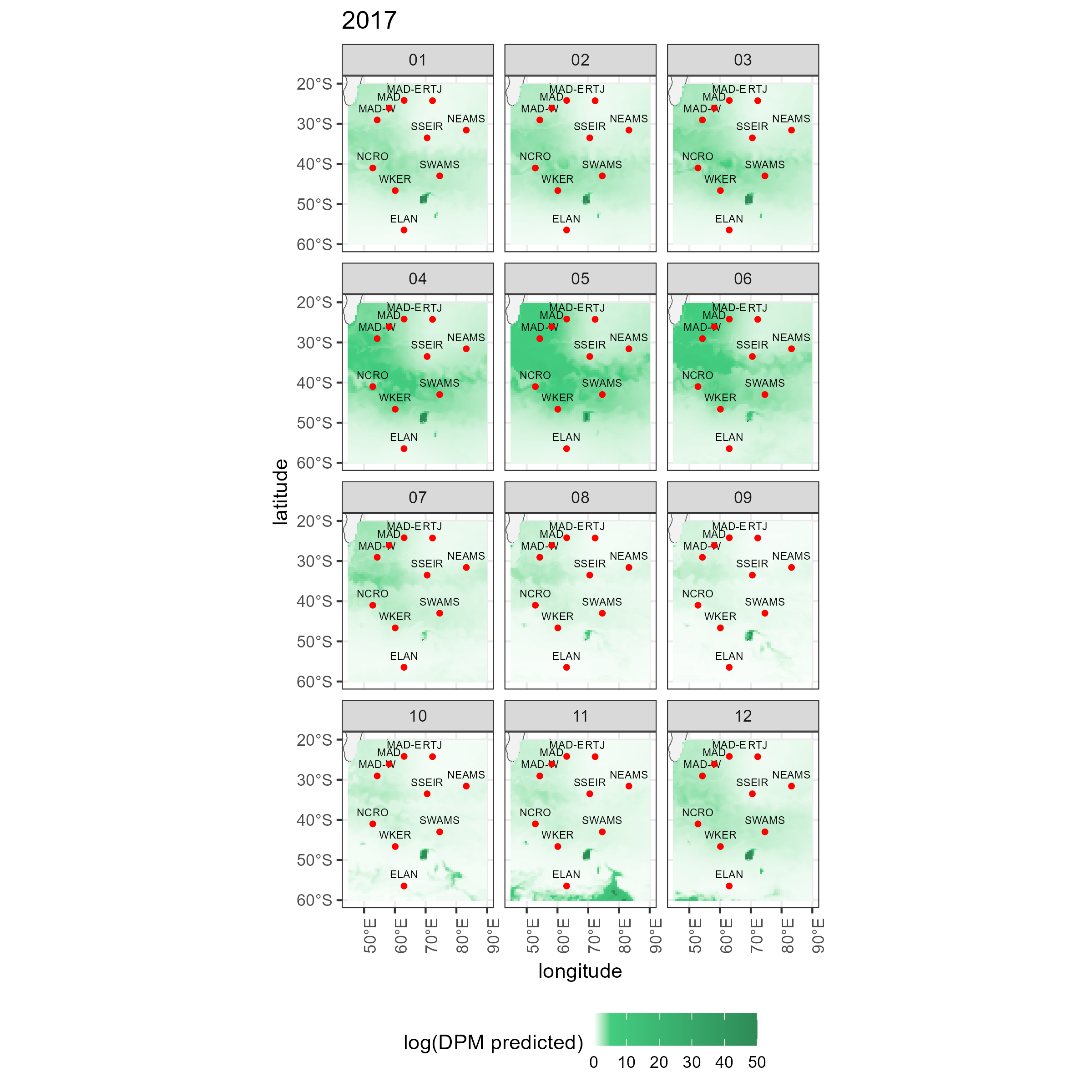

Supplement: Supplementary file 1 — Supplementary Information. [file 41598_2025_2941_MOESM1_ESM.zip › supp fig/swiopbw/plot_prediction_madpbw_2017.png]

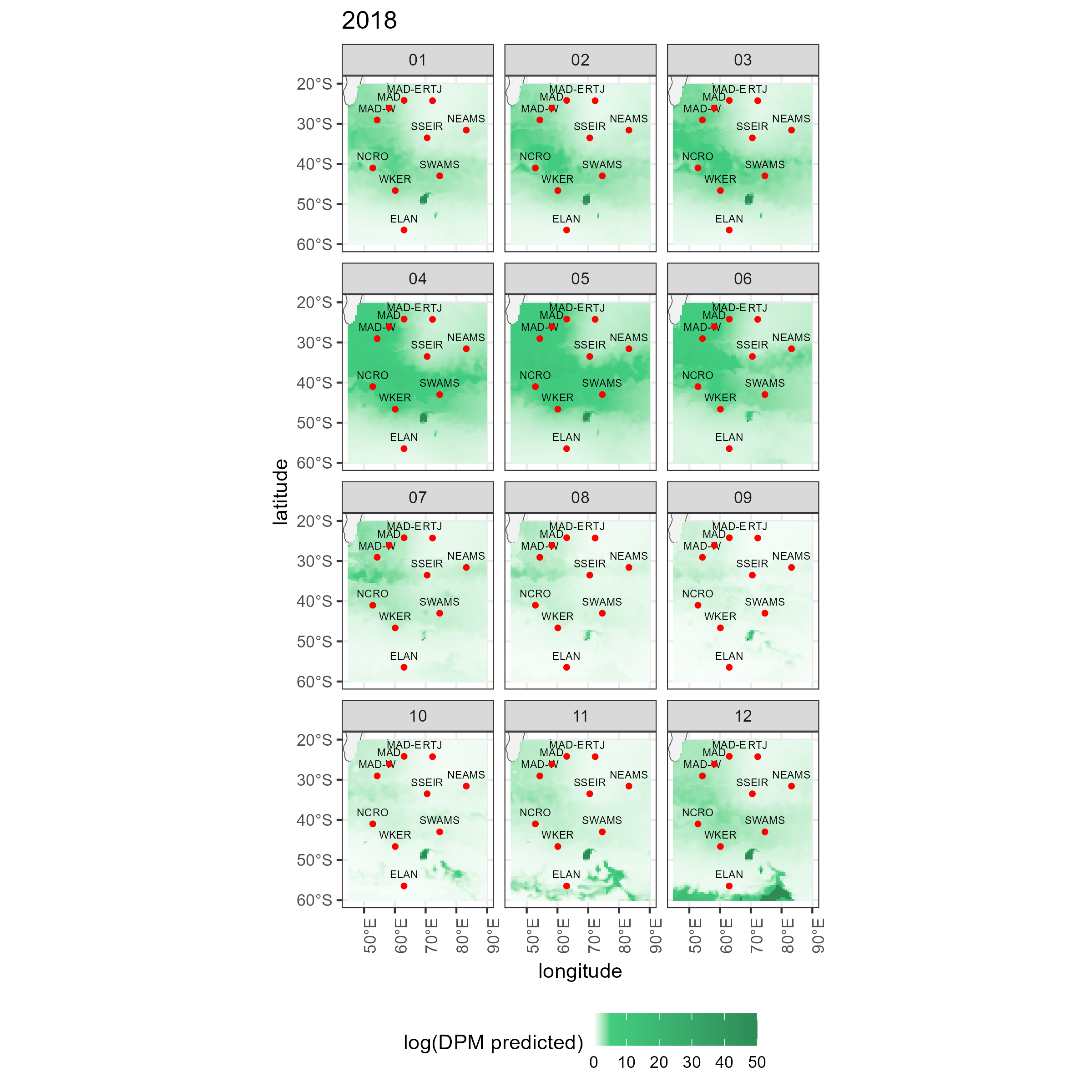

Supplement: Supplementary file 1 — Supplementary Information. [file 41598_2025_2941_MOESM1_ESM.zip › supp fig/swiopbw/plot_prediction_madpbw_2018.png]

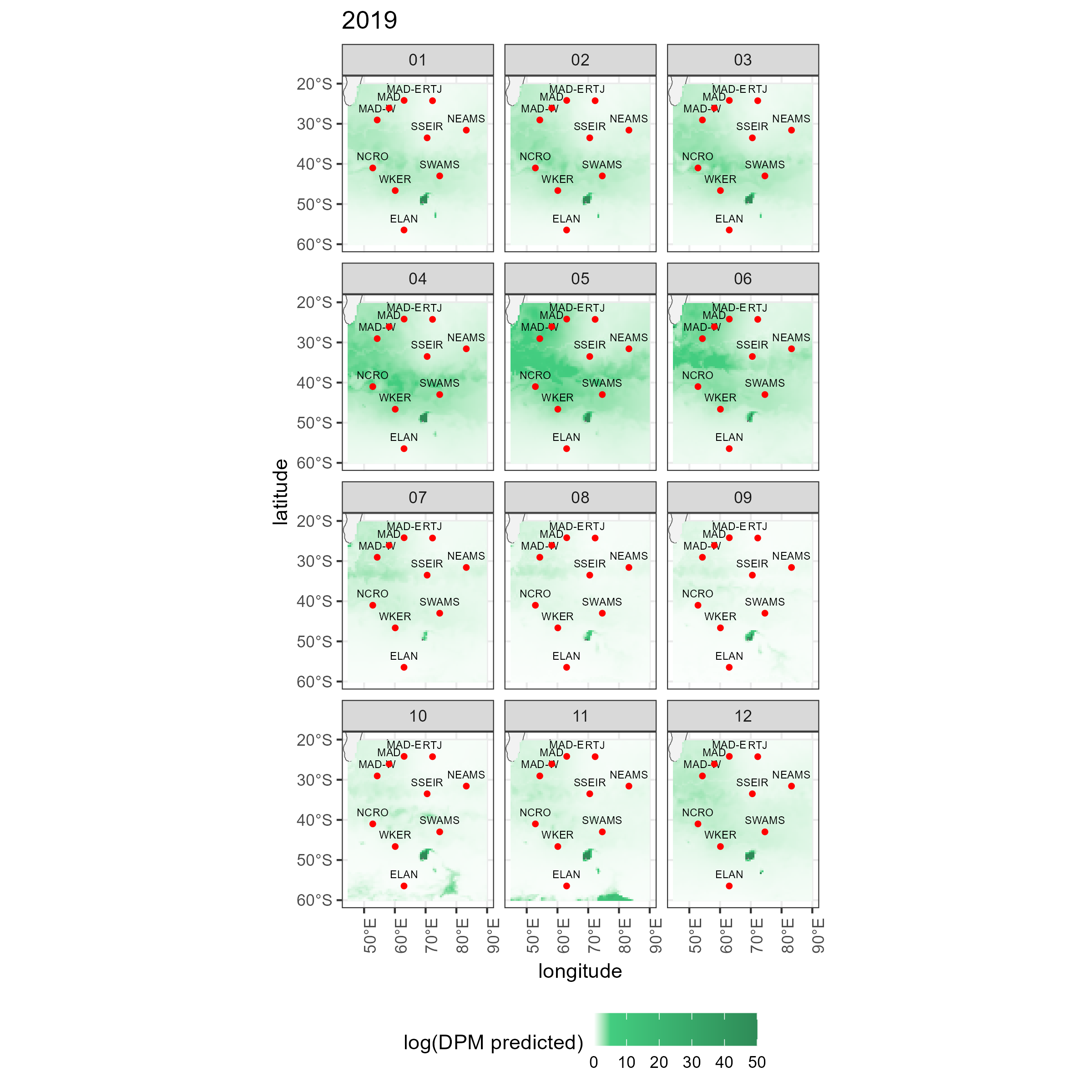

Supplement: Supplementary file 1 — Supplementary Information. [file 41598_2025_2941_MOESM1_ESM.zip › supp fig/swiopbw/plot_prediction_madpbw_2019.png]

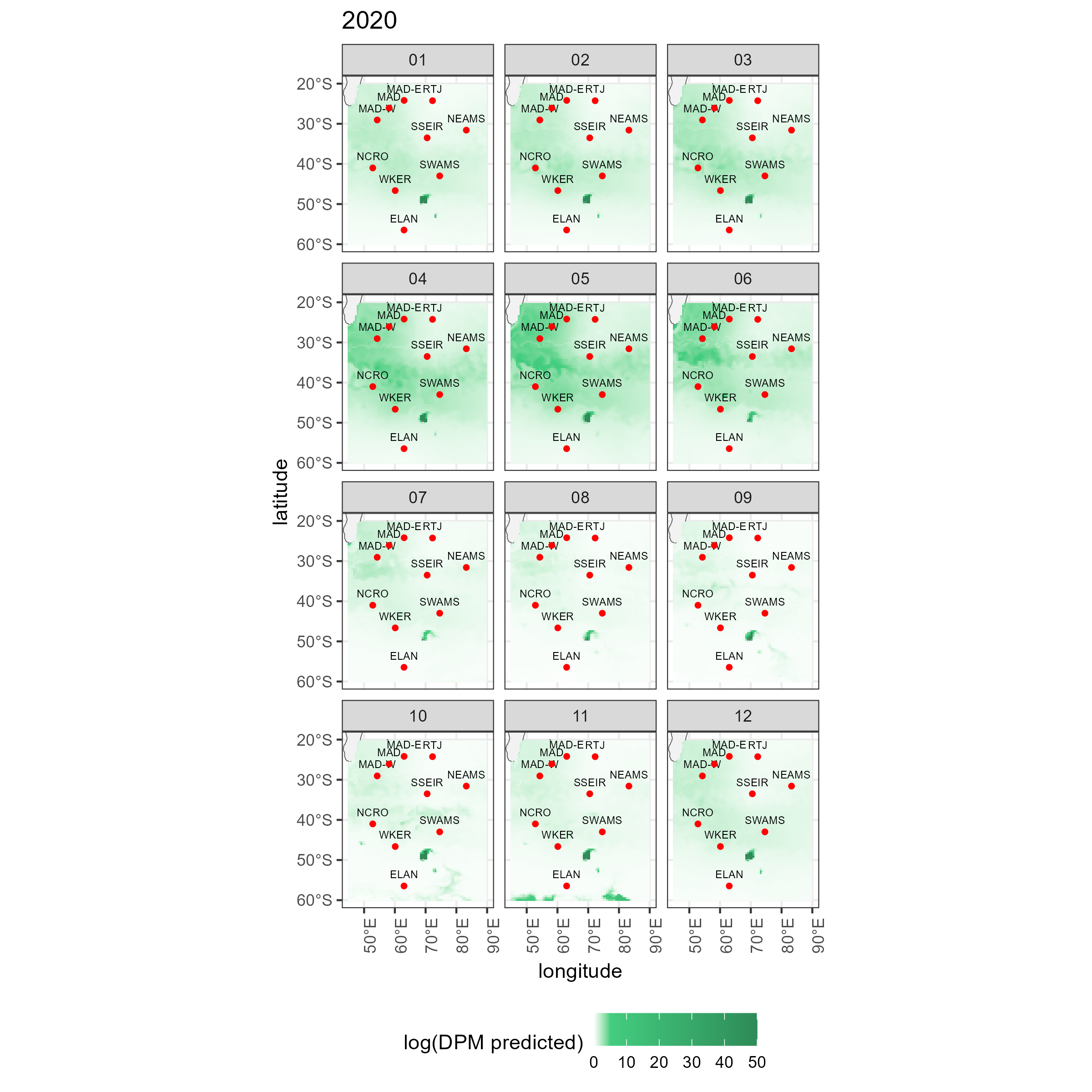

Supplement: Supplementary file 1 — Supplementary Information. [file 41598_2025_2941_MOESM1_ESM.zip › supp fig/swiopbw/plot_prediction_madpbw_2020.png]

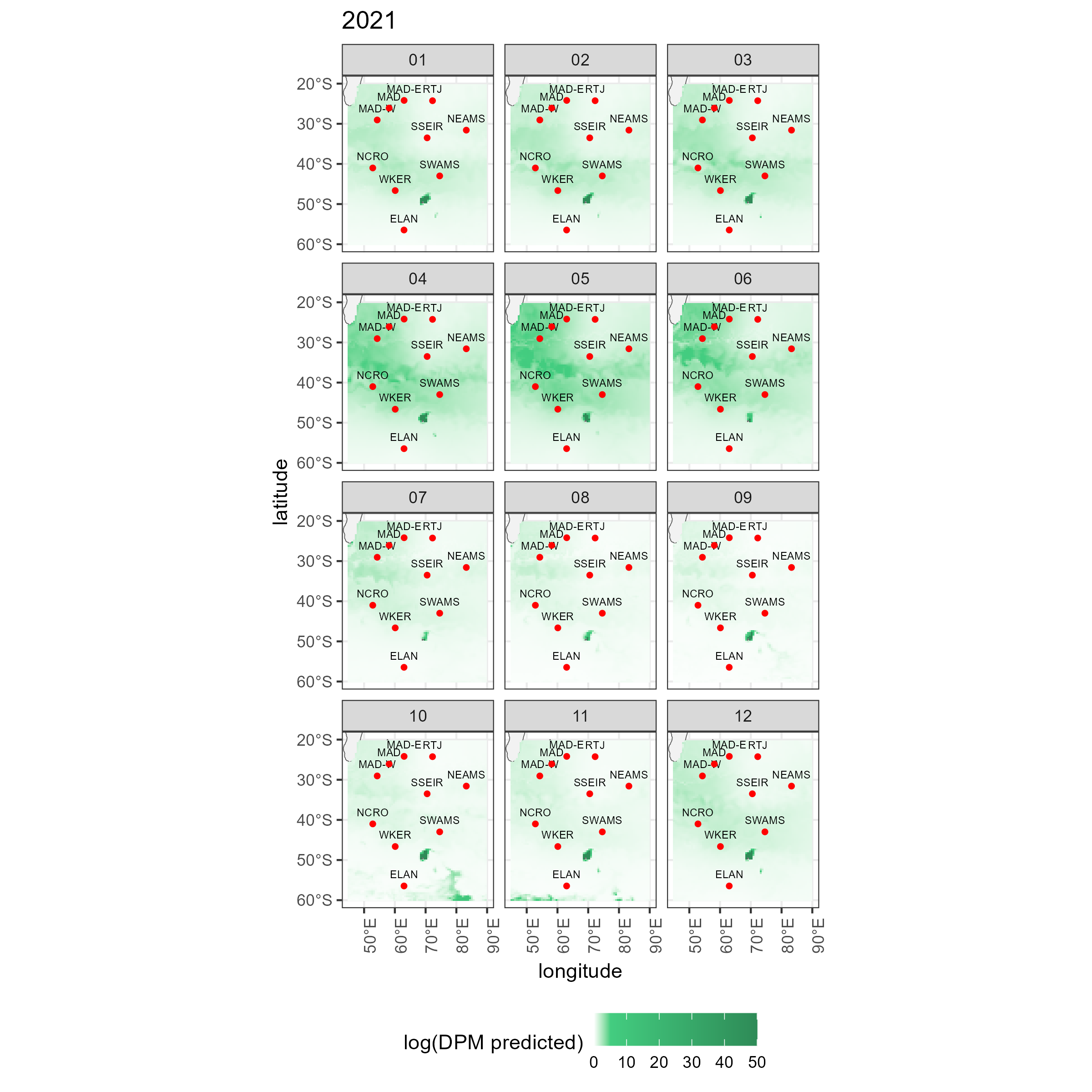

Supplement: Supplementary file 1 — Supplementary Information. [file 41598_2025_2941_MOESM1_ESM.zip › supp fig/swiopbw/plot_prediction_madpbw_2021.png]

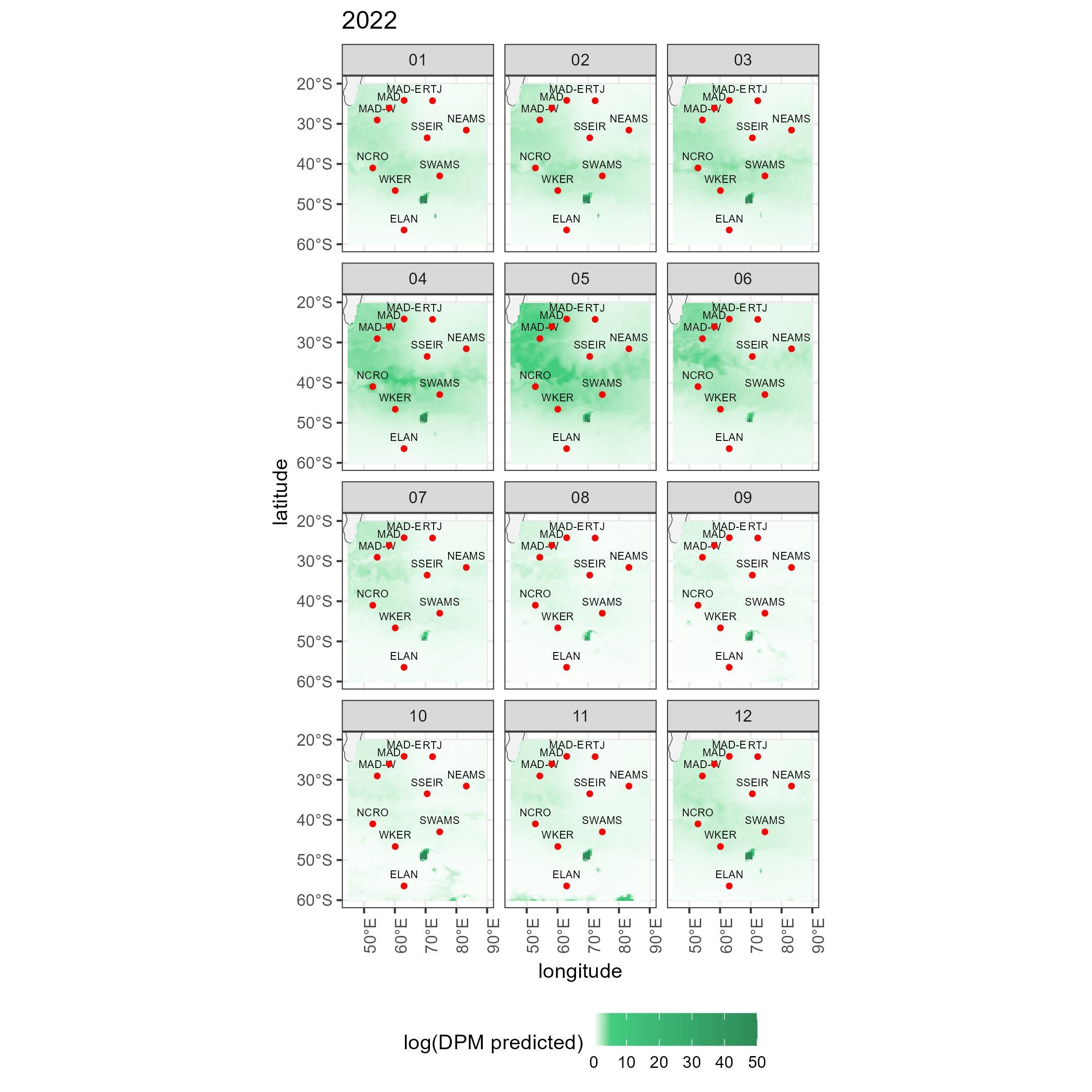

Supplement: Supplementary file 1 — Supplementary Information. [file 41598_2025_2941_MOESM1_ESM.zip › supp fig/swiopbw/plot_prediction_madpbw_2022.png]

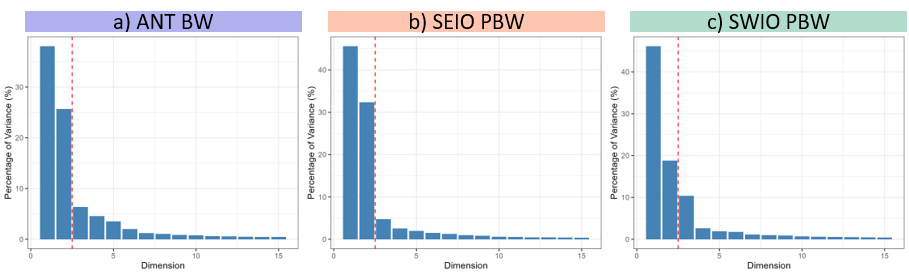

Supplement: Supplementary file 1 — Supplementary Information. [file 41598_2025_2941_MOESM1_ESM.zip › supp fig/variance explained EOF.png]
